# Unprecedented synthesis of a 14-membered hexaazamacrocycle

**DOI:** 10.3762/bjoc.19.126

**Published:** 2023-11-15

**Authors:** Anastasia A Fesenko, Anatoly D Shutalev

**Affiliations:** 1 N. D. Zelinsky Institute of Organic Chemistry, Russian Academy of Sciences, 47 Leninsky Ave., 119991 Moscow, Russian Federation

**Keywords:** amidrazones, hexaazamacrocycles, pyrazolo[3,4-*d*]pyrimidines, ring contraction, self-assembly

## Abstract

The transformation of 3-[(ethoxymethylene)amino]-1-methyl-1*H*-pyrazole-4-carbonitrile into the 14-membered macrocycle, 2,10-dimethyl-2,8,10,16-tetrahydrodipyrazolo[3,4-*e*:3',4'-*l*][1,2,4,8,9,11]hexaazacyclotetradecine-4,12-diamine, by the reaction with excess hydrazine under various conditions was studied in detail. The reaction proceeded through the initial formation of 4-imino-2-methyl-2,4-dihydro-5*H*-pyrazolo[3,4-*d*]pyrimidin-5-amine followed by dimerization to give the final macrocycle. A convenient synthesis of the latter starting from 4-imino-2-methyl-2,4-dihydro-5*H*-pyrazolo[3,4-*d*]pyrimidin-5-amine was developed. A plausible pathway for the macrocycle self-assembly is discussed. Some features of the structure and reactivity of the obtained macrocycle are outlined.

## Introduction

The chemistry of polyazamacrocycles (PAMs) is currently one of the most rapidly developing areas of heterocyclic chemistry [1–6]. The great interest in PAMs is primarily due to their ability to bind various cations, anions, and neutral molecules [7–14]. In addition, some representatives of PAMs were found in various natural products and play an important role in living systems (e.g., vitamin B_12_, chlorophyll, metalloproteins, cyclic peptides, etc). PAMs themselves and their metal complexes exhibit various useful properties [15–31], particularly, they possess a wide range of biological activities and are used as contrast agents for magnetic resonance imaging, radiopharmaceuticals, sensors, NMR shift reagents, luminescent materials, catalysis, etc.

To date, a large variety of PAMs with various ring sizes, number and location of nitrogen atoms, levels of unsaturation, etc. have been prepared and studied. Nevertheless, the synthesis of novel PAMs with interesting properties is of great importance. Recently, we developed some approaches to 14-membered cyclic bis-semicarbazones [32–35] and bis-thiosemicarbazone [36], namely 7,14-dimethyl-1,2,4,8,9,11-hexaazacyclotetradeca-7,14-diene-3,10-diones and -3,10-dithiones. The prepared compounds were able to chelate various metal cations through the N1, N4, N8, and N11 atoms [37–38]. In continuation of our research on 1,2,4,8,9,11-hexaazamacrocycles, which are under-explored representatives of the PAMs family, we were particularly interested in the synthesis of more unsaturated and therefore more conformationally rigid compounds. Previously, the unintentional preparation of two polyunsaturated 1,2,4,8,9,11-hexaazamacrocycles fused with two benzene or two pyrazole rings has been reported [39–40]. In particular, Dolzhenko et al. attempted to reproduce the synthesis of 4-imino-2-methyl-2,4-dihydro-5*H*-pyrazolo[3,4-*d*]pyrimidin-5-amine described by Baraldi et al. [41] using the reaction of 3-[(ethoxymethylene)amino]-1-methyl-1*H*-pyrazole-4-carbonitrile with excess hydrazine hydrate in EtOH under reflux. However, a pyrazole-fused 1,2,4,8,9,11-hexaazamacrocycle was unexpectedly obtained instead [40]. Since this type of macrocycle self-assembly seems to be very promising, we decided to reproduce the Dolzhenko’s procedure, then study the macrocyclization in detail, and extend this approach to the synthesis of other polyunsaturated 1,2,4,8,9,11-hexaazamacrocycles.

Herein, we report the detailed studies of the hydrazine-promoted transformation of 3-[(ethoxymethylene)amino]-1-methyl-1*H*-pyrazole-4-carbonitrile (**4**) or 4-imino-2-methyl-2,4-dihydro-5*H*-pyrazolo[3,4-*d*]pyrimidin-5-amine (**8**) into 2,10-dimethyl-2,8,10,16-tetrahydrodipyrazolo[3,4-*e*:3',4'-*l*][1,2,4,8,9,11]hexaazacyclotetradecine-4,12-diamine (**5**) under various conditions. Mechanistic aspects of the macrocyclization are also discussed. Some features of the structure and reactivity of the obtained macrocycle are outlined.

## Results and Discussion

**Scheme 1:**
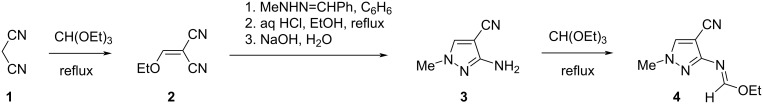
Synthesis of the key intermediate of the macrocycle preparation, 3-[(ethoxymethylene)amino]-1-methyl-1*H*-pyrazole-4-carbonitrile (**4**).

The readily available 3-amino-1-methyl-1*H*-pyrazole-4-carbonitrile (**3**) was used as the starting material. This compound was prepared according to the described regioselective method [42] based on the reaction of malononitrile with triethyl orthoformate followed by subsequent treatment of the obtained dinitrile **2** with benzaldehyde methyl hydrazone in benzene, conc. aqueous HCl in EtOH, and NaOH in water (Scheme 1). The key intermediate of the macrocycle preparation, imidate **4**, was synthesized using the reported procedure [43] by refluxing a solution of aminopyrazole **3** in triethyl orthoformate.

**Scheme 2:**
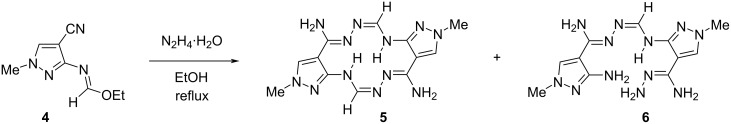
Synthesis of macrocycle **5** by the reaction of imidate **4** with hydrazine hydrate.

First, we studied the reaction of imidate **4** with hydrazine hydrate in EtOH under Dolzhenko’s conditions (4 equivalents of N_2_H_4_·H_2_O, concentration of **4** = 0.5 mmol/mL, reflux, 2 h) [40]. The resulting precipitate was isolated by filtration and washed with EtOH. In contrast to the reported data, the yield of the obtained product was significantly lower and did not exceed 38%. Moreover, according to NMR spectroscopic data, the isolated product was a mixture of the desired macrocycle **5** and a noticeable amount of an impurity (Scheme 2) whose formation was not mentioned in the cited reference.

The structure of the concomitant impurity was established using 1D and 2D NMR spectroscopy. The ^1^H NMR spectrum in DMSO-*d*_6_ shows the presence of two methylpyrazole moieties (singlet signals of two methyl groups at 3.63 and 3.70 ppm, singlet signals of two CH protons of pyrazole rings at 7.81 and 7.84 ppm), a H–N–C–H fragment with *trans*-orientation of protons (two doublets at 9.87 and 7.50 ppm, ^3^*J* = 11.2 Hz), four NH_2_ groups at 6.27, 5.72, 5.59, and 4.61 ppm (singlets). Signals of 11 different carbon atoms including 8 carbons of two methylpyrazole moieties were observed in the ^13^С NMR spectrum. Thus, we concluded that the impurity has bis-pyrazole structure **6**. This structure was also confirmed by the ^1^H,^13^C-HSQC and ^1^H,^13^C-HMBС spectra, as well as by comparing the experimental carbon chemical shifts in DMSO-*d*_6_ with those calculated for **6** by the GIAO method at the PBE1PBE/6-311+G(2d,p) level of theory using the DFT B3LYP/6-311++G(d,p) optimized geometries (DMSO solution) and applying a multi-standard approach [44] (see the Supporting Information File 1 for details). The high-resolution mass spectrum (ESI^+^) of a mixture of **5** and the impurity, in addition to a peak at *m*/*z* = 329.1696 [M + H]^+^ for compound **5**, shows a peak at *m*/*z* = 319.1862 [M + H]^+^, consistent with the molecular formula of C_11_H_18_N_12_ for bis-pyrazole **6**. According to NMR spectroscopic data, the amount of bis-pyrazole **6** in the crude product formed under above conditions was about 18 mol %.

The structure of macrocycle **5** was confirmed by comparing its ^1^H and ^13^C NMR spectra with those reported in ref. [40]. It should be noted that the ^1^H and ^13^C{1H} NMR spectra of compound **5** in DMSO-*d*_6_ show only a half-number set of proton or carbon signals (five and six signals, respectively), thus indicating its C_2_-symmetric dimeric structure. The analysis of 2D NMR spectroscopic data provided additional evidence for the macrocycle **5** structure (see Supporting Information File 1). The high-resolution mass spectrum (ESI^+^) confirmed its chemical formula as C_12_H_16_N_12_.

Thus, we found that, in contrast to the reported data [40], the reaction between imidate **4** and hydrazine hydrate (4 equiv) in refluxing EtOH for 2 h afforded macrocycle **5** in a relatively low yield, along with an appreciable amount of the byproduct **6**. This prompted us to optimize the reaction conditions varying hydrazine hydrate excess (from 3.1 to 4.3 equivalents), solvent (EtOH, MeOH, 1,4-dioxane, DME), reaction time (2 h and 6 h), and also using anhydrous hydrazine instead of hydrazine hydrate. However, all our attempts to improve both the yield and the purity of **5** failed. For example, prolonging the reaction time between **4** and N_2_H_4_∙H_2_O (4 equiv) in refluxing EtOH to 6 h resulted in an increase in the purity of the macrocycle (**5**/**6** = 91:9), but simultaneously to a decrease in its yield to 25%. Reducing the amount of hydrazine hydrate to 3 equivalents (EtOH, reflux, 2 h) had a similar effect and gave an 85:15 mixture of **5** and **6** in an overall yield of 22%. In refluxing MeOH (3 equiv of N_2_H_4_∙H_2_O, 2 h), a mixture of **5** and **6** in a ratio of 89:11 was obtained in 31% overall yield. In aprotic solvents (1,4-dioxane or DME), the selectivity of the reaction dramatically decreased and a mixture of **5** and **6** along with significant amounts of various unidentified byproducts was formed. For example, the reaction of **4** with N_2_H_4_∙H_2_O (4.1 equiv) in refluxing 1,4-dioxane for 2 h, followed by evaporation of the volatiles under reduced pressure, afforded a complex mixture containing only 7 mol % of macrocycle **5** according to the ^1^H NMR spectrum with the addition of a weighted amount of succinimide as a reference. Analogously, only 2 mol % of **5** were detected under the above conditions (1,4-dioxane, reflux, 2 h) when anhydrous hydrazine (4.2 equiv) was used as a promoter.

**Scheme 3:**
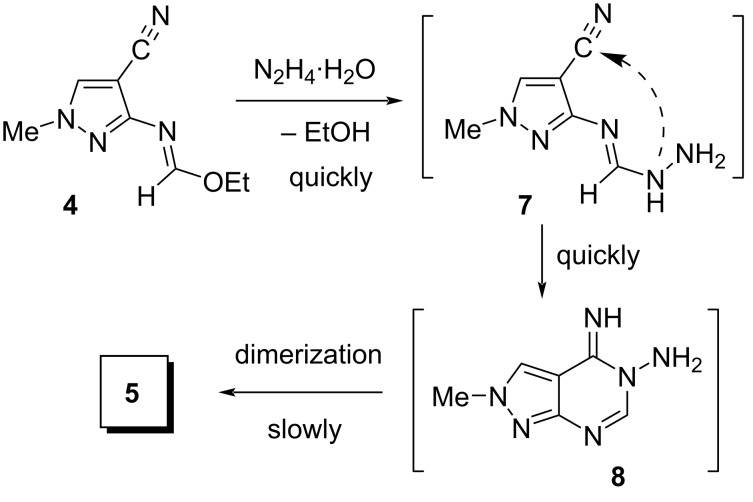
Plausible pathway for the transformation of imidate **4** into macrocycle **5**.

A plausible pathway for the transformation of imidate **4** into macrocycle **5** is shown in Scheme 3. This pathway includes fast substitution of the ethoxy group by hydrazine to give the intermediate amidrazone **7** followed by its rapid conversion to pyrazolopyrimidine **8**. Slow dimerization of compound **8** results in macrocycle **5**.

The formation of **5** through pyrazolopyrimidine **8** is confirmed by the literature data [40] that the reaction of **4** with N_2_H_4_∙H_2_O in EtOH to give **8** proceeds under much milder conditions than the reaction to afford **5** (rt and reflux, respectively). Based on this background, we assumed that the synthesis of macrocycle **5** could be carried out directly from **8**. We also hoped that this would be especially useful from a preparative viewpoint, since pure pyrazolopyrimidine **8** can be easily obtained in any required quantities, in contrast to pure imidate **4**.

**Scheme 4:**
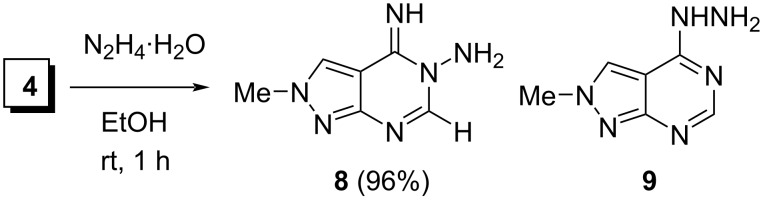
Synthesis of pyrazolopyrimidine **8** by the reaction of imidate **4** with hydrazine hydrate.

Pyrazolopyrimidine **8** was prepared by the reaction of **4** with N_2_H_4_∙H_2_O in EtOH according to our modification of the described procedure [40] using room temperature (without pre-cooling), a lower excess of N_2_H_4_∙H_2_O (1.6 equiv instead of 5 equiv) and a shorter reaction time (1 h instead of 5 h). The precipitated compound **8** was isolated by filtration in a 96% yield (Scheme 4).

Previously, the structure of **8** was assigned based on ^1^H and ^13^C NMR spectroscopic data [40]. However, these data are insufficient to distinguish compound **8** and its isomer **9** resulting from a Dimroth rearrangement that is known to proceed in 3-substituted 4-iminopyrimidine systems [40,45–47]. Our analysis of ^1^H, ^13^C NMR, and 2D NMR spectra (DMSO-*d*_6_ solution) of the prepared product confirmed its structure as compound **8**. For example, the ^1^H,^13^C-HMBC spectrum showed correlation of the NH_2_ protons with carbon C-6 (through three bonds), and the ^1^H,^1^H-NOESY experiment revealed a diagnostic NOE between the NH_2_ and H-6 protons. The structure **8** was also confirmed by comparing the experimental carbon chemical shifts of the prepared compound in DMSO-*d*_6_ with shifts calculated for **8** and **9** by the GIAO method at the PBE1PBE/6-311+G(2d,p) level of theory using the DFT B3LYP/6-311++G(d,p) optimized geometries (DMSO solution) and applying a multi-standard approach [44]. The calculated shifts of sp^2^-atoms C-7a, C-4, C-6, C-3, and C-3a in (*Z*)-**8** and the s-*cis*-conformer (with respect to the C4–N bond) of **9** were 156.6, 152.1, 151,9, 128.3, 105.6 ppm and 160.9, 158.9, 157.4, 123.3, 98.4 ppm, respectively. The corresponding experimental shifts (155.0, 151.8, 149.9, 128.4, 105.4 ppm) were in good agreement with the structure **8**. It is noteworthy that the DFT B3LYP/6-311++G(d,p) calculations using the PCM solvation model showed that (*Z*)-**8** was significantly less stable than the s-*cis*-conformer of **9** in DMSO solution (Δ*G* = 7.17 kcal/mol; 298 K, 1 atm).

**Scheme 5:**
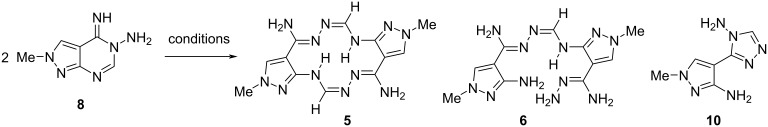
Synthesis of macrocycle **5** by the dimerization of pyrazolopyrimidine **8**.

As we proposed, pyrazolopyrimidine **8** undergoes dimerization to produce macrocycle **5** under certain conditions (Scheme 5).

The dimerization of **8** was thoroughly studied varying promoter, its amount, solvent, substrate concentration, and reaction time (Table 1).

**Table 1:** Synthesis of macrocycle **5** by the dimerization of pyrazolopyrimidine **8**.^a^

Entry	Promoter (equiv)	Solvent	Conc. of **8** (mmol/mL)	Reaction time (h)	Isolated products^b^	Mass yield of products (%)	Molar ratio of products^c^	Estimated yield of **5** (%)^d^

1	N_2_H_4_·H_2_O (1.02)	EtOH	0.39	2	**5** + **6** + **8**	18	56:7:37	13
2	N_2_H_4_·H_2_O (3.11)	EtOH	0.46	2	**5** + **6**	35	80:20	28
3	N_2_H_4_·H_2_O (6.29)	EtOH	0.34	0.5	**5** + **6**	21	64:36	13
4	N_2_H_4_·H_2_O (1.90)	THF	0.33	2	**5** + **6** + **8**	60	3:4:93	3
5	N_2_H_4_·H_2_O (2.10)	dioxane	0.20	2	**5** + **6**^e^	46	89:11	≈41
6	N_2_H_4_·H_2_O (3.09)	dioxane	0.33	2	**5** + **6**	41	84:16	34
7	N_2_H_4_·H_2_O (2.03)	dioxane	1.00	2	**5** + **6**^f^	47	76:24	–
8	N_2_H_4_·H_2_O (1.44)	dioxane	0.33	2	**5** + **6** + **8**^e^	43	54:9:37	≈29
9	N_2_H_4_·H_2_O (3.07)	pyridine	0.50	1.5	**5** + **6**	18	86:14	15
10	N_2_H_4_·H_2_O (2.20)	pyridine	0.33	1.5	**5** + **6**	16	89:11	14
11	N_2_H_4_·H_2_O (1.99)	MeCN	0.33	2	**5** + **6**^e^	34	56:44	≈19
12	N_2_H_4_·H_2_O (6.19)	iPrOH	0.31	1.5	**5** + **6**	15	58:42	9
13	N_2_H_4_·H_2_O (1.46)	MeOH	0.50	3	**5** + **6**	41	89:11	36
14	N_2_H_4_·H_2_O (2.00)	MeOH	0.50	3	**5** + **6**	40	89:11	36
15	N_2_H_4_·H_2_O (6.04)	MeOH	0.34	1	**5** + **6**	20	82:18	17
16	N_2_H_4_·H_2_O (6.05)	EtOH/H_2_O 12:1 (v/v)	1.03	1	**5** + **6**	21	81:19	17
17	N_2_H_4_·H_2_O (3.07)	dioxane/H_2_O 12:1 (v/v)	1.03	1	**5** + **6**	21	82:18	17
18	N_2_H_4_ (2.21)	MeOH	0.50	2	**5** + **6**	38	87:13	33
19	MeNHNH_2_ (2.63)	EtOH	0.38	2	**5** + **6**^f^	22	91:9	–
20	N_2_H_4_·H_2_O (1.51)	MeOH	0.48	3	**5** + **6**	43	90:10	39
21	N_2_H_4_·H_2_O (1.51)	MeOH	0.50	3	**5** + **6**	46	89:11	41
22	N_2_H_4_·H_2_O (3.04)+ TsOH·H_2_O (0.05)	EtOH	0.50	1	**5** + **6** + **10**	38	66:4:30	29
23	N_2_H_4_·H_2_O (2.93)+ TsOH·H_2_O (0.05)	dioxane	0.51	1	**5** + **6** + **10**	87	13:0.5:86.5	19
24	TsOH·H_2_O (0.10)	dioxane	0.50	1	**8**	98	–	0
25	TsOH·H_2_O (0.10)	MeCN	0.24	2	**5** + **8**^f^	91	0.8:99.2	<1
26	TsOH·H_2_O (0.10)	MeOH	0.38	5.5	**5**	4	–	4
27	TsOH·H_2_O (0.10)	EtOH	0.37	2	**5** ^f^	–	–	12^g^

^a^Loadings of **8** were 50–141 mg (0.30–0.86 mmol) in entries 1–20, 23, 26, 27, 1.016 g (6.19 mmol) in entry 21, 4.039 g (24.60 mmol) in entry 22, 0.337 g (2.05 mmol) in entry 24, and 0.315 g (1.92 mmol) in entry 25. All reactions were carried out under refluxing conditions; ^b^methods of the product isolation: (i) filtration of the precipitate formed (for entries 1, 2, 4–8, 10, 11, 13–21, 24–26), (ii) evaporation of the volatiles under vacuum followed by treatment with water and filtration of the formed precipitate (for entries 3, 9, 12, 22, 23), (iii) evaporation of the volatiles under vacuum (for entry 27); ^c^according to ^1^H NMR spectrum of the crude product; ^d^calculated based on overall mass yields and molar ratios of the products; ^e^plus a small amount of unidentified impurities; ^f^plus a significant amount of unidentified impurities; ^g^the yield was estimated by ^1^H NMR spectrum for a mixture of the crude product with a weighed amount of succinimide as a reference.

First, we studied the dimerization of **8** promoted by hydrazine hydrate in EtOH under reflux (Table 1, entries 1–3). We found that the starting material was completely consumed in the presence of 3 equivalents of N_2_H_4_·H_2_O within 2 h and the precipitated solid was isolated by filtration. According to the ^1^H NMR spectrum, this crude product was a mixture of macrocycle **5** and bis-pyrazole **6** in a molar ratio of 80:20 (Table 1, entry 2). An increase in the amount of N_2_H_4_·H_2_O to 6.3 equivalents led to a faster conversion of **8**, however, the amount of bis-pyrazole **6** in the isolated mixture increased to 36% (Table 1, entry 3). In contrast, reducing the amount of N_2_H_4_·H_2_O to 1 equivalent resulted in an increase in the **5**:**6** ratio to 89:11 and a decrease in the conversion of **8** to 63% after 2 h of reflux (Table 1, entry 1).

Next, we tested other protic (iPrOH, MeOH, EtOH/H_2_O, 1,4-dioxane/H_2_O) and aprotic (THF, 1,4-dioxane, pyridine, MeCN) solvents for the dimerization of **8** promoted by hydrazine hydrate to improve both yield and purity of **5** (Table 1, entries 4‒17). As can be seen from Table 1 the solvent had a dramatic effect on the outcome of the reaction. With THF, pyridine, MeCN, iPrOH, EtOH/H_2_O, or 1,4-dioxane/H_2_O either low conversion of **8** (Table 1, entry 4), or poor product yield (entries 9, 10, 12, 16, and 17), or low purity of **5** (entries 11 and 12) were observed. Using 1,4-dioxane with 3 equivalents of N_2_H_4_·H_2_O (reflux, 2 h), a mixture of **5** and **6** in a molar ratio of 84:16 was obtained, and the calculated yield of **5** was 34% (Table 1, entry 6). The best result was achieved by the reaction of **8** with 1.5–2 equivalents of N_2_H_4_·H_2_O in MeOH under reflux for 3 h (**5**/**6** = 89:11, macrocycle calculated yield of 36%) (entries 13 and 14 in Table 1).

The experimental data described above were obtained using 50–141 mg of pyrazolopyrimidine **8**. We demonstrated that, under the optimized conditions (1.5 equiv of N_2_H_4_·H_2_O, MeOH, reflux, 3 h), the reaction can be scaled up to gram quantities without any loss of efficiency and even with a noticeable increase in the macrocycle yield up to 41% (Table 1, entries 20 and 21). The extremely poor solubility of product **5** in most organic solvents allowed to purify it from all admixtures, including byproduct **6**, by a single crystallization from boiling DMF. Thus, pure product **5** was prepared on a multi-gram scale in a 35% isolated yield.

Several other promoters were also tested to dimerize pyrazolopyrimidine **8**. In particular, the reaction proceeded in MeOH (reflux, 2 h) in the presence of anhydrous N_2_H_4_ (2.2 equiv) afforded product **5**, but in somewhat lower yield and purity compared with N_2_H_4_·H_2_O (entry 18 in Table 1). The dimerization of **8** in refluxing EtOH promoted by methylhydrazine gave a 91:9 mixture of **5** and **6** along with a significant amount of unidentified side-products (Table 1, entry 19). In the presence of TsOH·H_2_O (0.1 equiv) in refluxing MeOH or EtOH, macrocycle **5** was formed in unacceptably low yields (Table 1, entries 26 and 27), however, a complete conversion of the starting material was observed. In contrast, in aprotic solvents (1,4-dioxane, MeCN) in the presence of TsOH·H_2_O (0.1 equiv), the starting material remained intact (Table 1, entries 24 and 25). The use of N_2_H_4_·H_2_O (2.9–3.0 equiv) with a catalytic amount of TsOH·H_2_O (0.05 equiv) in refluxing EtOH or 1,4-dioxane resulted in the formation of mixtures of macrocycle **5**, pyrazolyl-1,2,4-triazole **10**, and a very small amount of bis-pyrazole **6** according to NMR data (Table 1, entries 22 and 23). It is noteworthy that triazole **10** was the major product in dioxane (**10**/**5**/**6** = 86.5:13:0.5, Table 1, entry 23). Again, macrocycle **5** was separated from this mixture by crystallization from DMF, and pyrazolyl-1,2,4-triazole **10** was isolated from the mother liquor.

The structure of compound **10** was established based on 1D and 2D NMR spectroscopic data. The ^1^H NMR spectrum in DMSO-*d*_6_ showed signals of two amino groups at 6.15 and 5.46 ppm. The HMBC spectrum demonstrated diagnostic cross peaks between one of these groups (6.15 ppm) and carbons at 148.8 ppm (C) and 143.9 ppm (CH), and the other group (5.46 ppm) correlated with carbons at 154.1 ppm (C) and 129.1 ppm (CH). In the ^1^H,^1^H-NOESY experiment, NOEs were observed between the NH_2_ group at 6.15 ppm and two CH protons (8.28 and 8.12 ppm). Additionally, the structure **10** was confirmed by comparing its experimental carbon chemical shifts in DMSO-*d*_6_ with shifts calculated by the GIAO method at the PBE1PBE/6-311+G(2d,p) level of theory using the DFT B3LYP/6-311++G(d,p) optimized geometries (DMSO solution) and applying a multi-standard approach [44]. It is noteworthy that, in the most stable conformer of **10**, the NH_2_ group of the triazole ring is located between the pyrazole and triazole ring protons providing the observed NOEs.

**Scheme 6:**
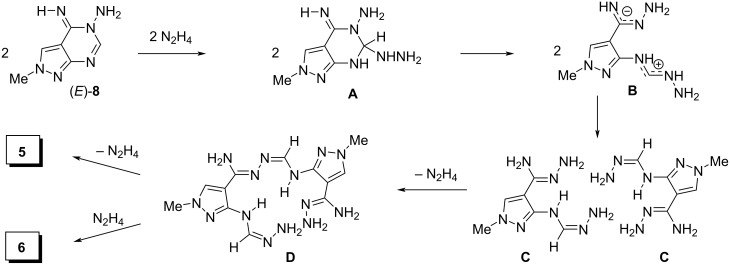
Plausible pathway for the dimerization of pyrazolopyrimidine **8** into macrocycle **5**.

To the best of our knowledge, self-assembly of compound **8** into macrocycle **5** seems to be unusual and a plausible pathway of this reaction promoted by hydrazine is shown in Scheme 6.

The first step involves a nucleophilic attack of hydrazine on the most electrophilic C2 carbon of the pyrimidine ring in **8** to give intermediate **A**. Cleavage of the C2–N3 bond in the latter followed by proton transfer in the formed zwitterion **B** affords bis-amidrazone **C**. Next, two molecules of bis-amidrazone **C** react with each other forming adduct **D**, which either cyclizes to macrocycle **5** or undergoes hydrazinolysis to give bis-pyrazole **6** as the byproduct. The independent formation of bis-pyrazole **6** and macrocycle **5** was confirmed by refluxing an 81:19 mixture of **5** and **6** with N_2_H_4_·H_2_O (4.0 equiv) in 1,4-dioxane for 2 h followed by removal of the volatiles under reduced pressure to obtain a mixture of **5** and **6** in only a slightly changed ratio (84:16 according to the ^1^H NMR spectrum).

It should be noted that Scheme 6 also explains formation of pyrazolyl-1,2,4-triazole **10** by the reaction of **8** with N_2_H_4_·H_2_O in the presence of TsOH·H_2_O (Table 1, entries 22 and 23). Under these conditions, the cleavage of either the C2–N3 bond or the N1–C2 bond can proceed. In the latter case, the formed 3-amino-*N*-(hydrazonomethyl)-1-methyl-1*H*-pyrazole-4-carboximidohydrazide recyclizes to give compound **10**.

**Figure 1:**
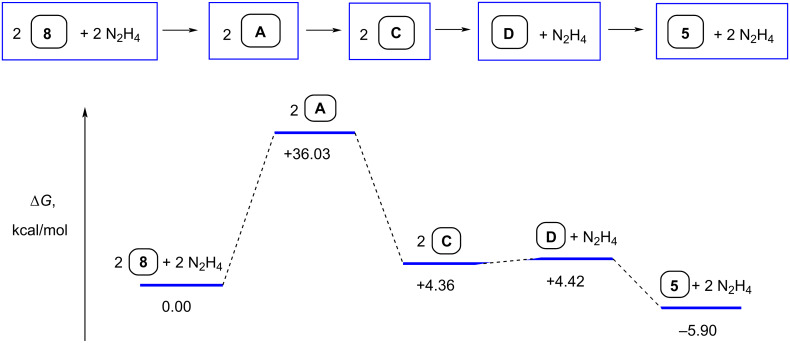
Gibbs free energy diagram (B3LYP/6-311++G(d,p)) for the N_2_H_4_-promoted transformation of pyrazolopyrimidine (*E*)-**8** into macrocycle **5** in MeOH solution. Free energies in kcal/mol at 298 K and 1 atm.

Thermodynamic parameters for the hydrazine-promoted transformation of pyrazolopyrimidine **8** into macrocycle **5** in MeOH solution were estimated by the DFT B3LYP/6-311++G(d,p) calculations. Relative Gibbs free energies of the starting, final, and intermediate molecular systems (Figure 1) were calculated using the Gibbs free energies for the most stable isomers of **8**, macrocycle **5**, intermediates **A**, **C**, **D**, and hydrazine.

Figure 1 shows that the hydrazine-promoted transformation of pyrazolopyrimidine **8** into macrocycle **5** in MeOH is a thermodynamically favorable process. Moreover, the extremely low solubility of macrocycle **5** makes the dimerization of **8** even more preferable.

It is noteworthy that the geometry of the intermediate bis-amidrazone **C** facilitates macrocycle **5** formation. The DFT B3LYP/6-311++G(d,p) calculations of ten different tautomers and stereoisomers of bis-amidrazone **C** (DMSO and MeOH solutions) revealed that the structure shown in Scheme 6 is the most stable. Clearly, the geometry of this structure favors the formation of adduct **D** and then macrocycle **5**. All these data explain the formation of macrocycle **5** from pyrazolopyrimidine **8** in acceptable yields (see Table 1), despite the multistep transformation (Scheme 6), rather harsh reaction conditions (>1.5 equivalents of N_2_H_4_·H_2_O, boiling solvent), and the presence of various reactive centers in both the starting material and the intermediate products.

Thus, we developed a preparative protocol for the synthesis of macrocycle **5** by the dimerization of pyrazolopyrimidine **8** in the presence of N_2_H_4_·H_2_O (1.5 equiv) (MeOH, reflux, 3 h) followed by crystallization of the crude product from DMF. The use of compound **8** as a starting material affords the target product **5** with higher purity and yield compared with the use of imidate **4**. Moreover, pyrazolopyrimidine **8** itself is much more stable than imidate **4**, and its intermediate isolation allows to remove residual impurities that arose from long-time reflux of aminopyrazole **3** in triethyl orthoformate (see the Experimental section).

According to the DFT B3LYP/6-311++G(d,p) calculations in DMSO solution, the polyunsaturated 14-membered ring in **5** is almost planar with a maximum atom deviation of 0.054 Å from the mean-square plane. The nuclear-independent chemical shift (NICS) [48–50] values of **5** in its optimized conformation were used as a magnetic criterion of aromaticity. The NICS(0) value for the macrocyclic ring (+2.23 ppm in DMSO), calculated at the HF/6-31+G(d) level, shows its somewhat anti-aromatic character. Obvious aromaticity of the pyrazole rings is confirmed by the NICS(0) values of −12.64 ppm.

**Scheme 7:**
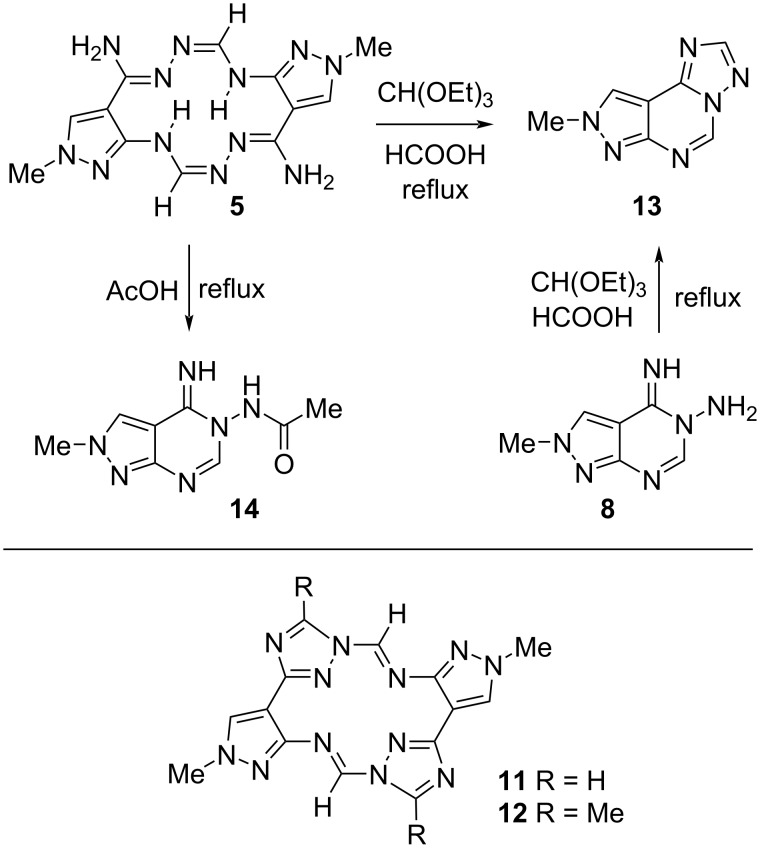
Transformation of macrocycle **5** into pyrazolo[4,3-*e*][1,2,4]triazolo[1,5-*c*]pyrimidine **13** and pyrazolopyrimidine **14**.

Macrocycle **5** has various reactive centers, including two amidrazone fragments with primary amino groups, which makes it possible to modify it in many ways [51–54]. Moreover, the size of internal cavity formed by the nitrogen atoms N5, N8, N13, and N16 (≈2.6 Å × 2.8 Å according to the DFT calculations) enables to chelate various metal cations. Herein, we attempted to prepare compounds **11** and **12** by annulation of two 1,2,4-triazole rings onto the macrocyclic core of **5** using acylation reactions (Scheme 7).

After a suspension of **5** was refluxed in excess CH(OEt)_3_ for 28 h, a 58:42 mixture of the starting material and a new compound was isolated. This reaction completed in 8 h in the presence of HCOOH (2.5 equiv) and the new compound was isolated in 59% yield. According to 1D and 2D NMR, and HRMS (ESI^+^) data, the product obtained was triazolo[1,5-*c*]pyrimidine **13** instead of the expected macrocycle **11**. The structure **13** was unambiguously confirmed by its synthesis directly from pyrazolopyrimidine **8** (ethyl orthoformate, 1.3 equiv of HCOOH, reflux, 15 h).

Analogously, no macrocyclic derivatives (e.g., **12**) were formed from **5** under reflux in AcOH for 7 h. The only isolated product was pyrazolopyrimidine **14** (38%) whose structure was assigned based on NMR and HRMS (ESI^+^) data. It should be noted that all signals in the ^1^H and ^13^C NMR spectra of **14** in DMSO-*d*_6_ except N–CH_3_ and C3–H were extremely broadened. This can be explained by some equilibrium processes including internal rotation of the acetylamino group and/or exchange between C=N bond configurations. As the temperature increases, the width of the signals decreases, however, even at 100 °С, some signals remain broadened.

The failure in the synthesis of compounds **11** and **12** can be associated with difficulties in the annulation of the triazole rings due to the high conformational rigidity of macrocycle **5** (see Supporting Information File 1). Thus, instead of the formation of **11** and **12**, side reactions occur leading to the splitting of the macrocyclic ring in **5**. The relatively low stability and tendency to disintegration of the macrocyclic ring in **5** can be explained by its somewhat anti-aromatic character (see above).

## Conclusion

In conclusion, self-assembly of 14-membered 1,2,4,8,9,11-hexaazamacrocycle annulated with two pyrazole rings, 2,10-dimethyl-2,8,10,16-tetrahydrodipyrazolo[3,4-*e*:3',4'-*l*][1,2,4,8,9,11]hexaazacyclotetradecine-4,12-diamine (**5**), proceeding by the reaction of 3-[(ethoxymethylene)amino]-1-methyl-1*H*-pyrazole-4-carbonitrile (**4**) with excess hydrazine was reinvestigated in detail. Under all tested conditions, including the reported ones, the product yield was significantly lower compared with the reported one and did not exceed 38%. Moreover, in all cases the isolated product was a mixture of the desired macrocycle and a noticeable amount of the admixture of bis-pyrazole structure (>18–19 mol %). We demonstrated that the reaction proceeds through the initial formation of 4-imino-2-methyl-2,4-dihydro-5*H*-pyrazolo[3,4-*d*]pyrimidin-5-amine (**8**) undergoing dimerization to give the final macrocycle. Preparative protocol for the macrocycle synthesis on a multi-gram scale starting from 4-imino-2-methyl-2,4-dihydro-5*H*-pyrazolo[3,4-*d*]pyrimidin-5-amine (**8**) was developed. Under the optimized conditions (1.5 equiv of N_2_H_4_·H_2_O, MeOH, reflux, 3 h), the macrocycle **5** was obtained in a 35% isolated yield after crystallization. We believe that, despite the moderate yield of the macrocycle, the ease of its isolation and purification, the operational simplicity of all the reactions, the high availability of all the reactants make the developed synthesis very promising. A plausible pathway of the hydrazine-promoted self-assembly of the macrocycle from the pyrazolopyrimidine based on the experimental data and the DFT calculations was proposed. It involves nucleophilic attack of hydrazine on the C2 carbon of the pyrimidine ring followed by cleavage of the C2–N3 bond, dimerization of the bis-amidrazone formed, and macrocyclization of the dimer. The DFT calculations also showed that the hydrazine-promoted transformation of the pyrazolopyrimidine **8** into the macrocycle **5** in MeOH is a thermodynamically favorable process with Δ*G* = −5.90 kcal/mol at 298 K and 1 atm. We found that the 14-membered macrocyclic ring can be destroyed under harsh reaction conditions. Thus, under reflux in CH(OEt)_3_ in the presence of HCOOH or in AcOH, the macrocycle afforded pyrazolo[4,3-*e*][1,2,4]triazolo[1,5-*c*]pyrimidine **13** or 5-acetylamino-4-imino-2-methylpyrazolo[3,4-*d*]pyrimidine **14**, respectively. Observed disintegration of the macrocycle **5** can be explained by its relative instability arising from somewhat anti-aromatic character of the macrocyclic core [NICS(0) = +2.23 ppm calculated at the HF/6-31+G(d) level].

Since numerous heterocyclic and carbocyclic analogs of 3-amino-1-methyl-1*H*-pyrazole-4-carbonitrile (**3**), which is the starting material in the synthesis of macrocycle **5**, are readily available, we believe that our results will be useful for the preparation of other polyunsaturated annulated 14-membered 1,2,4,8,9,11-hexaazamacrocycles.

## Experimental

All solvents and liquid reagents purchased from commercial sources were distilled prior to use. Petroleum ether had a distillation range of 40–70 °C. 100% Hydrazine hydrate was used in the syntheses. Anhydrous N_2_H_4_ was obtained from N_2_H_4_·H_2_O according to the standard procedure. All other reagents were purchased from commercial sources and used without additional purification. FTIR spectra were recorded using a Bruker Alpha-T spectrophotometer in KBr. Band characteristics in the IR spectra are defined as very strong (vs), strong (s), medium (m), weak (w), shoulder (sh), and broad (br). NMR spectra (solutions in DMSO-*d*_6_ or CDCl_3_) were acquired using a Bruker Avance III 600 spectrometer at 600.13 (^1^H) and 150.90 (^13^C) MHz. ^1^H NMR chemical shifts are referenced to the residual proton signal in DMSO-*d*_6_ (2.50 ppm) or CDCl_3_ (7.24 ppm). In ^13^C NMR spectra, the central signal of DMSO-*d*_6_ (39.50 ppm) or CDCl_3_ (77.23 ppm) was used as a reference. Multiplicities are reported as singlet (s), doublet (d), triplet (t), quartet (q), and some combinations of these, multiplet (m). Selective ^1^H–^1^H decoupling, DEPT-135 experiments as well as HMQC, HMBC, and NOESY correlation techniques were used to aid in the assignment of ^1^H and ^13^C NMR signals. Elemental analyses (CHN) were performed using a Thermo Finnigan Flash EA1112 apparatus. High-resolution mass spectra (HRMS) were obtained using a Bruker mikrOTOF II focus spectrometer (ESI^+^). All yields refer to isolated and spectroscopically pure material. The color of the solids is white if not otherwise mentioned. The geometry optimizations were carried out at the B3LYP level of theory using Gaussian 16 suite [55] of quantum chemical programs. Pople’s basis sets, 6-311++G(d,p), was employed for geometry optimization. The effect of continuum solvation was incorporated by using the polarizable continuum model (PCM). Enthalpies and Gibbs free energies were obtained by adding unscaled zero-point vibrational energy corrections (ZPVE) and thermal contributions to the energies (temperature 298.150 Kelvin, pressure 1.000 atm). Carbon chemical shifts of the prepared compounds in DMSO were calculated by the GIAO method at the PBE1PBE/6-311+G(2d,p) level of theory using the DFT B3LYP/6-311++G(d,p) optimized geometries (DMSO solution) and applying a multi-standard approach [44].

**3-[(Ethoxymethylene)amino]-1-methyl-1*H*-pyrazole-4-carbonitrile (4):** Imidate **4** was prepared according to the literature method [43]. Our modification of the method is provided below in details. A solution of aminopyrazole **3** [42] (9.044 g, 74.10 mmol) in HC(OEt)_3_ (145 mL) was stirred under reflux for 23 h. After the reaction had completed (monitored by ^1^H NMR spectroscopy), the obtained solution was concentrated under water pump vacuum upon heating in a water bath at 65 °C. The product was extracted from the resulting dense brown oil by trituration 4 times with a mixture of ether (25 mL) and petroleum ether (50 mL), and 1 time with a mixture of ether (10 mL) and petroleum ether (20 mL) at room temperature. The extraction was considered as completed when the brown oil solidified. The product precipitated upon concentration of the combined extracts under reduced pressure. The concentration was carried out until a suspension convenient for filtering was obtained (not to dryness). The suspension was cooled (−18 °C), the precipitate was filtered, washed with cold petroleum ether (3 × 20 mL), and dried to give imidate **4** (10.373 g, 79%) as a very light creamy solid which was used in the next step. ^1^H NMR (600.13 MHz, CDCl_3_) δ 8.19 (tq, ^4^*J* = 0.8, ^5^*J* = 0.5 Hz, 1Н, CH=N), 7.62 (q, ^4^*J* = 0.5 Hz, 1Н, H-5), 4.36 (dq, ^3^*J* = 7.1, ^4^*J* = 0.8 Hz, 2Н, OСH_2_), 3.81 (d, ^4^*J* = 0.5 Hz, 3Н, NCH_3_), 1.35 (dt, ^3^*J* = 7.1, ^5^*J* = 0.5 Hz, 3H, СH_3_ in OEt); ^13^C NMR (150.90 MHz, CDCl_3_) δ 158.97 (CH=N), 157.80 (С-3), 135.72 (С-5), 113.58 (СN), 85.70 (С-4), 63.46 (OСH_2_), 39.85 (NCH_3_), 14.19 (СH_3_ in OEt).

**2,10-Dimethyl-2,8,10,16-tetrahydrodipyrazolo[3,4-*e*:3',4'-*l*][1,2,4,8,9,11]hexaazacyclotetradecine-4,12-diamine (5):** A mixture of pyrazolopyrimidine **8** (4.039 g, 24.60 mmol) and N_2_H_4_·H_2_O (1.859 g, 37.13 mmol) in МеОН (29 mL) was stirred under reflux for 3 h, and cooled in refrigerator (about +6 °C). The precipitate was filtered, washed with cold EtOH (4 × 15 mL), cold ether (2 × 15 mL), and dried to give a mixture of macrocycle **5** and bis-pyrazole **6** in a ratio of 89:11 (1.858 g, very light pink solid). This mixture (1.026 g) was recrystallized from boiling DMF (255 mL). After precipitation completed, the solid was filtered, washed with cold DMF (3 × 5 mL). The filter cake was suspended in water (10 mL) for 5 min followed by suction, this procedure was repeated 3 times to remove DMF from crystals. After drying in a vacuum desiccator over P_2_O_5_ pure macrocycle **5** (0.779 g, 35%) was obtained as scarcely pink solid (the initial color was not completely removed by crystallization). Mp 298 °C dec (colorless foam, DMF; note: the solid had a very sharp melting point that varies in the range of 290‒299 °C depending on the heating rate) [lit [40] mp 269–270 °C (MeOH)]; IR (KBr, cm^−1^) ν: 3423 (br s), 3365 (s), 3307 (br s), 3182 (br s), 1626 (sh), 1596 (vs), 1555 (s), 1547 (sh), 1242 (s), 1164 (s), 965 (s); ^1^H NMR (600.13 MHz, DMSO-*d*_6_) δ 11.82 (d, ^3^*J* = 11.2 Hz, 2Н, two NH), 8.01 (q, ^4^*J* = 0.5 Hz, 2Н, H-3 and H-11), 7.47 (d, ^3^*J* = 11.2 Hz, 2Н, H-7 and H-15), 6.62 (br s, 4Н, two NH_2_), 3.73 (d, ^4^*J* = 0.5 Hz, 6Н, two CH_3_); ^13^C NMR (150.90 MHz, DMSO-*d*_6_) δ 152.25 (С-4 and C-12), 147.62 (С-8a and C-16a), 138.19 (С-7 and C-15), 130.65 (С-3 and C-11), 100.48 (С-3a and C-11a), 38.80 (two СH_3_); HRESIMS–TOF (*m*/*z*): [M + H]^+^ calcd for C_12_H_17_N_12_, 329.1694; found, 329.1691; Anal. calcd for C_12_H_16_N_12_·0.85H_2_O: C, 41.94; H, 5.19; N, 48.91; found: C, 41.67; H, 4.80; N, 48.83.

**4-Imino-2-methyl-2,4-dihydro-5*H*-pyrazolo[3,4-*d*]pyrimidin-5-amine (8):** To a cooled (ice bath), stirred solution of compound **4** (5.836 g, 32.77 mmol) in EtOH (70 mL) was added a solution of N_2_H_4_·H_2_O (2.470 g, 49.33 mmol) in EtOH (10 mL) over 3 min. The reaction mixture was stirred at room temperature for 1 h. The resulting suspension was cooled (−18 °C), the precipitate was filtered, washed with cold EtOH (4 × 15 mL), and dried to give compound **8** (5.152 g, 96%) as a white solid which was used in the next step. IR (KBr, cm^–1^) ν: 3294 (m), 3251 (s), 3157 (m), 3130 (s), 1662 (vs), 1583 (s), 1552 (m), 1228 (s), 1155 (m), 961 (s), 853 (s), 764 (s); ^1^H NMR (600.13 MHz, DMSO-*d*_6_) δ 8.25 (unresolved q, 1Н, H-3), 7.87 (s, 1Н, H-6), 7.61 (very br s, 1H, C=NH), 5.40 (br s, 2Н, N-NH_2_), 3.91 (d, ^4^*J* = 0.5 Hz, 3Н, CH_3_); ^13^C NMR (150.90 MHz, DMSO-*d*_6_) δ 155.02 (С-7a), 151.77 very br (С-4), 149.89 (С-6), 128.37 (С-3), 105.45 (С-3a), 39.54 (СH_3_); Anal. calcd for C_6_H_8_N_6_·0.5H_2_O: C, 41.61; H, 5.24; N, 48.53; found: C, 41.55; H, 4.88; N, 48.87.

**3-(3-Amino-1-methyl-1*H*-pyrazol-4-yl)-4*H*-1,2,4-triazol-4-amine (10):** To an emulsion of N_2_H_4_·H_2_O (0.301 g, 6.01 mmol) in 1,4-dioxane (2 mL) was added TsOH·H_2_O (0.019 g, 0.10 mmol) and the mixture was stirred until solids completely dissolved. Then, pyrazolopyrimidine **8** (0.337 g, 2.05 mmol) and 1,4-dioxane (2 mL) were added, the reaction mixture was stirred under reflux for 1 h, and the volatiles were removed under vacuum. The solid residue was triturated with saturated aqueous NaHCO_3_ until a suspension formed, and cooled to 0 °C. The precipitate was filtered, washed with ice-cold water, petroleum ether, ether, and dried to give a mixture of compounds **5**, **6**, and **10** in a ratio of 13:0.5:86.5 (0.294 g) as a violet powder. This mixture (0.267 g) was completely dissolved in boiling DMF (15.6 mL), the solution was filtered through a rough paper filter and left in the refrigerator (about +6 °C) until crystallization completed. The precipitated solid was filtered and washed with cold DMF (3 × 2 mL). The ^1^H NMR spectrum showed that the solid was pure macrocycle **5**. The mother liquor was collected, H_2_O (20 mL) was added and the solution was left standing overnight at room temperature. After that, some additional violet crystals precipitated and were collected by filtration, the mother liquor was concentrated under vacuum. To the obtained dense oily residue was added H_2_O (35 mL), the resulting light pink solution was cooled (0 °C), and upon manual agitation the precipitation of product was initiated. After precipitation completed, the solid was filtered, washed with ice-cold H_2_O, and dried to give compound **10** (0.104 g, 28%) as a light pink solid. Mp 204.5–206 °C (water); IR (KBr, cm^–1^) ν: 3414 (br vs), 3315 (br vs), 3200 (br s), 3143 (s), 3094 (s), 1721 (w), 1647 (w), 1605 (vs), 1538 (s), 1517 (s), 1407 (s), 1216 (m), 1131 (s), 962 (s), 860 (s), 738 (s); ^1^H NMR (600.13 MHz, DMSO-*d*_6_) δ 8.28 (s, 1Н, H-5 in triazole ring), 8.12 (q, ^4^*J* = 0.5 Hz, 1Н, H-5 in pyrazole ring), 6.15 (s, 2Н, NH_2_ in triazole ring), 5.46 (br s, 2H, NH_2_ in pyrazole ring), 3.67 (d, ^4^*J* = 0.5 Hz, 3Н, CH_3_); ^13^C NMR (150.90 MHz, DMSO-*d*_6_) δ 154.15 (С-3 in pyrazole ring), 148.78 (С-3 in triazole ring), 143.95 (С-5 in triazole ring), 129.10 (С-5 in pyrazole ring), 92.64 (С-4 in pyrazole ring), 38.18 (СH_3_); HRESIMS–TOF (*m*/*z*): [M + Na]^+^ calcd for C_6_H_9_N_7_Na, 202.0812; found, 202.0817; Anal. calcd for C_6_H_9_N_7_·0.88H_2_O: C, 36.95; H, 5.56; N, 50.27; found: C, 36.64; H, 5.16; N, 49.99.

**8-Methyl-8*H*-pyrazolo[4,3-*e*][1,2,4]triazolo[1,5-*c*]pyrimidine (13):** Method A: A mixture of pyrazolopyrimidine **8** (0.053 g, 0.03 mmol), CH(OEt)_3_ (4 mL) and НСООН (0.016 mL) was stirred under reflux for 15 h, then the volatiles were removed under vacuum to dryness. The residue was triturated with H_2_O and one drop of saturated aqueous NaHCO_3_ until a suspension formed, and cooled (0 °C). The precipitate was filtered, washed with ice-cold H_2_O, petroleum ether, and dried to give compound **13** (0.035 g, 61%) as a light yellow solid. An analytically pure sample (a light yellow solid) was obtained after crystallization from EtOH. Mp 296–297 °C (EtOH); IR (KBr, cm^−1^) ν: 3108 (w), 3089 (s), 3050 (m), 1871 (w), 1840 (w), 1662 (s), 1548 (w), 1522 (s), 1327 (m), 1173 (s), 870 (m), 773 (m), 652 (m); ^1^H NMR (600.13 MHz, DMSO-*d*_6_) δ 9.42 (s, 1Н, H-5), 8.86 (q, ^4^*J* = 0.6 Hz, 1Н, H-9), 8.53 (s, 1Н, H-2), 4.19 (d, ^4^*J* = 0.6 Hz, 3Н, CH_3_); ^13^C NMR (150.90 MHz, DMSO-*d*_6_) δ 153.94 (С-2), 153.60 (С-6a), 147.48 (С-9b), 139.93 (С-5), 126.09 (С-9), 101.76 (С-9a), 40.39 (СH_3_); HRESIMS–TOF (*m*/*z*): [M + H]^+^ calcd for C_7_H_7_N_6_, 175.0727; found, 175.0733; Anal. calcd for C_7_H_6_N_6_: C, 48.27; H, 3.47; N, 48.25; found: C, 48.35; H, 3.52; N, 48.18.

Method B: Compound **13** (0.034 g, 59%) as a light yellow solid was prepared from macrocycle **5** (0.055 g, 0.17 mmol), СН(ОЕt)_3_ (4 mL) and НСООН (0.016 mL) (reflux, 8 h) as described in Method A.

**5-Acetylamino-4-imino-2-methylpyrazolo[3,4-*d*]pyrimidine (14):** A suspension of macrocycle **5** (0.304 g, 0.93 mmol) in AcOH (15 mL) was stirred under reflux for 7 h, and after about 6 h from the beginning of the reaction, a solution formed. Then, the solvent was removed under vacuum to dryness. The residue was triturated with a minimal volume of saturated aqueous NaHCO_3_ (≈1 mL), and the resulting suspension was cooled to 0 °C. The precipitate was rapidly filtered on cold filter, rapidly washed 2 times with a minimal volume of ice-cold H_2_O (2 × ≈1 mL), and dried to give compound **14** (0.145 g, 38%) as a white solid. An analytically pure sample (a white solid) was obtained after crystallization from EtOH. Mp 285.5–286.5 °C dec (EtOH); IR (KBr, cm^−1^) ν: 3313 (s), 3113 (br s), 1705 (s), 1624 (s), 1573 (s), 1521 (m), 1304 (m), 1249 (s), 1155 (m), 872 (s), 761 (m); ^1^H NMR (600.13 MHz, DMSO-*d*_6_, 30 °C) δ 9.68 (br s, 1Н, NHC=O), ≈9.50 (very br s, 1Н, C=NH, signal partly overlaps with the signal of the NHC=O proton), 8.24 (br s, 1Н, H-6), 8.06 (s, 1Н, H-3), 3.82 (s, 3Н, NCH_3_), 2.03 (br s, 3Н, CH_3_ in Ac); ^1^H NMR (600.13 MHz, DMSO-*d*_6_, 85 °C) δ 8.19 (s, 1Н, H-6), 8.02 (s, 1Н, H-3), 3.83 (s, 3Н, NCH_3_), 2.10 (s, 3Н, CH_3_ in Ac), signals of the NH protons are not observed; ^13^C NMR (150.90 MHz, DMSO-*d*_6_, 85 °C) δ 168.22 (C=O), 151.71 (С-7a), 146.22 (С-6), 143.51 (С-4), 129.63 (С-3), 103.54 (С-3a), 38.35 (NСH_3_), 22.65 (CH_3_ in Ac); HRESIMS–TOF ( *m*/*z*): [M + Na]^+^ calcd for C_8_H_10_N_6_ONa, 229.0808; found, 229.0815; Anal. calcd for C_8_H_10_N_6_O: C, 46.60; H, 4.89; N, 40.76; found, C, 46.65; H, 4.85; N, 40.86.

## Supporting Information

File 1 Copies of IR, ^1^H and ^13^C NMR spectra of synthesized compounds and computational details.
