# Supplementary material for: Unprecedented synthesis of a 14-membered hexaazamacrocycle
Source: Beilstein J Org Chem. 2023 Nov 15;19:1728–40. doi: 10.3762/bjoc.19.126 (PMC10667714; doi:10.3762/bjoc.19.126)
Supplement: File 1 — Copies of IR, 1H and 13C NMR spectra of synthesized compounds and computational details. [file Beilstein_J_Org_Chem-19-1728-s001.pdf]

BEILSTEIN JOURNAL OF ORGANIC CHEMISTRY

Supporting Information

for

Unprecedented synthesis of a 14-membered hexaazamacrocycle

Anastasia A. Fesenko and Anatoly D. Shutalev

Beilstein J. Org. Chem. **2023,** *19,* 1728–1740. doi:10.3762/bjoc.19.126

Copies of IR, ^{1}H and ^{13}C NMR spectra of synthesized compounds and computational details

License and Terms: This is a supporting information file under the terms of the Creative Commons Attribution License (https://creativecommons.org/licenses/by/4.0). Please note that the reuse, redistribution and reproduction in particular requires that the author(s) and source are credited and that individual graphics may be subject to special legal provisions.
The license is subject to the *Beilstein Journal of Organic Chemistry* terms and conditions: (https://www.beilstein-journals.org/bjoc/terms)

Table of contents

1. Copies of selected ^{1}H NMR spectra of crude products obtained by reaction of **4** or **8** with $N_2H_4 \cdot H_2O$ S2–S5
2. Copies of 1D and 2D NMR spectra of macrocycle **5** S6–S9
3. Copies of 1D and 2D NMR spectra of pyrazolopyrimidine **8** S10–S14
4. Copies of 1D and 2D NMR spectra of pyrazolyl-1,2,4-triazole **10** S15–S19
5. Copies of ^{1}H and ^{13}C spectra of pyrazolo[4,3-*e*][1,2,4]triazolo[1,5-*c*]pyrimidine **13** S20–S21
6. Copies of 1D and 2D NMR spectra of pyrazolopyrimidine **14** S22–S28
7. Copies of IR spectra of compounds **5**, **8**, **10**, **13**, and **14** S29–S33
8. Computational details S34–S74
9. References S75

^{1}H NMR spectrum of the crude product prepared by the reaction of imidate **4** with 4.14 equiv of $N_2H_4 \cdot H_2O$ (EtOH, reflux, 2 h)

(600.13 MHz, DMSO-d_6)

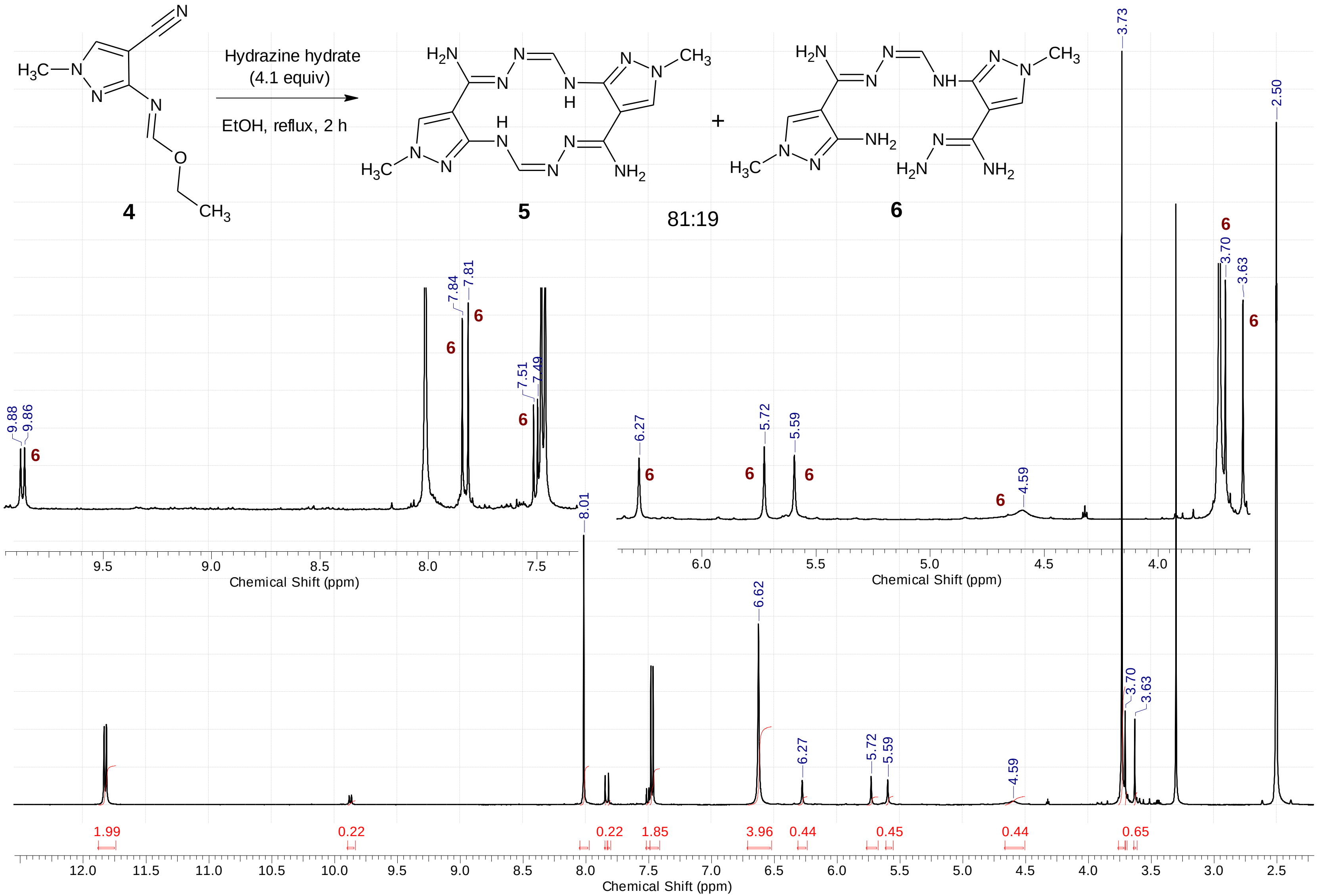

^{1}H NMR spectrum of the crude product prepared by the reaction of **8** with 1.51 equiv of $N_2H_4{\cdot}H_2O$ (MeOH, reflux, 3 h) (600.13 MHz, DMSO-d_6)

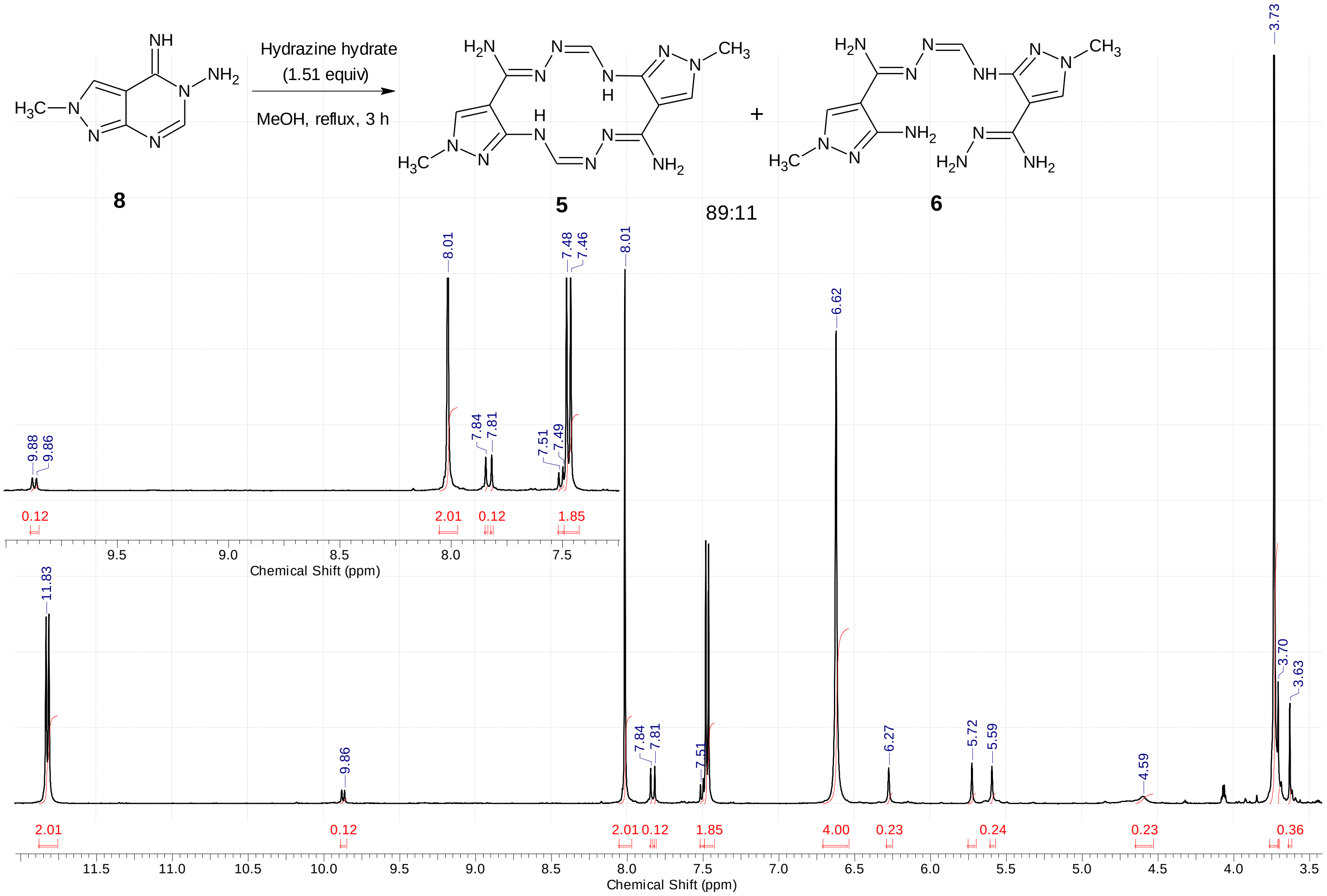

^{1}H NMR spectrum of the crude product prepared by the reaction of **8** with 6.29 equiv of $N_2H_4{\cdot}H_2O$ (EtOH, reflux, 0.5 h)

(600.13 MHz, DMSO-d_6)

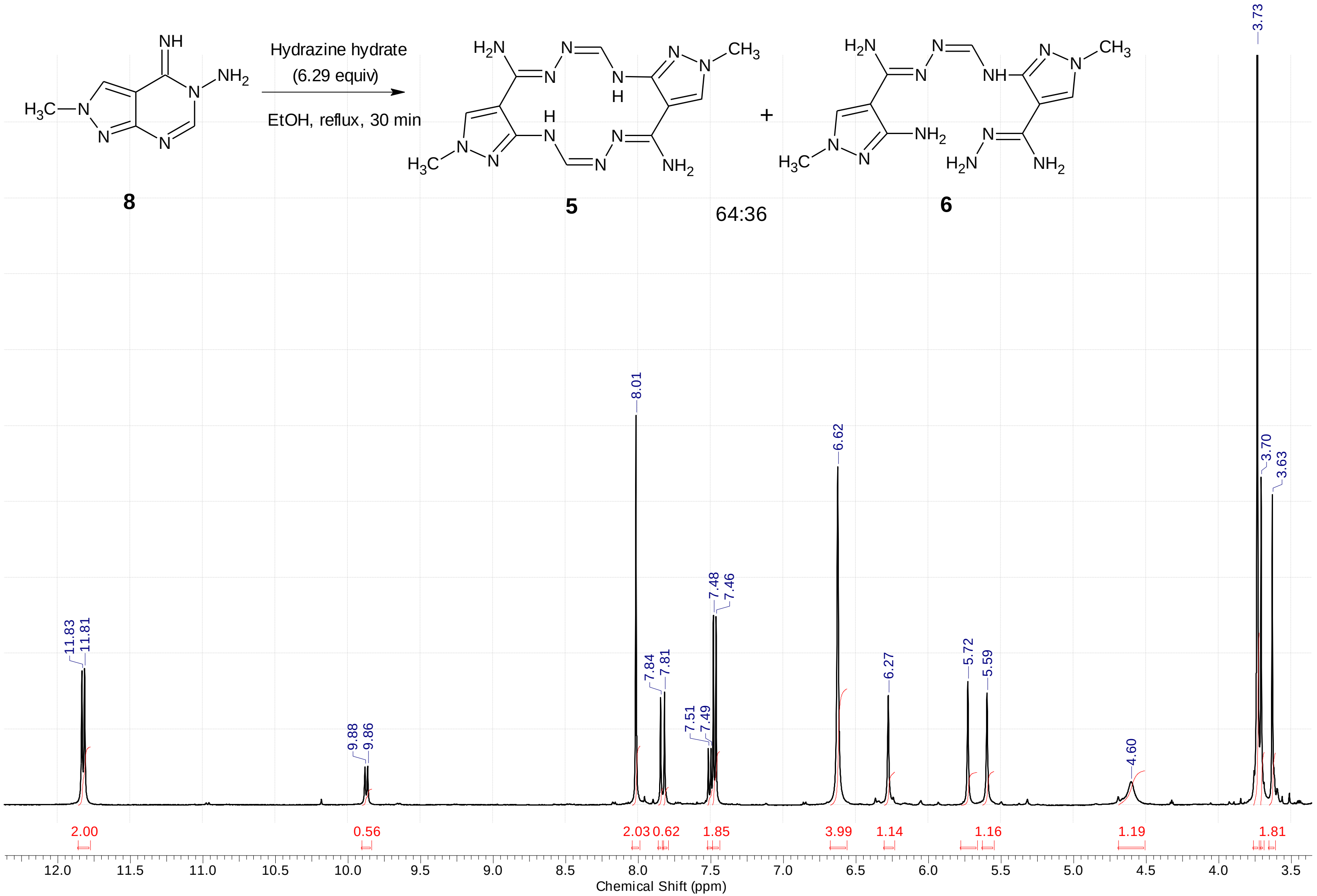

^{1}H NMR spectrum of the crude product prepared by the reaction of **8** with $N_2H_4 \cdot H_2O$ (2.93 equiv) and $TsOH \cdot H_2O$ (0.05 equiv) (dioxane, reflux, 1 h) (600.13 MHz, DMSO-d_6)

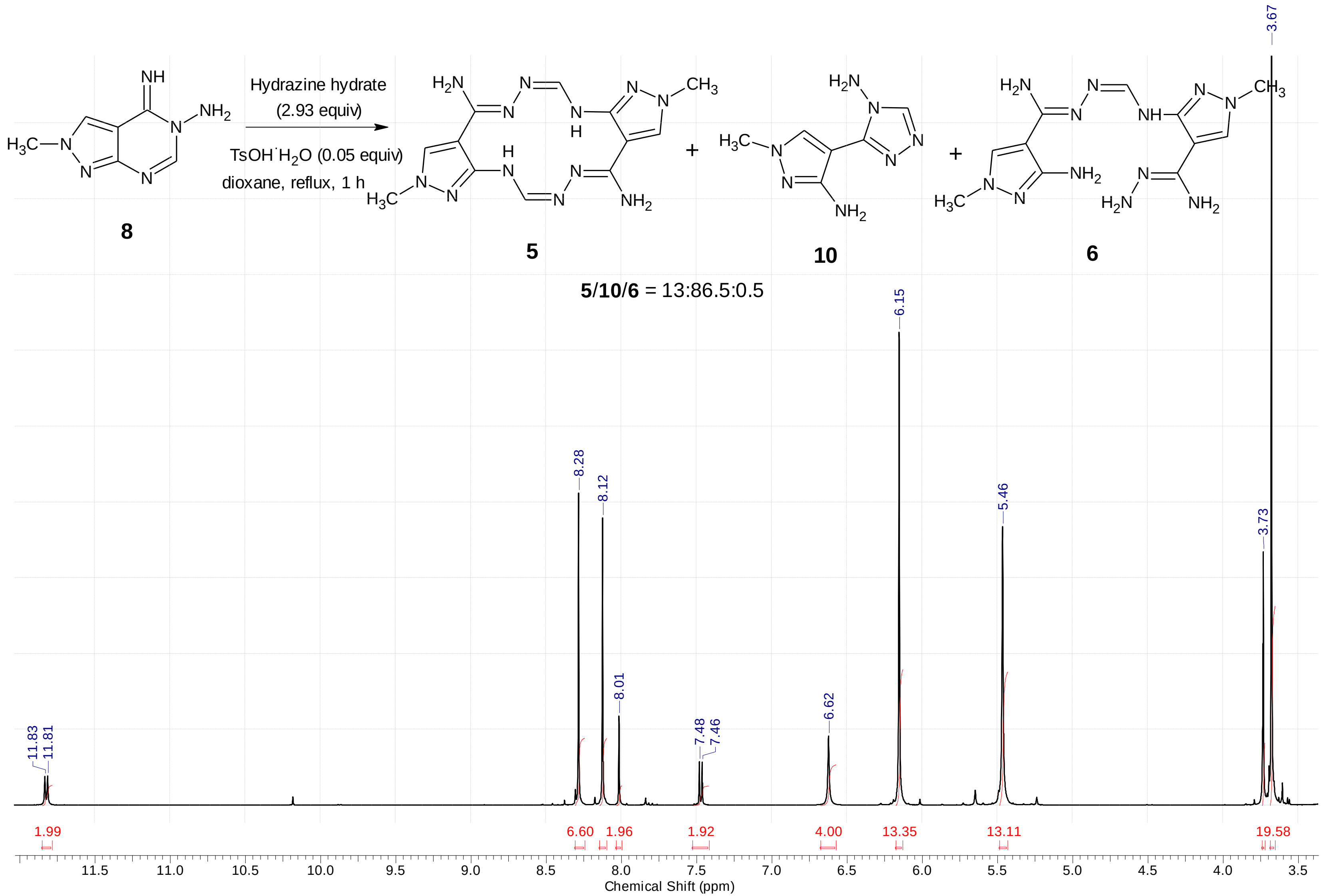

^{1}H NMR spectrum of compound **5** (600.13 MHz, 30 °C, DMSO-d_6).

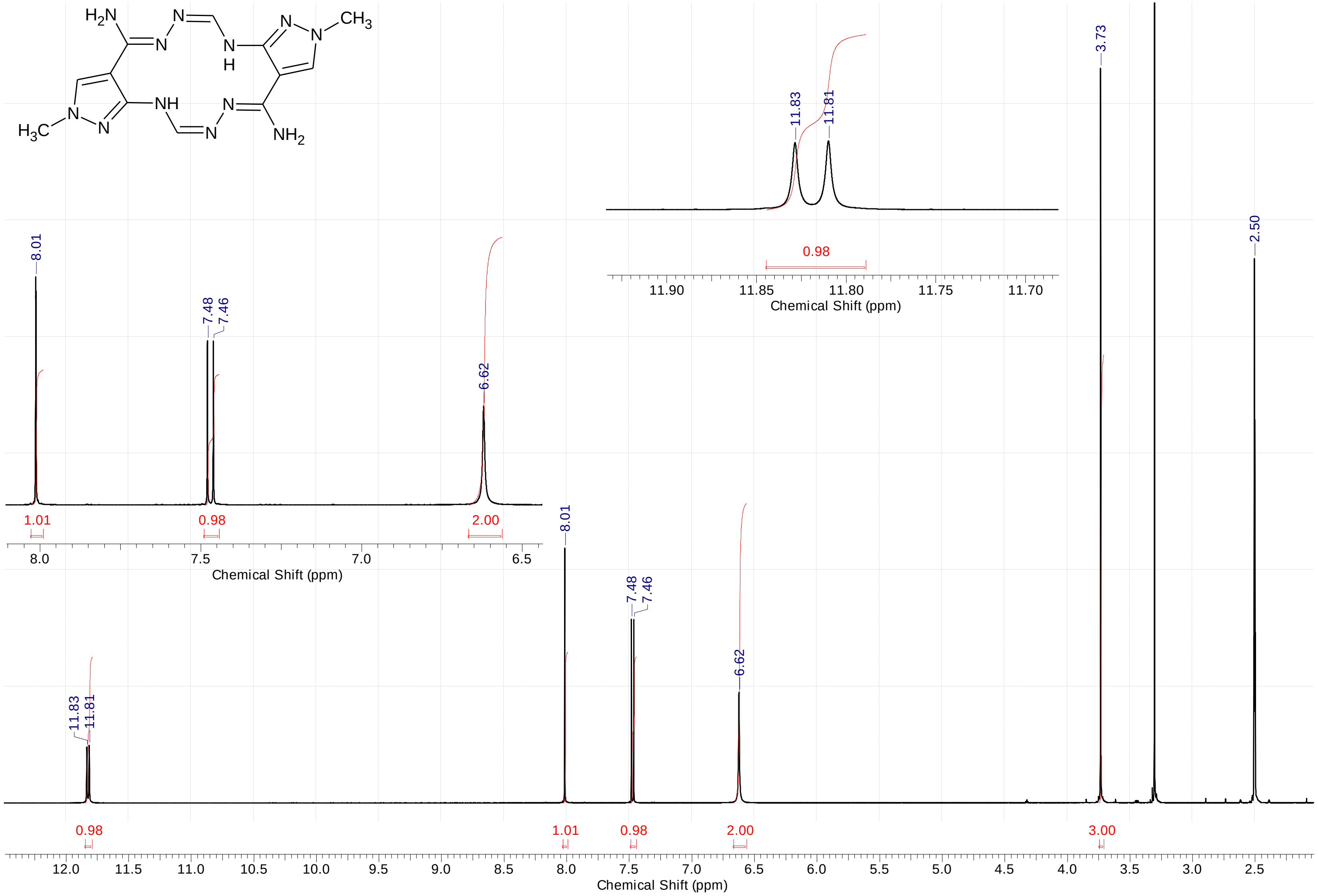

^{13}C NMR spectrum of compound **5** (150.90 MHz, 30 °C, DMSO-d_6)

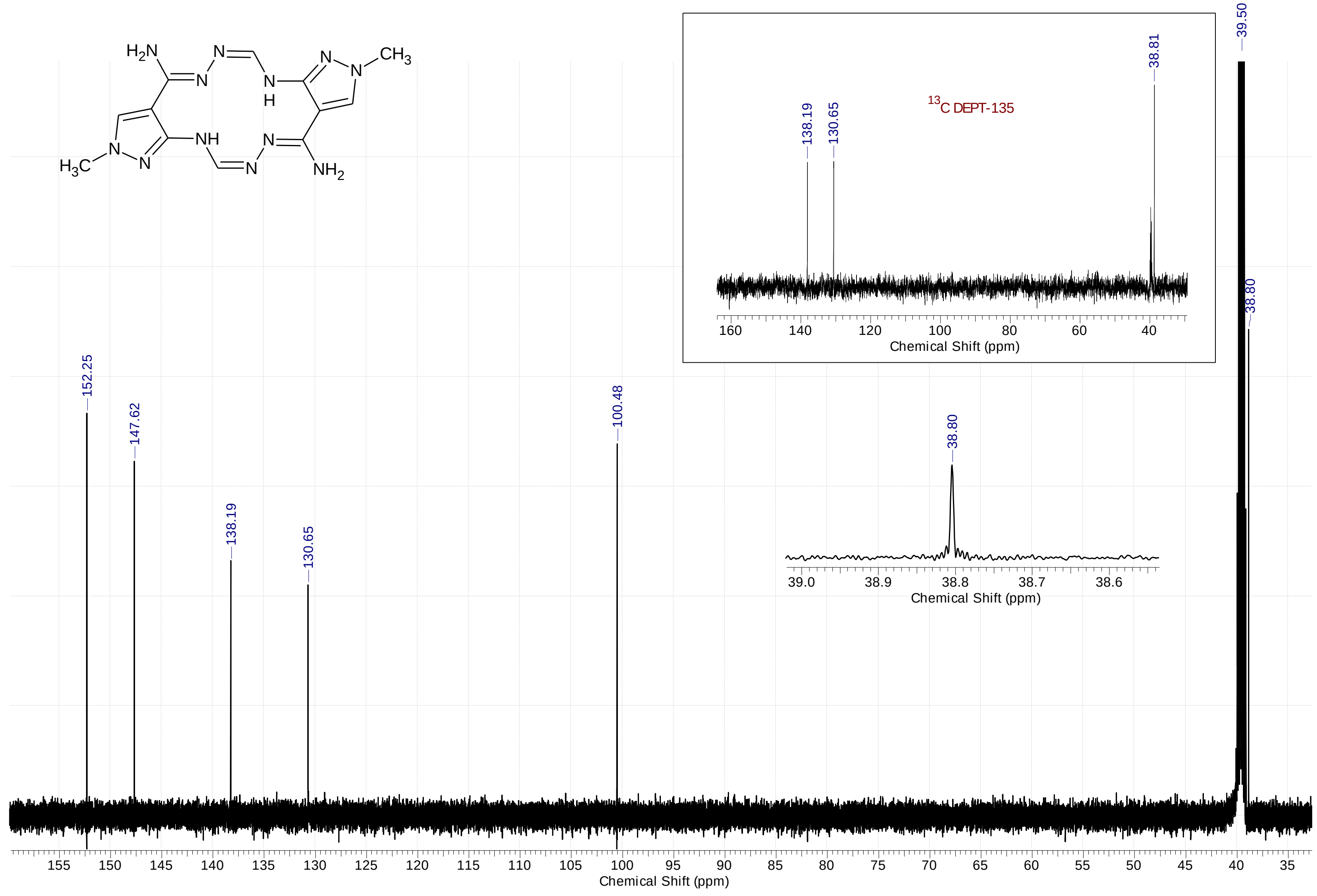

^{1}H,^{13}C HSQC spectrum of compound **5** (Bruker Avance III, 30 °C, DMSO-d_6)

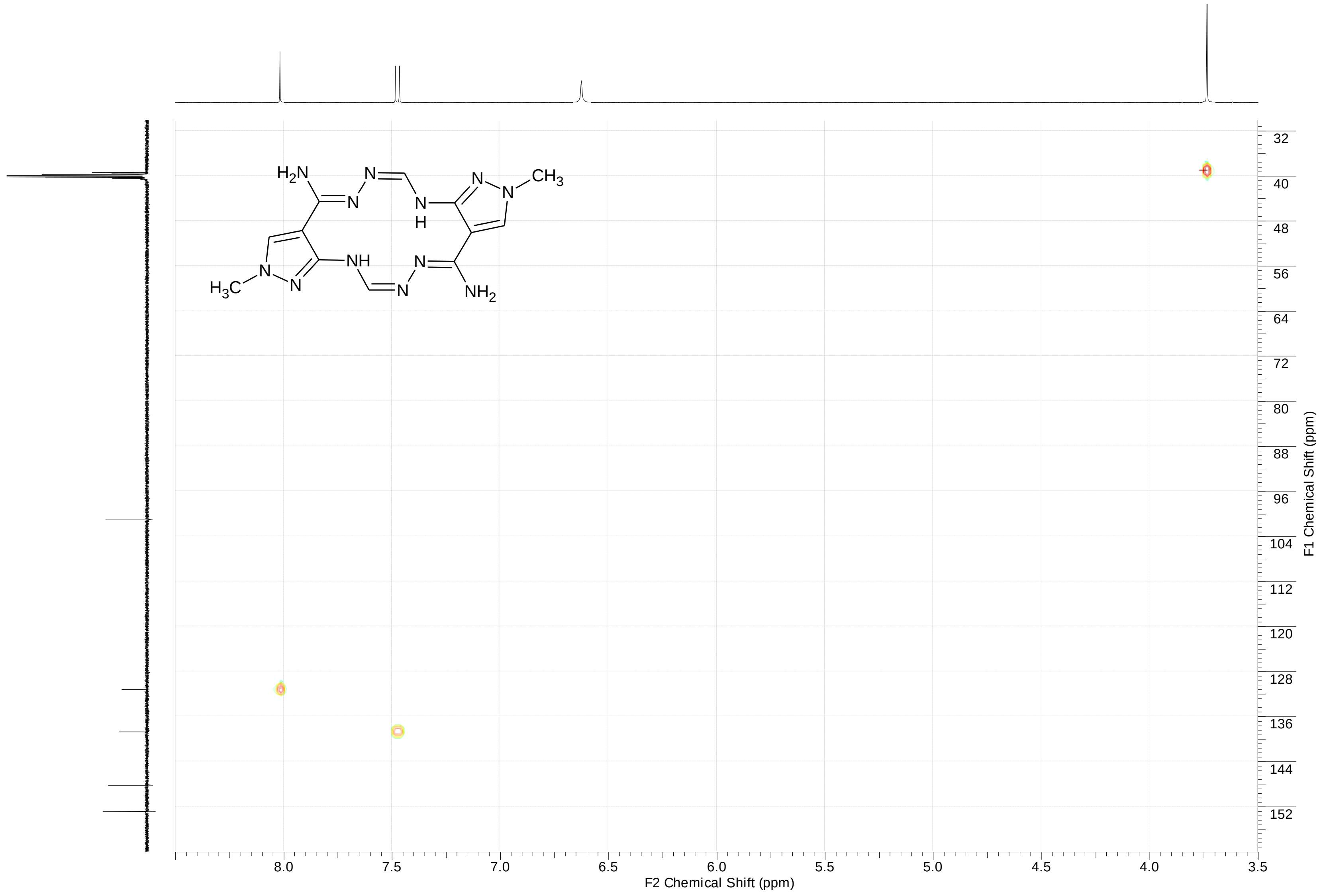

^{1}H,^{13}C HMBC spectrum of compound **5** (Bruker Avance III, 30 °C, DMSO-d_6)

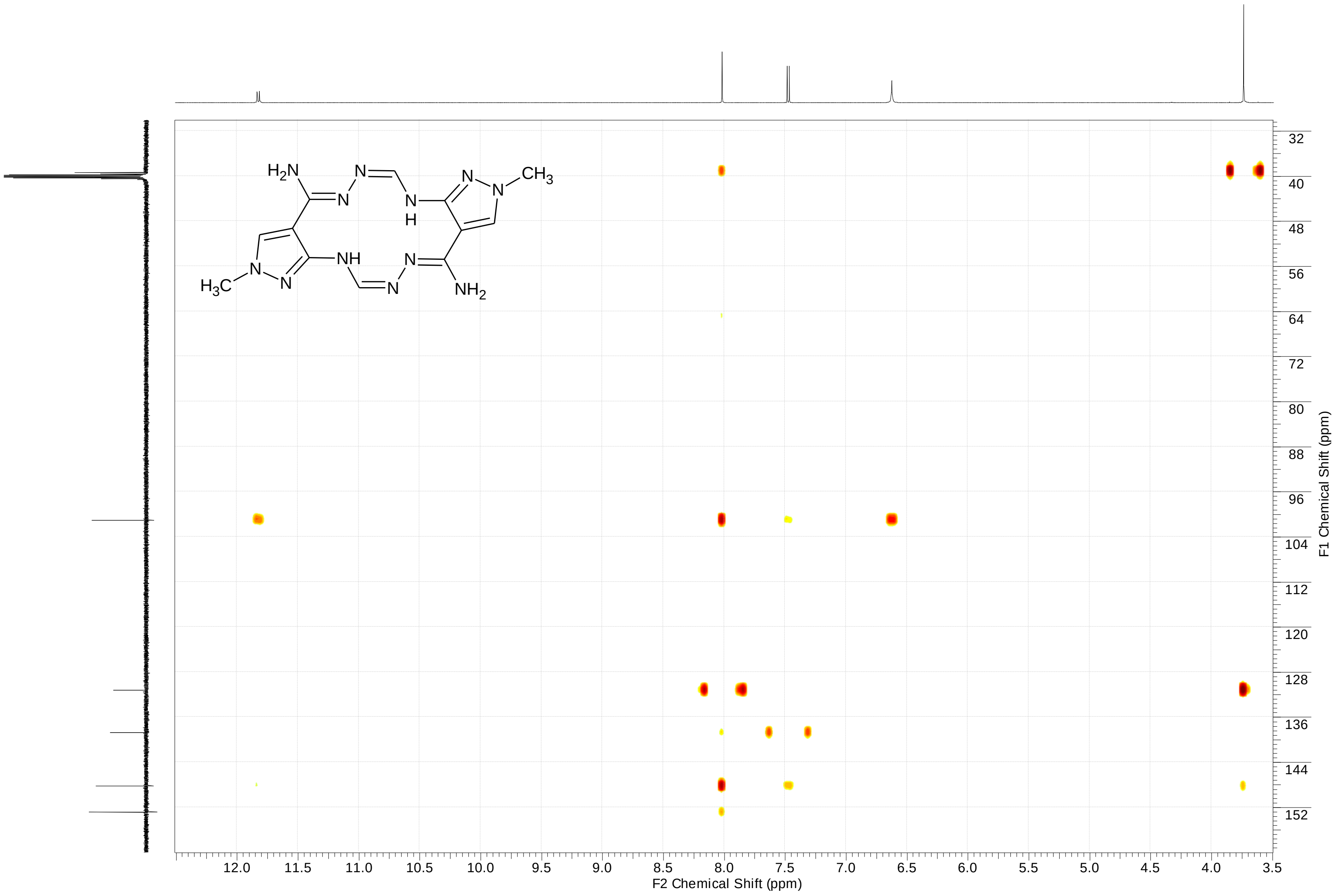

^{1}H NMR spectrum of compound **8** (600.13 MHz, 30 °C, DMSO-d_6).

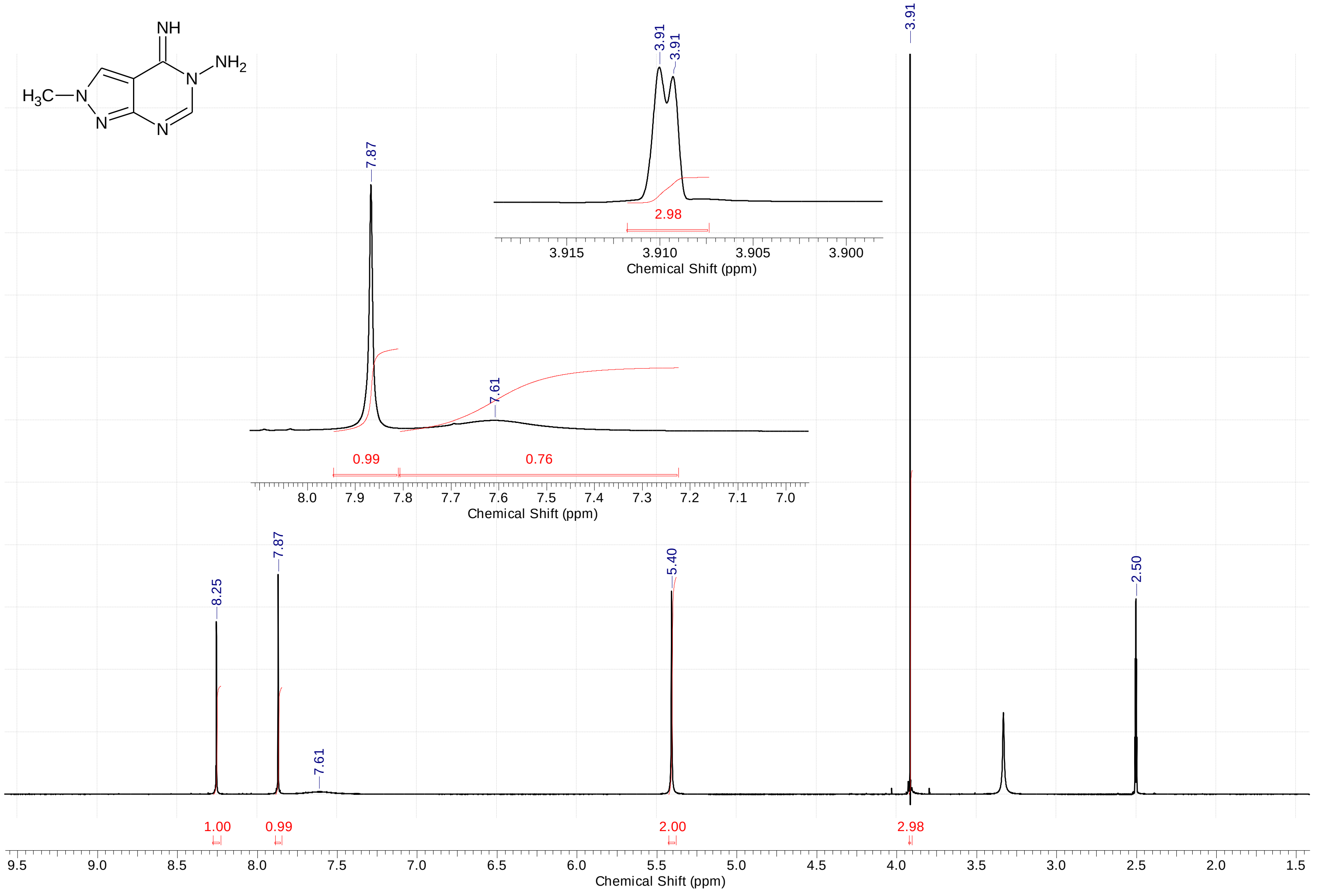

^{13}C NMR spectrum of compound **8** (150.90 MHz, 30 °C, DMSO-d_6)

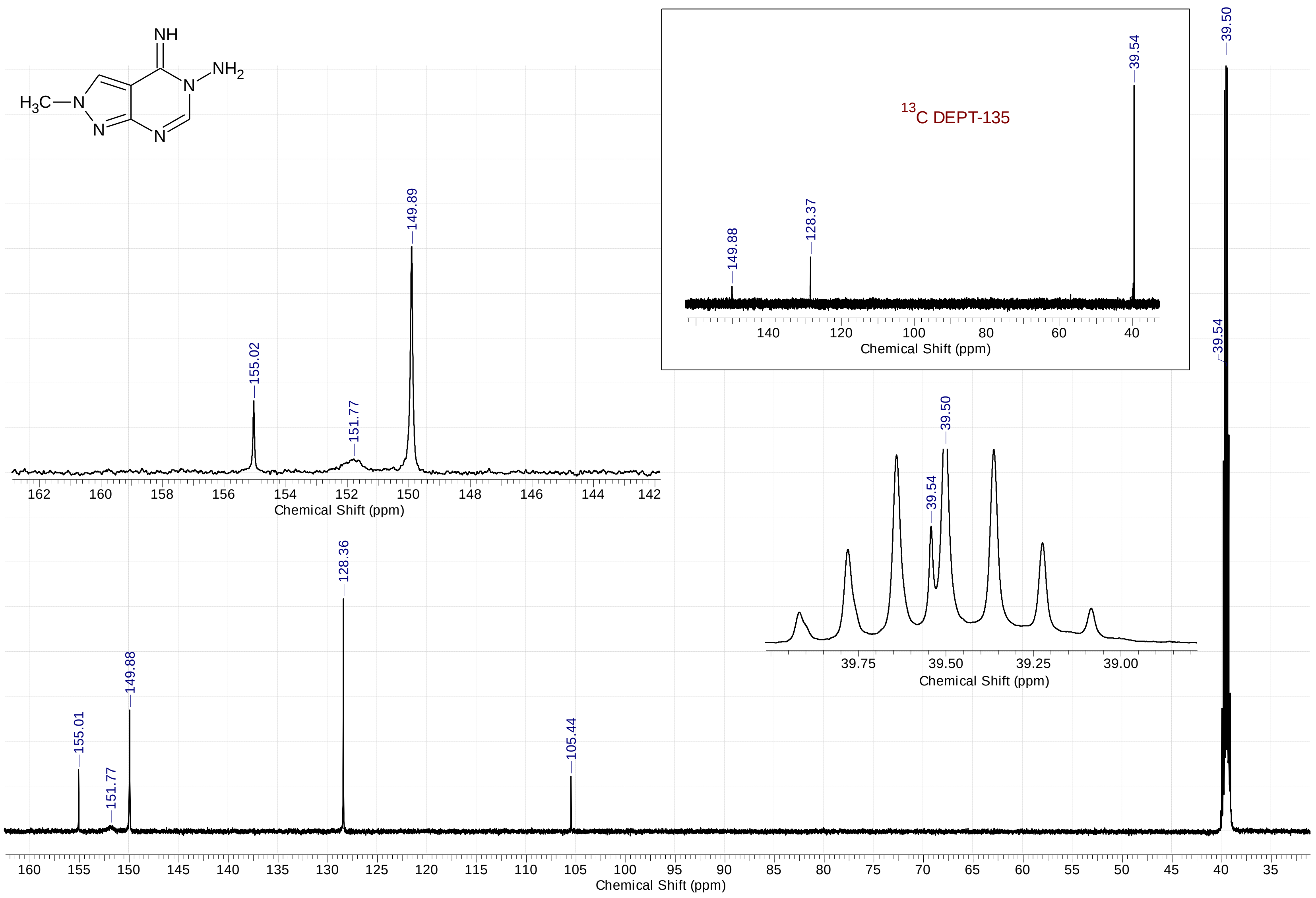

$^{1}H,^{13}C$ HSQC spectrum of compound **8** (Bruker Avance III, 30 °C, DMSO-d_6)

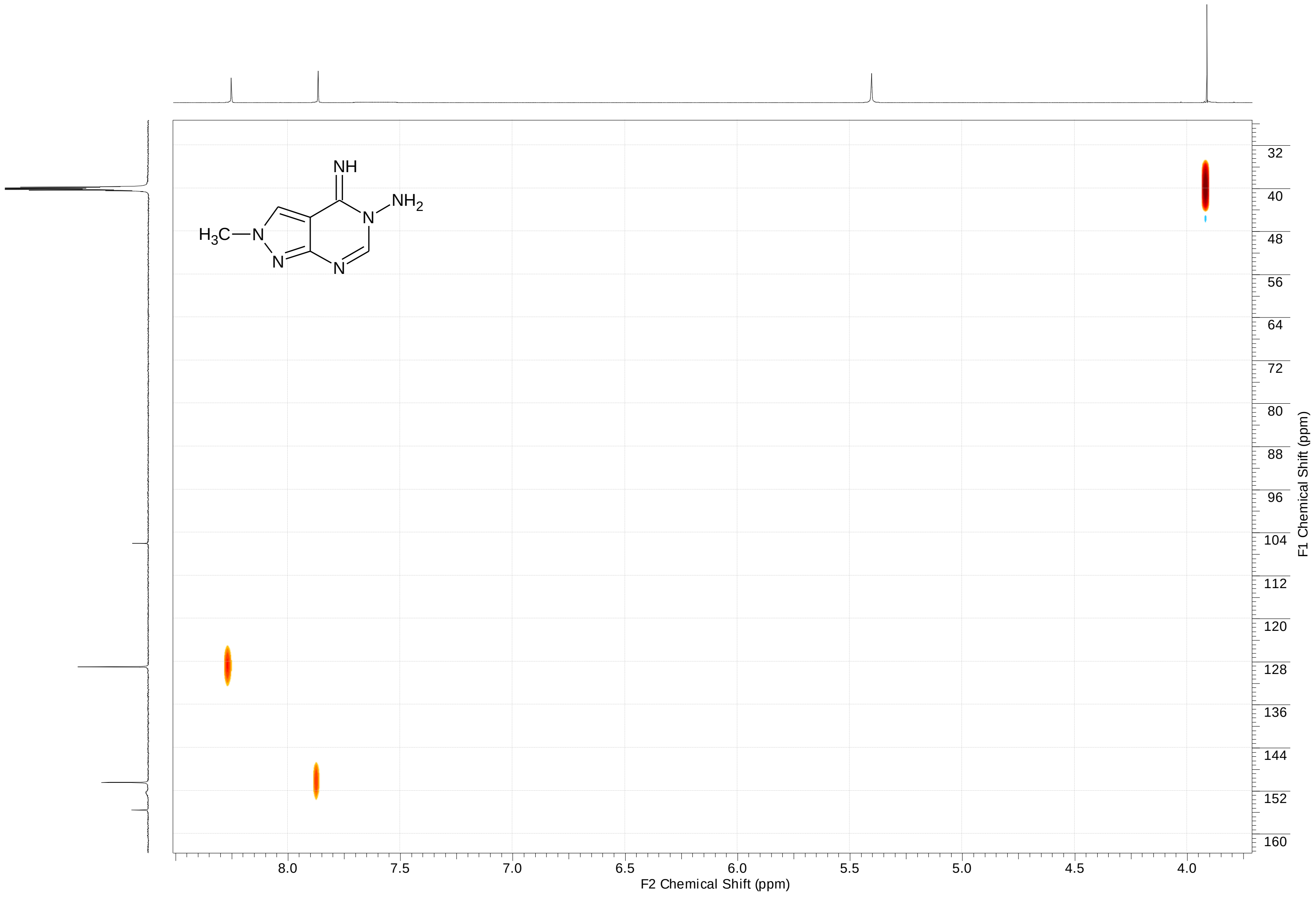

$^{1}H,^{13}C$ HMBC spectrum of compound **8** (Bruker Avance III, 30 °C, DMSO-d_6)

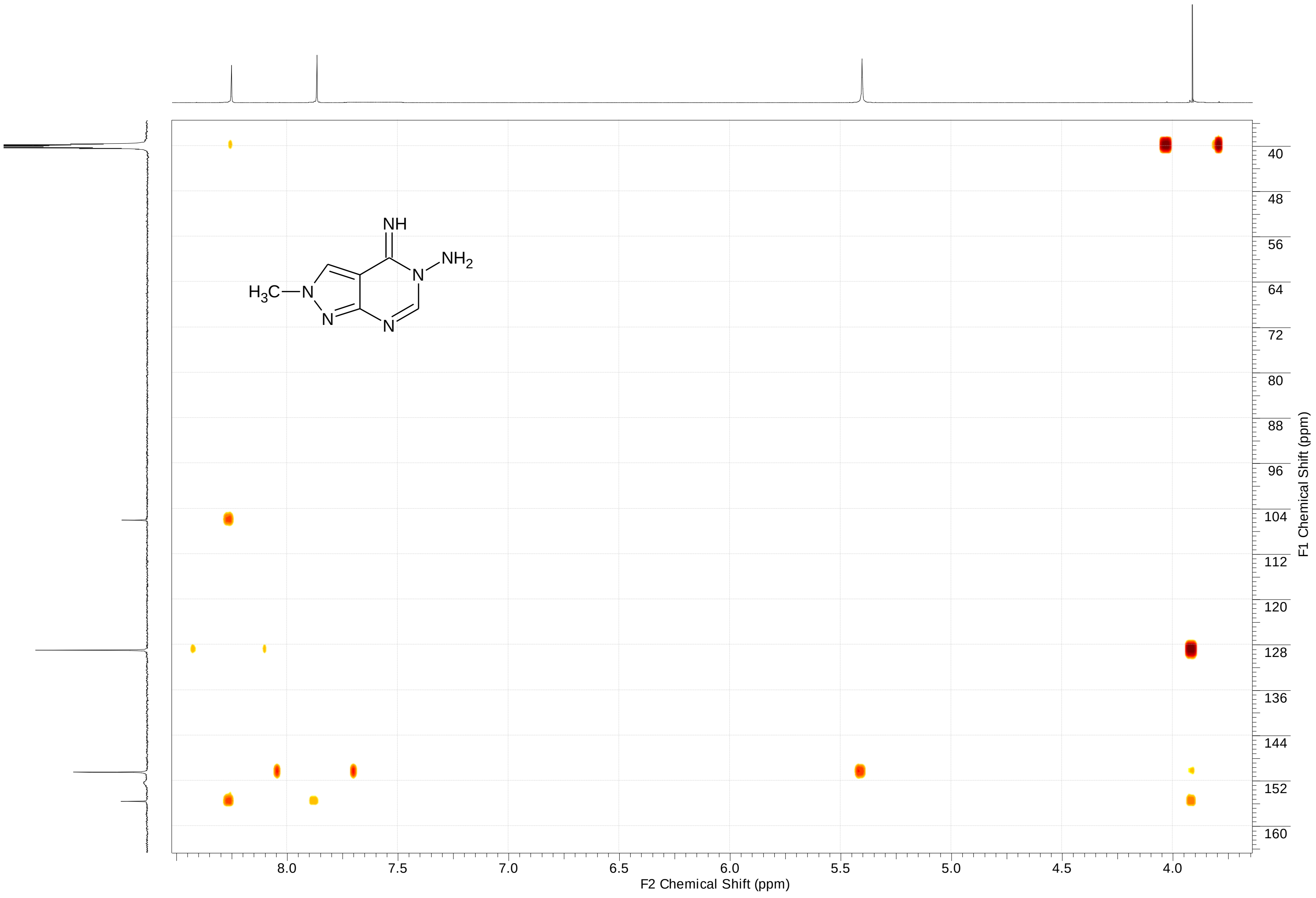

^{1}H,^{1}H NOESY spectrum of compound **8** (600.13 MHz, 30 °C, DMSO-d_6)

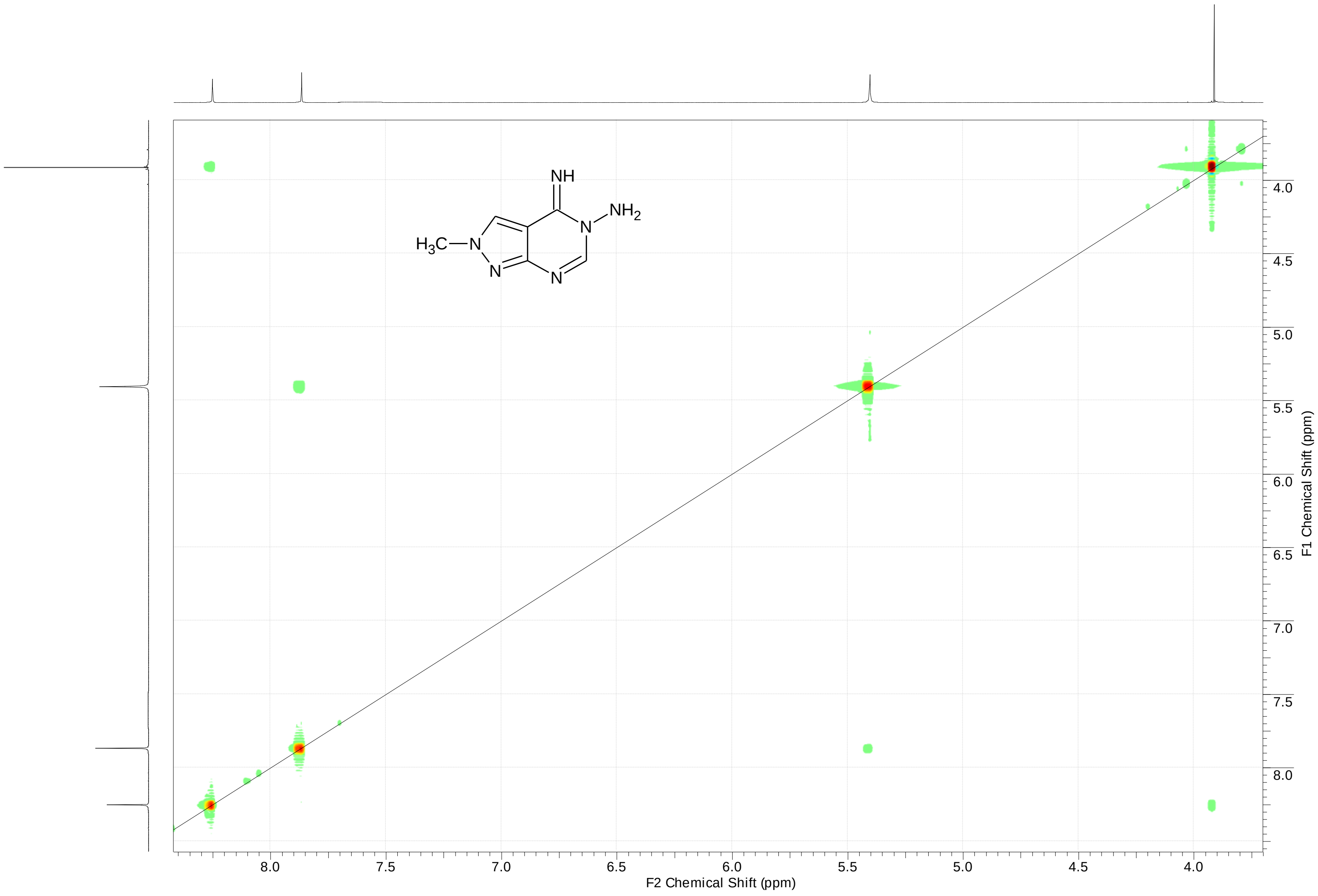

^{1}H NMR spectrum of compound **10** (600.13 MHz, 30 °C, DMSO-d_6).

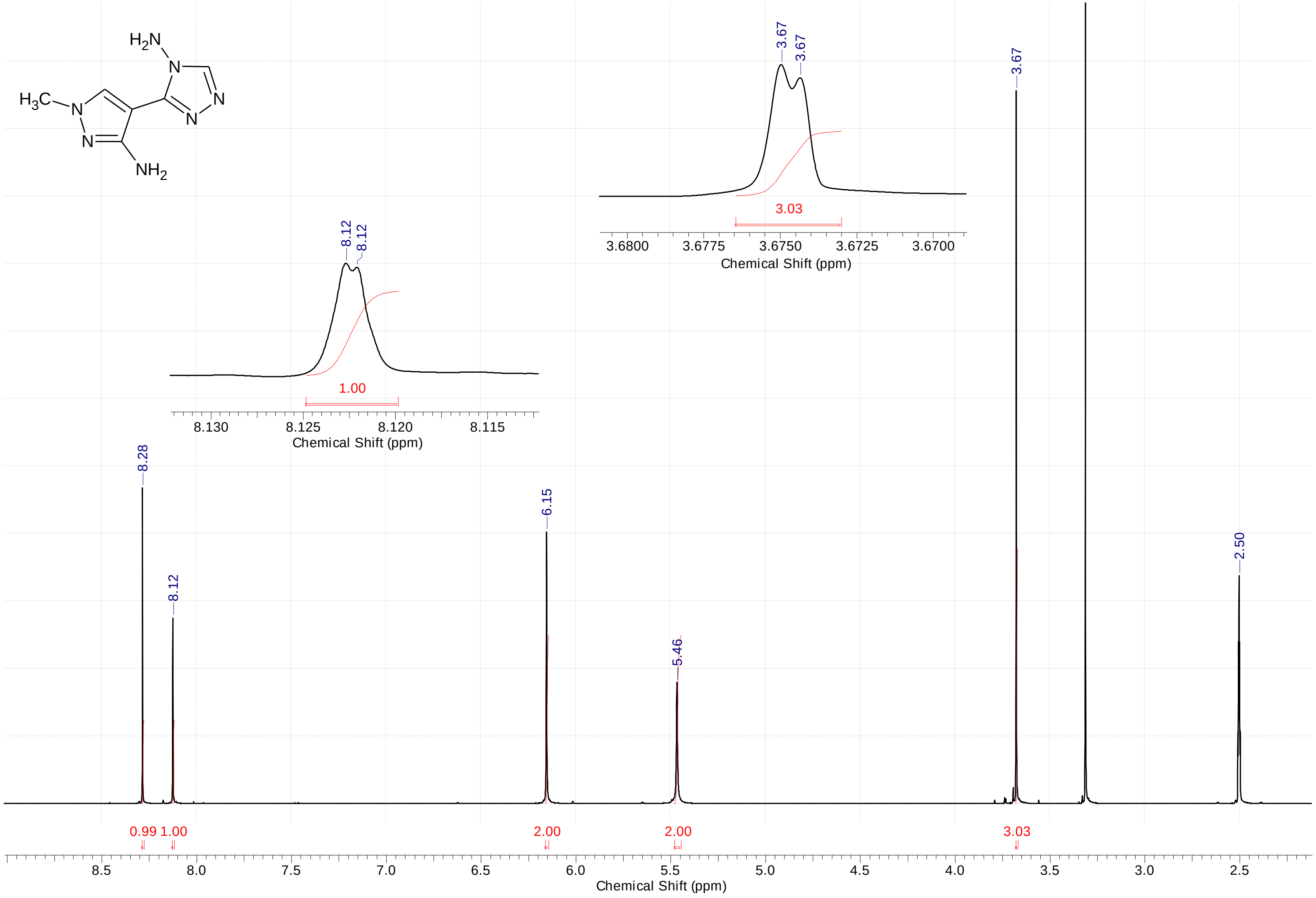

^{13}C NMR spectrum of compound **10** (150.90 MHz, 30 °C, DMSO-d_6)

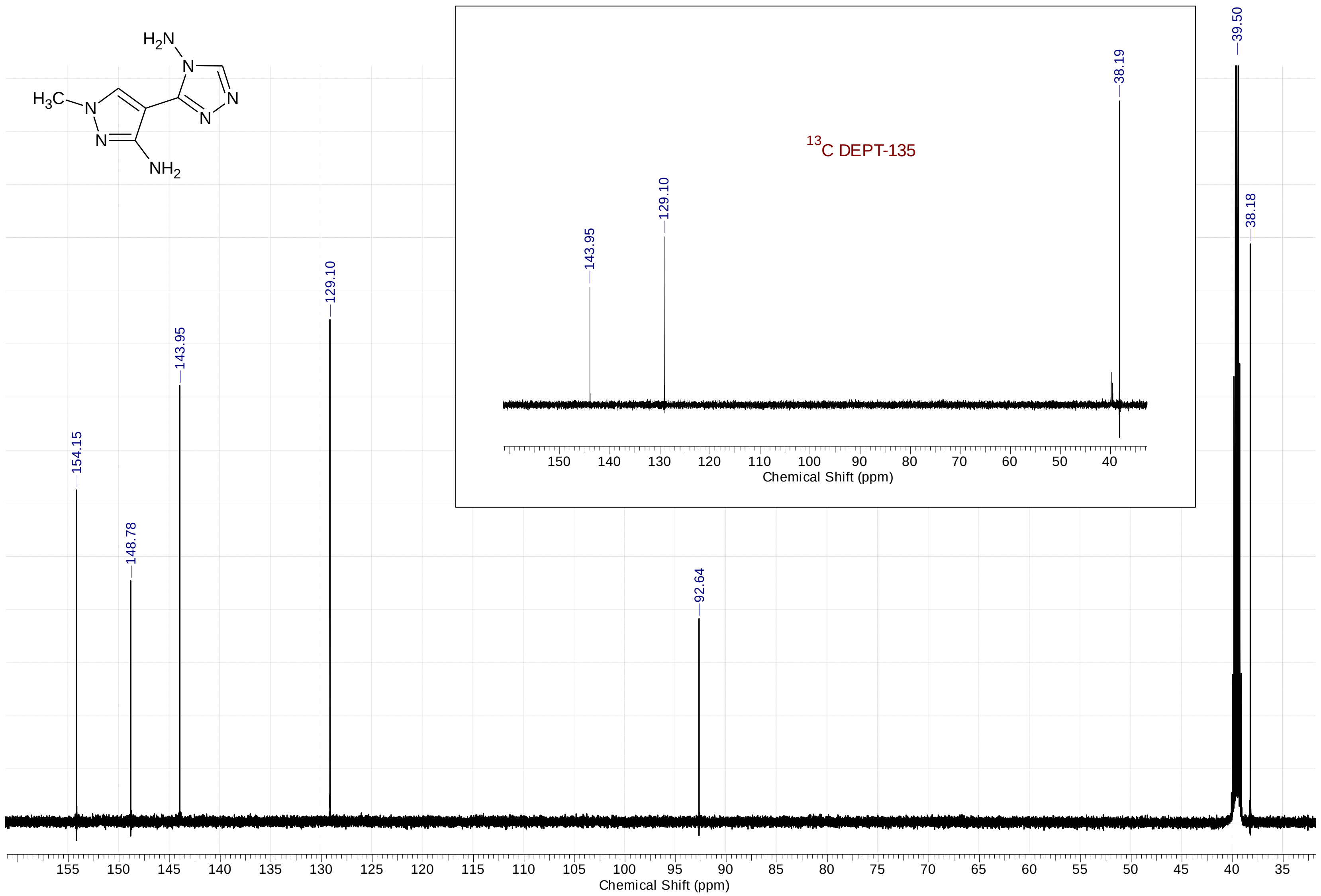

$^{1}H,^{13}C$ HSQC spectrum of compound **10** (Bruker Avance III, 30 °C, DMSO-d_6)

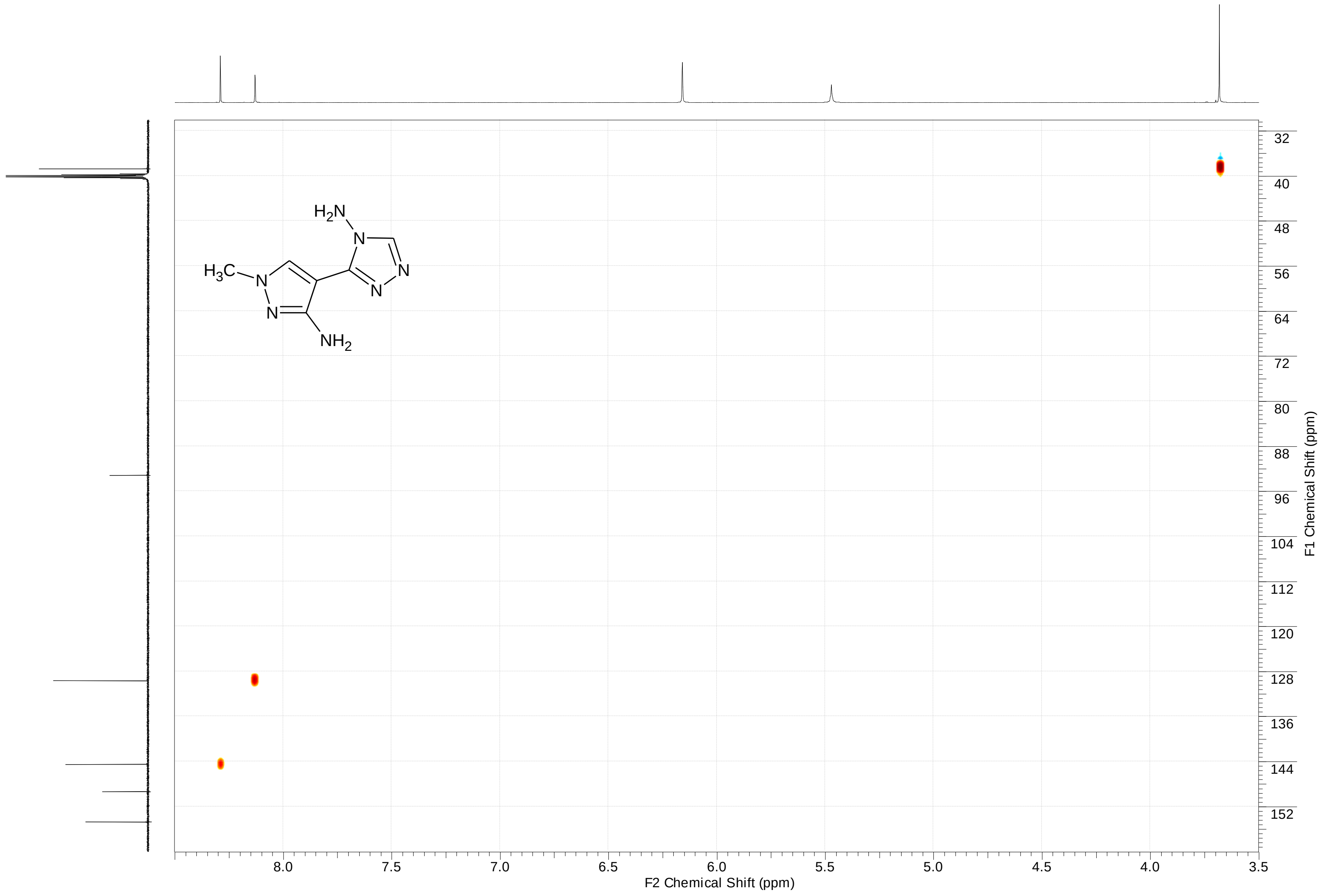

$^1H,^{13}C$ HMBC spectrum of compound **10** (Bruker Avance III, 30 °C, DMSO-d_6)

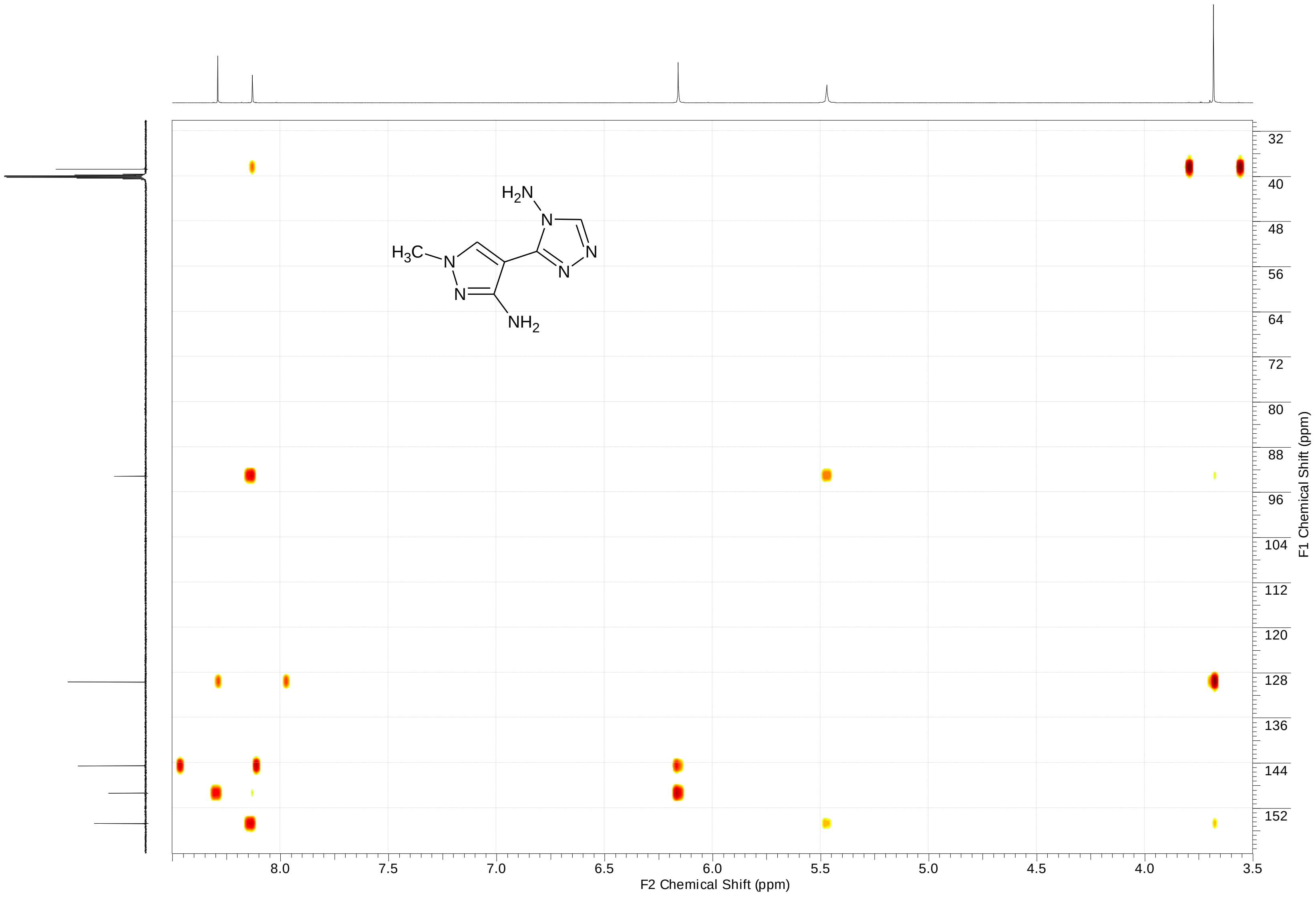

$^{1}H,^{1}H$ NOESY spectrum of compound **10** (600.13 MHz, 30 °C, DMSO-d_6)

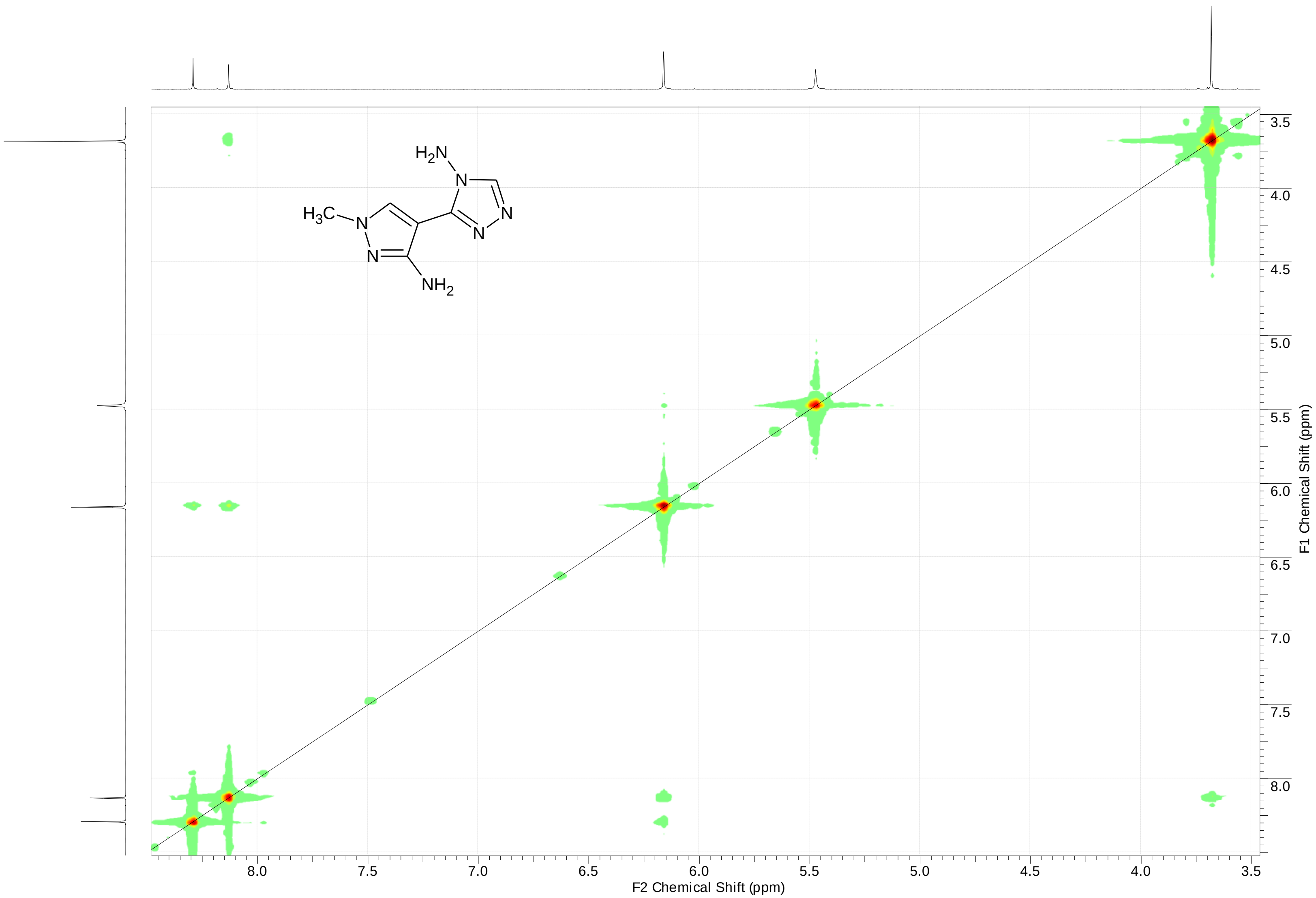

^{1}H NMR spectrum of compound **13** (600.13 MHz, 30 °C, DMSO-d_6).

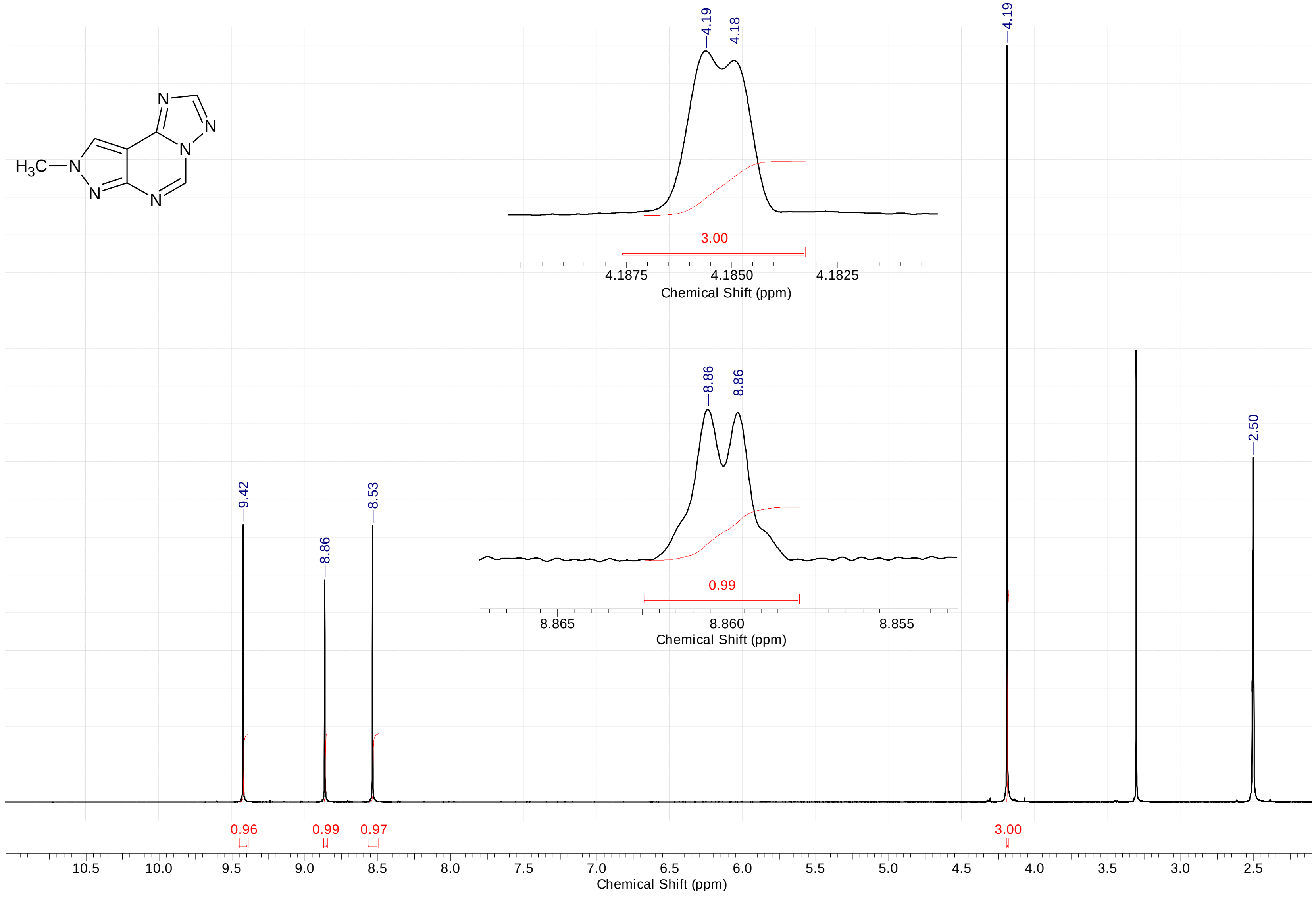

^{13}C NMR spectrum of of compound **13** (150.90 MHz, 30 °C, DMSO-d_6)

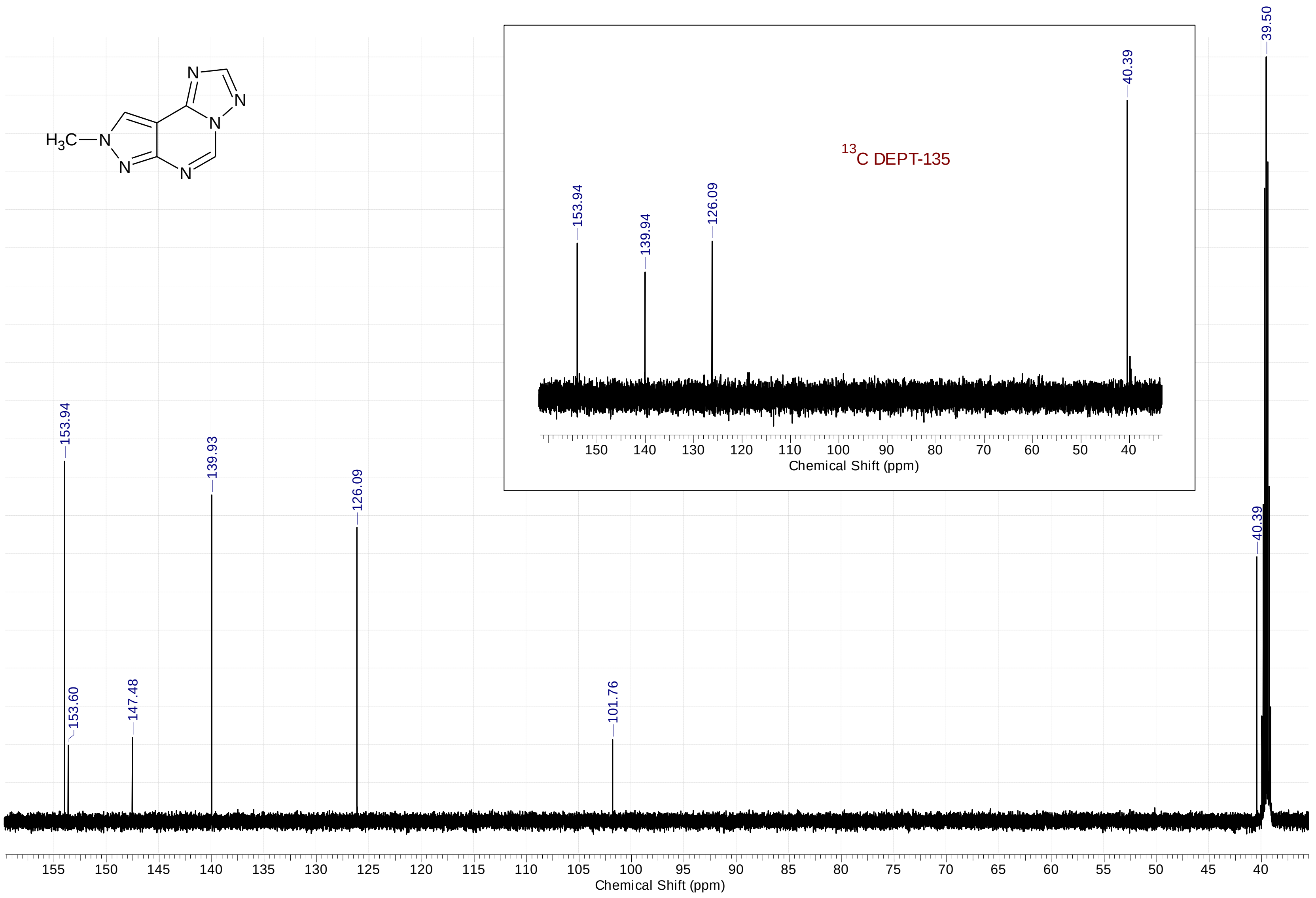

^{1}H NMR spectrum of compound **14** (600.13 MHz, 30 °C, DMSO-d_6).

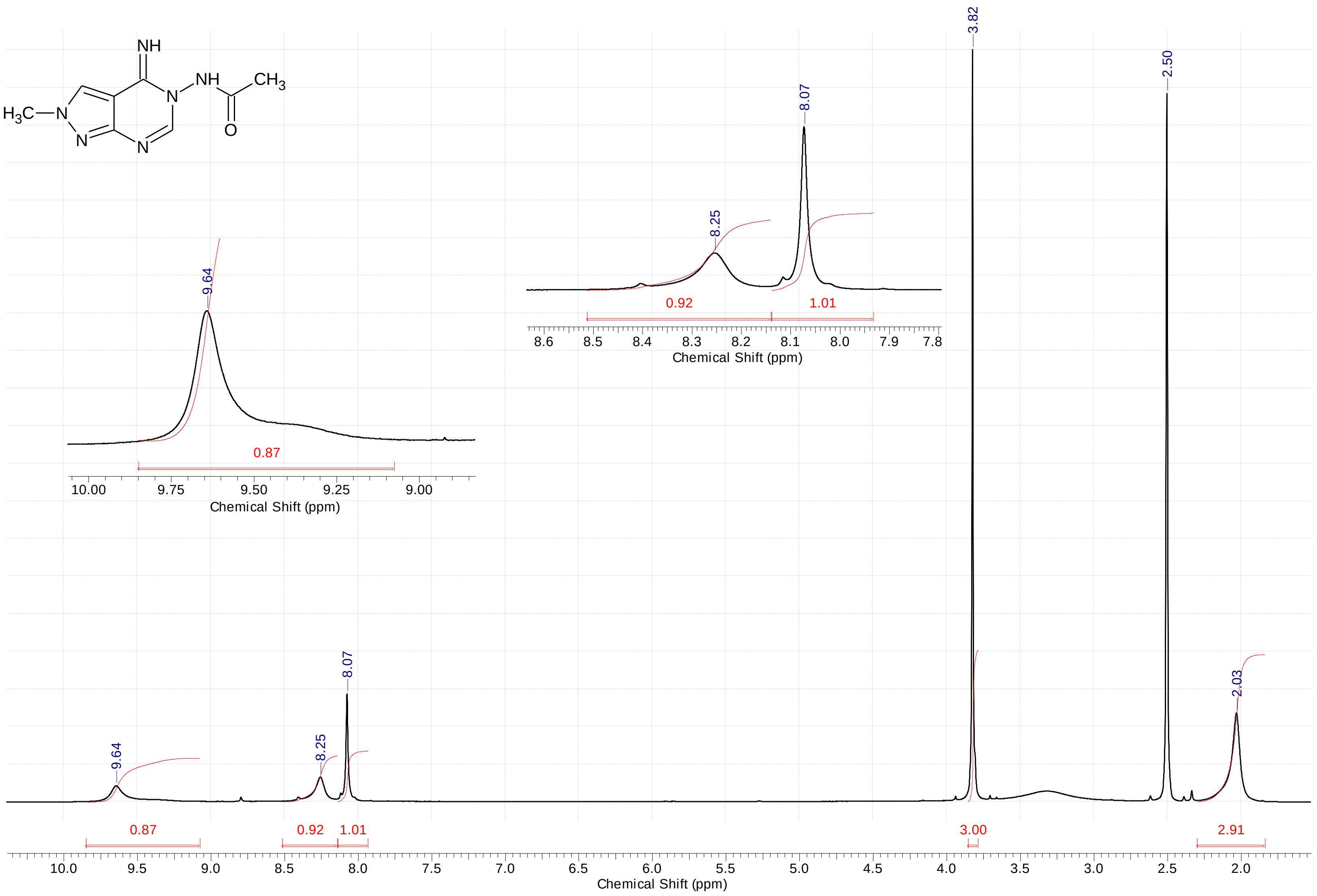

^{1}H NMR spectrum of compound **14** (600.13 MHz, 60 °C, DMSO-d_6).

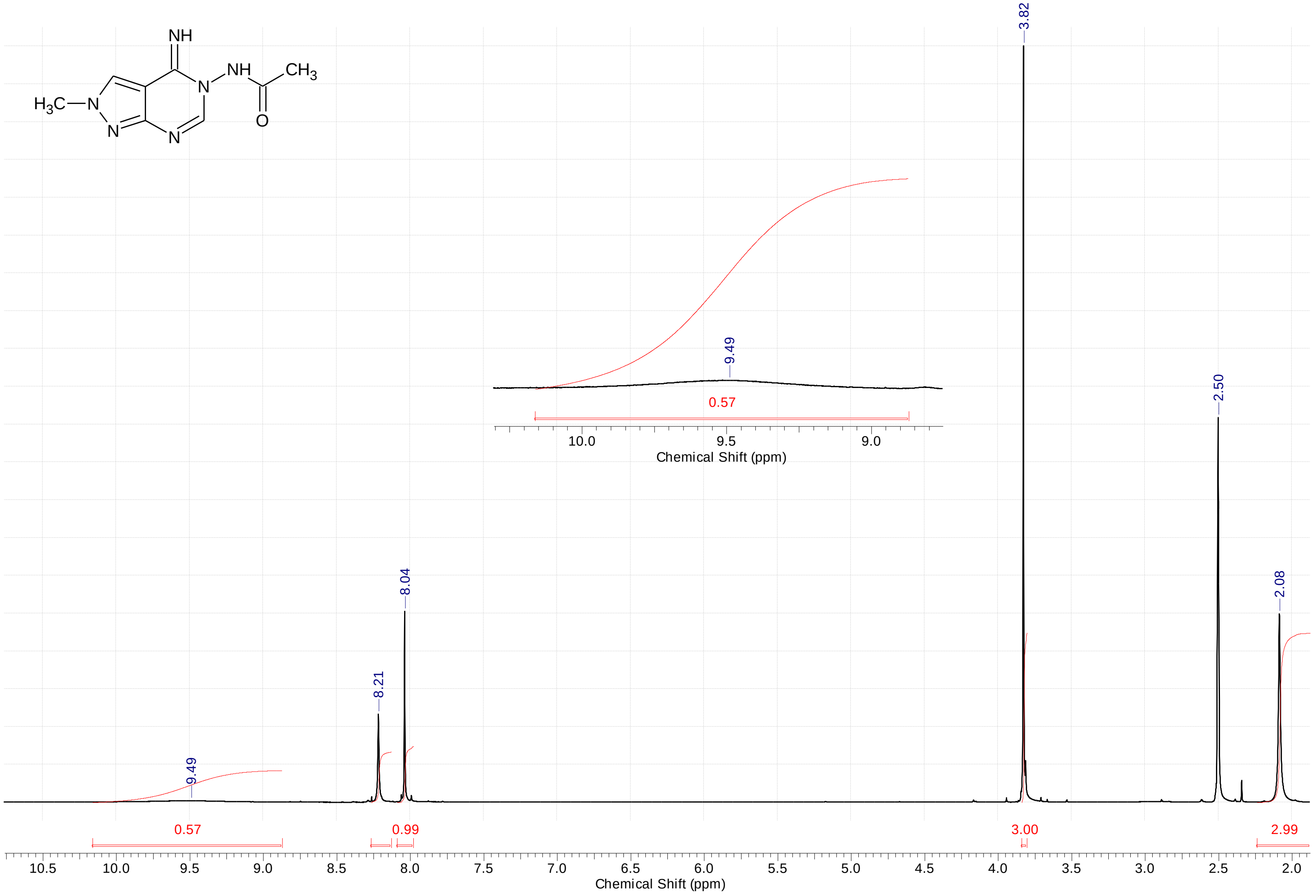

^{1}H NMR spectrum of compound **14** (600.13 MHz, 85 °C, DMSO-d_6).

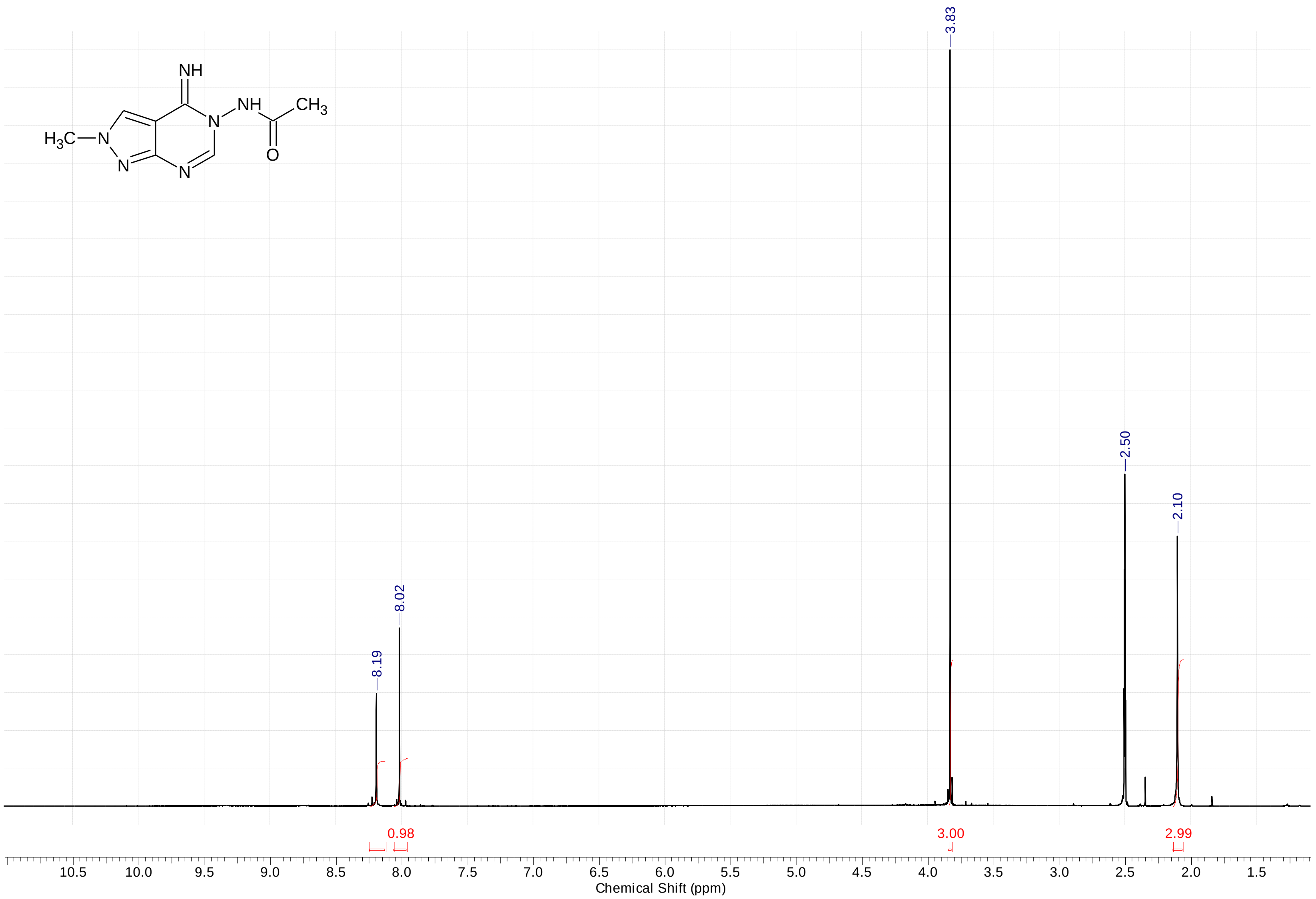

^{13}C NMR spectrum of of compound **14** (150.90 MHz, 85 °C, DMSO-d_6)

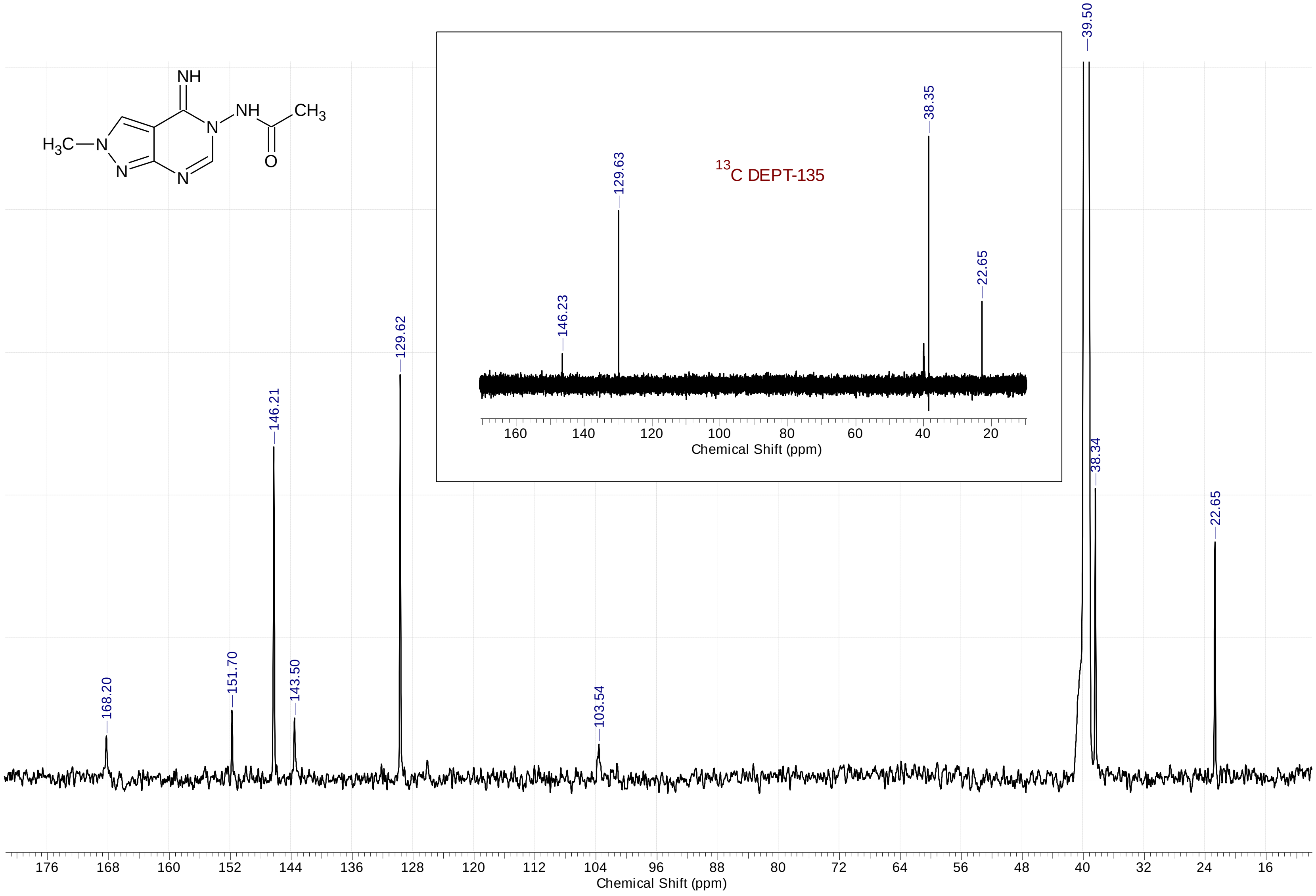

^{1}H,^{13}C HSQC spectrum of compound **14** (Bruker Avance III, 85 °C, DMSO-d_6)

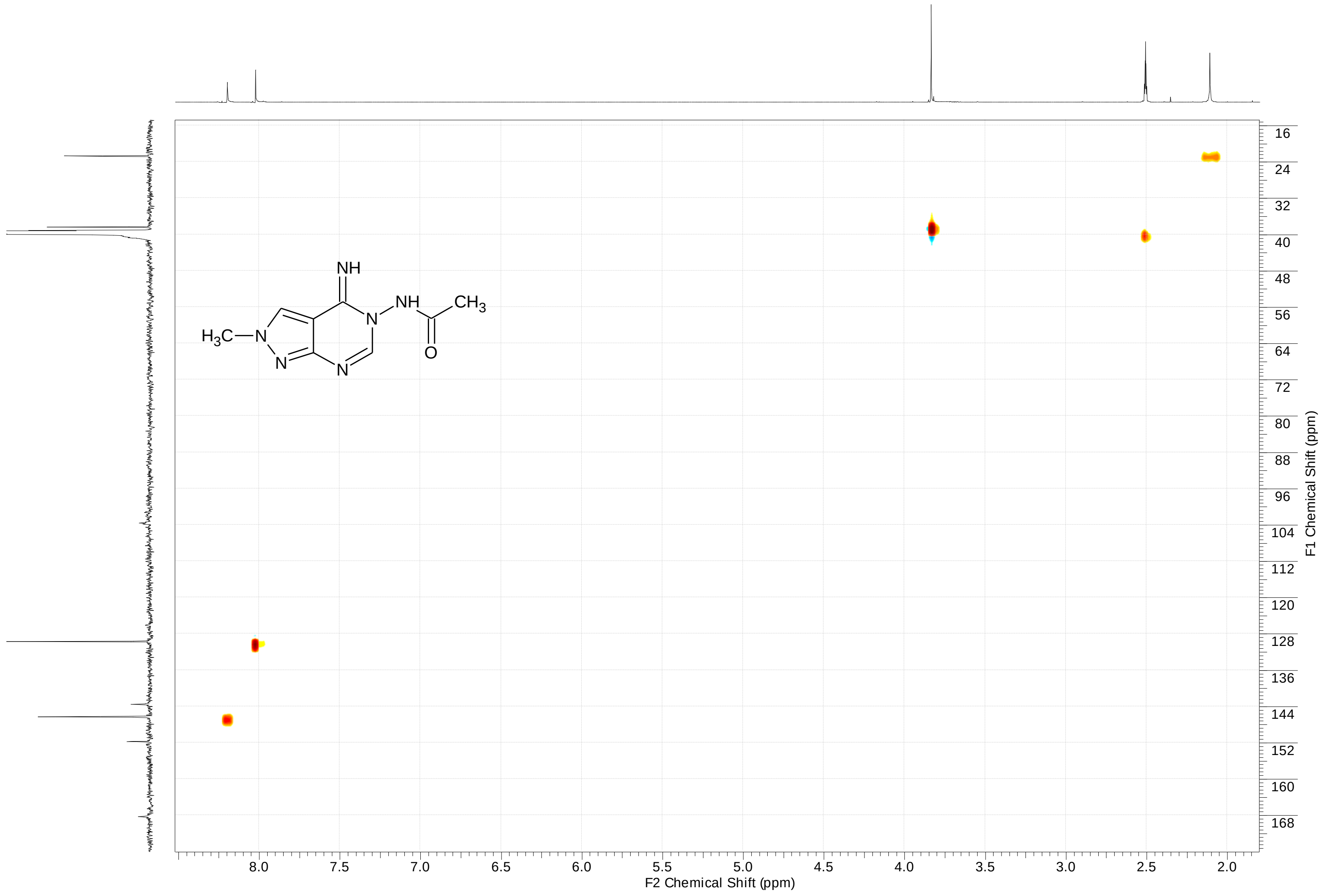

Fragment of $^{1}H,^{13}C$ HMBC spectrum of compound **14** (Bruker Avance III, 85 °C, DMSO-d_6)

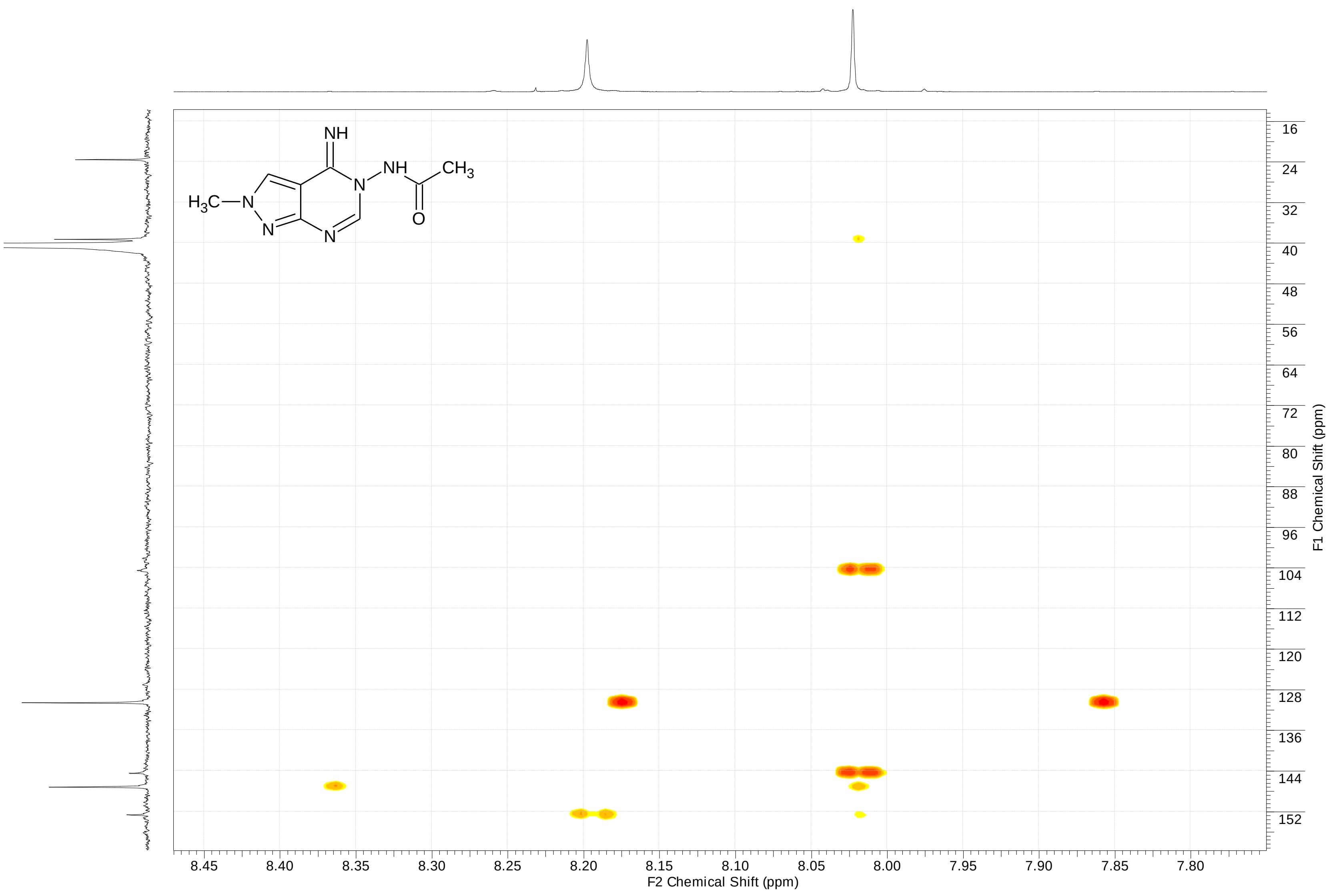

Fragment of $^{1}H,^{13}C$ HMBC spectrum of compound **14** (Bruker Avance III, 85 °C, DMSO-d_6)

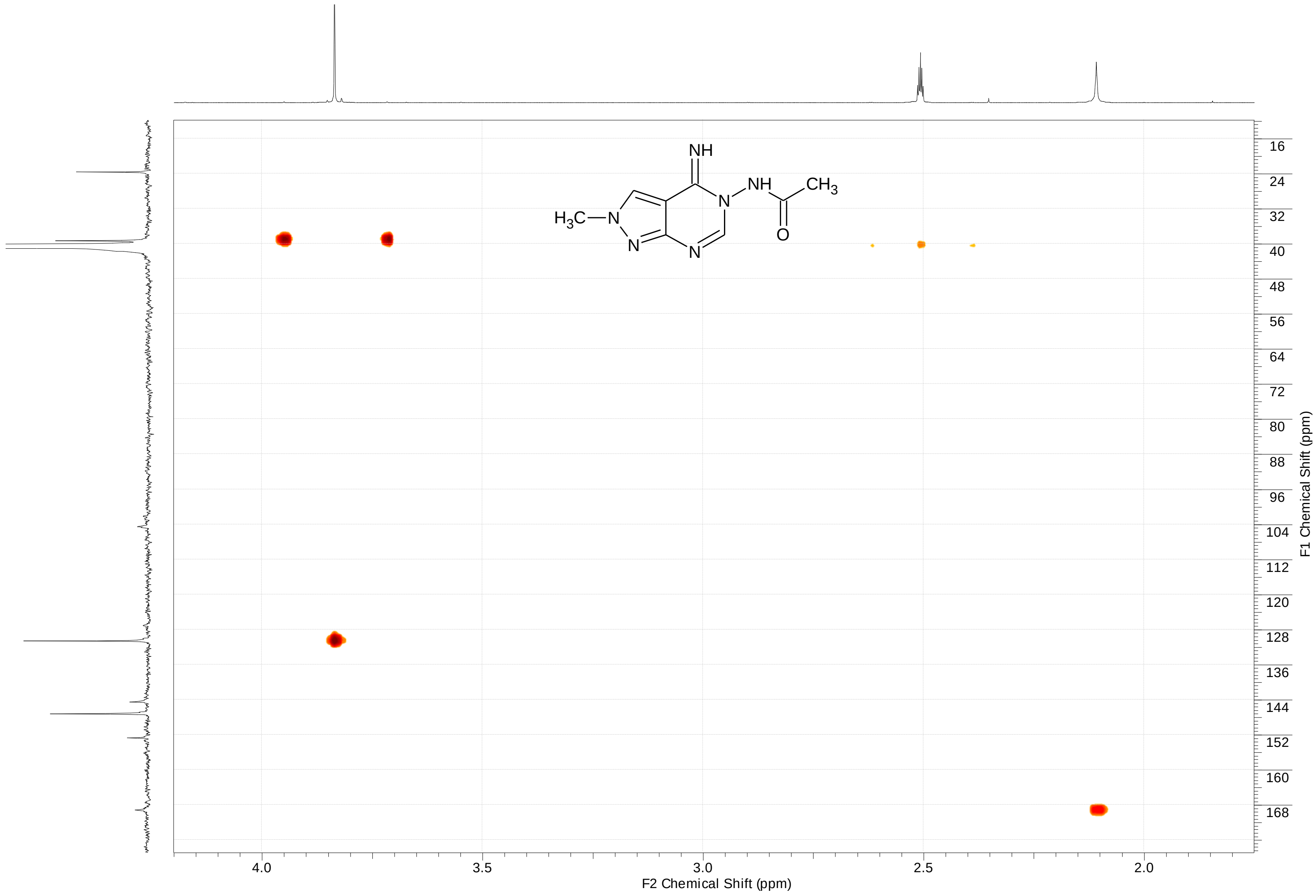

IR spectrum of compound **5** (KBr pellet).

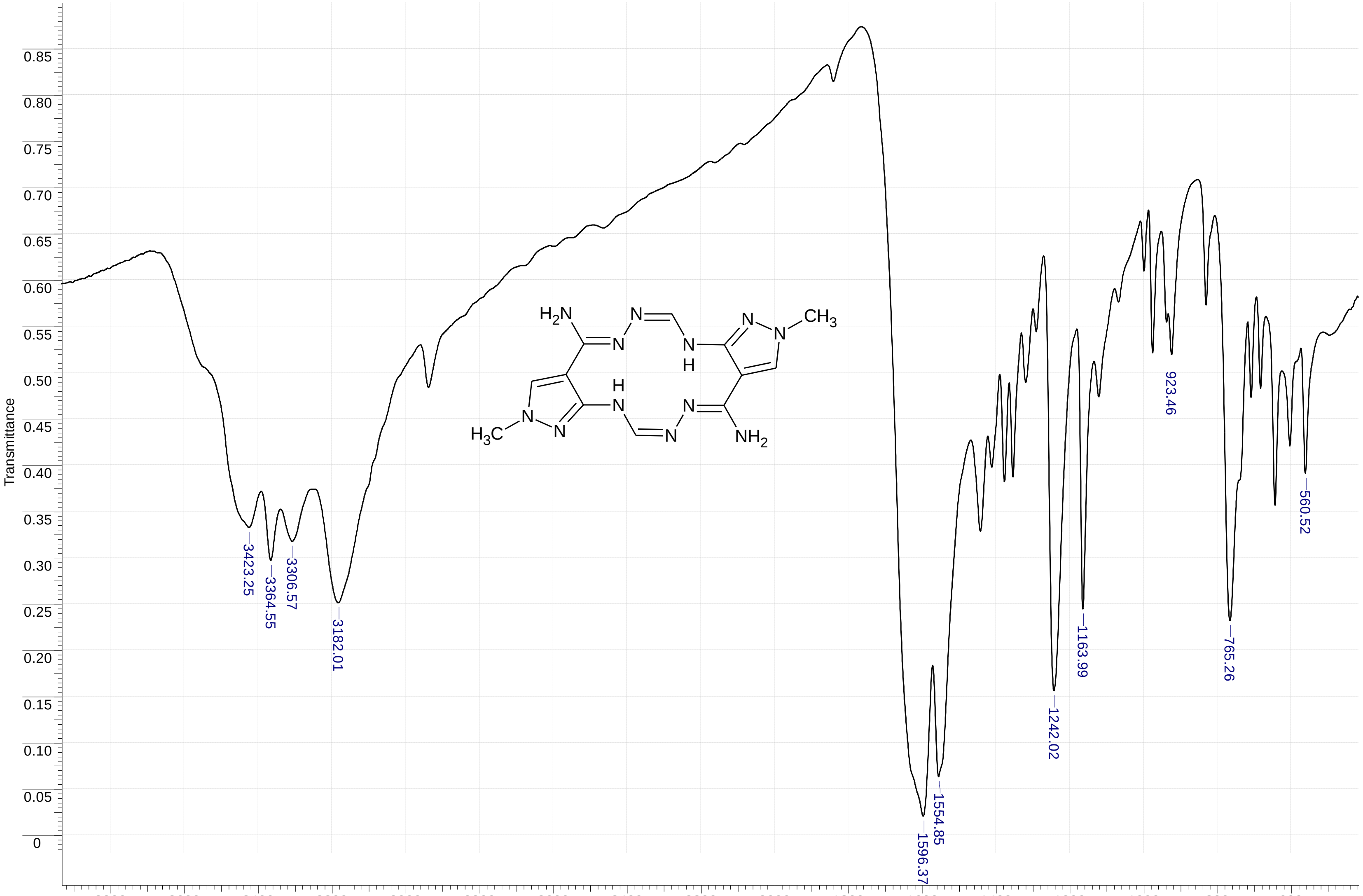

IR spectrum of compound **8** (KBr pellet).

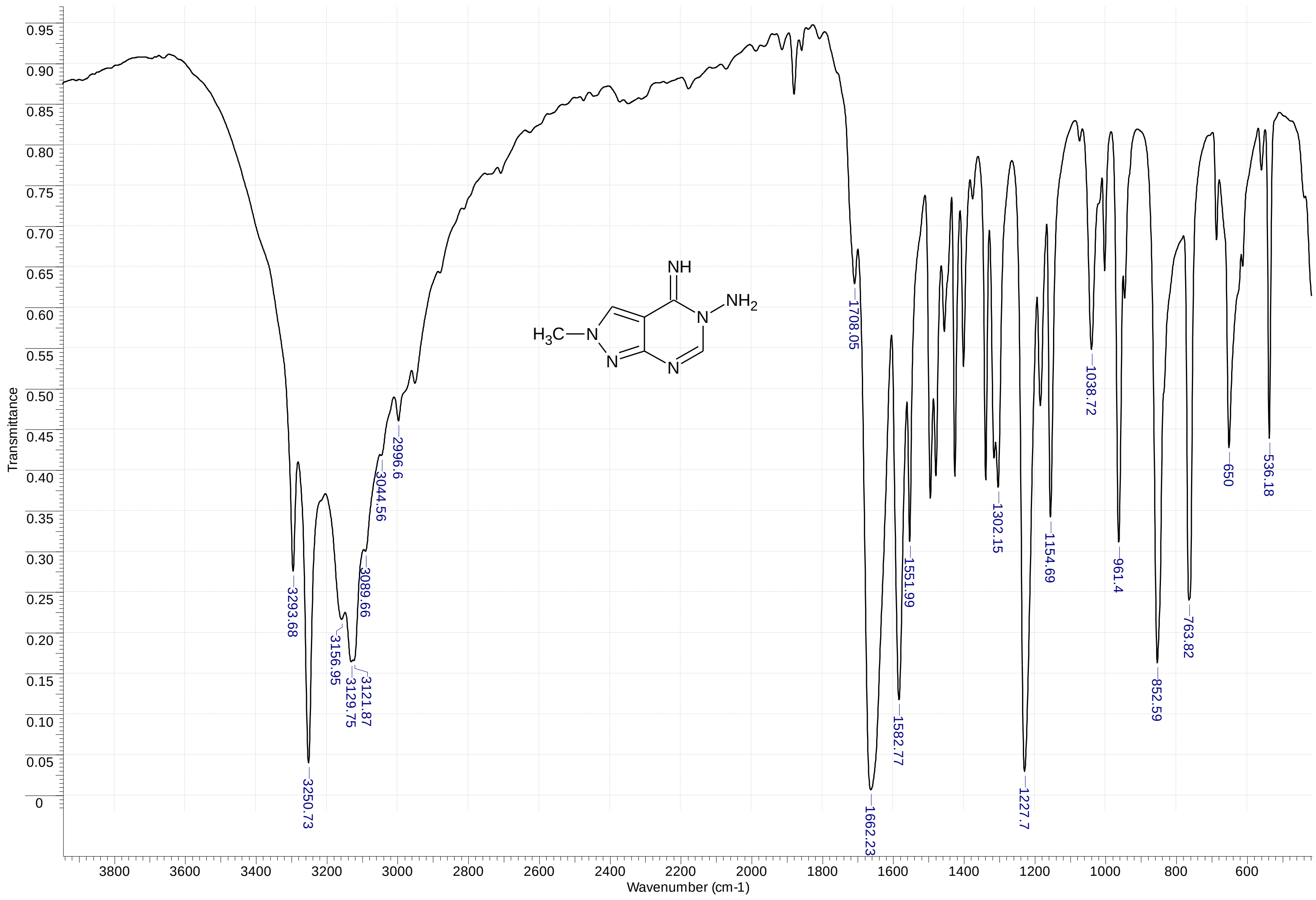

IR spectrum of compound **10** (KBr pellet).

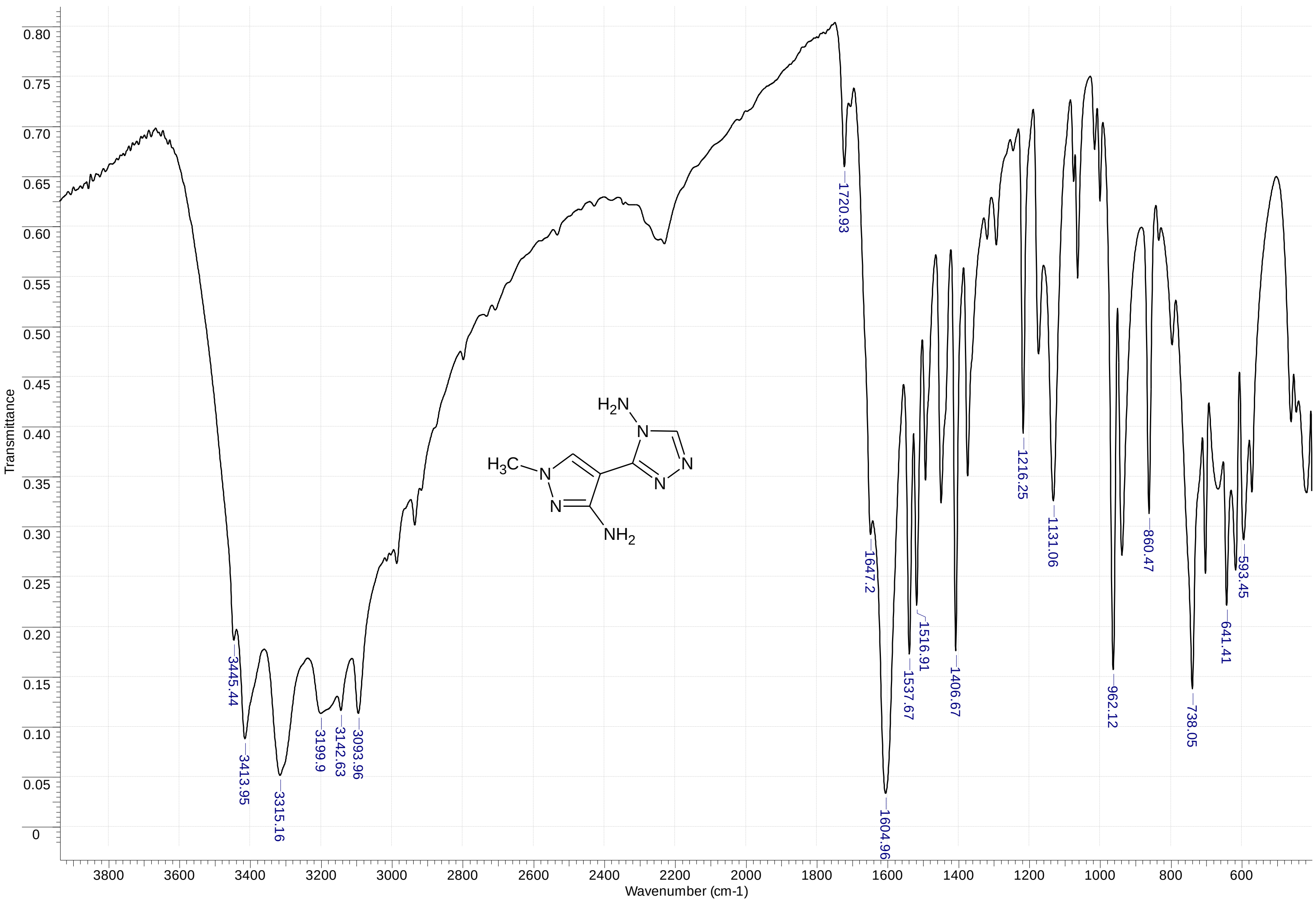

IR spectrum of compound **13** (KBr pellet).

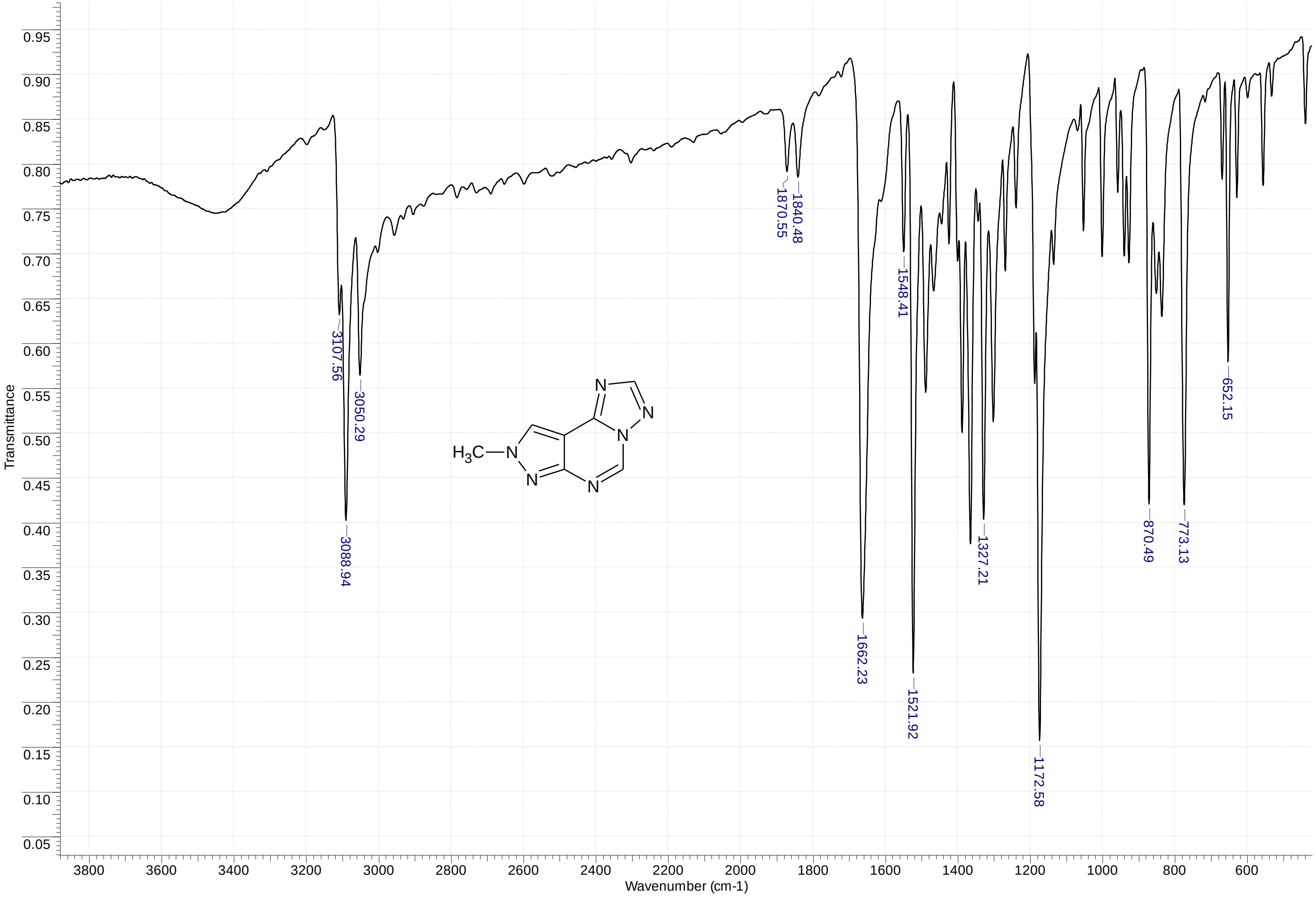

IR spectrum of compound **14** (KBr pellet).

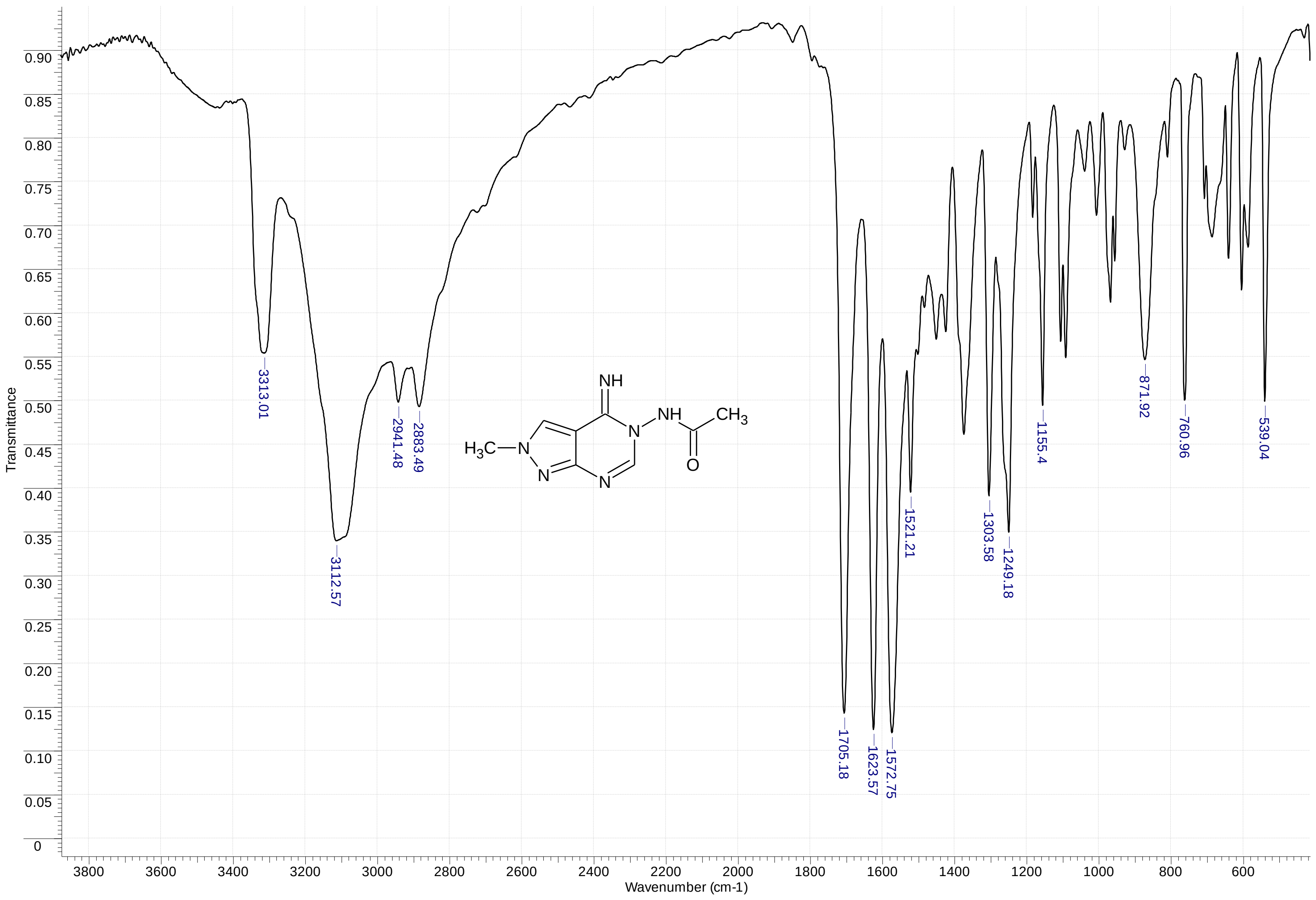

Computational details

The geometry optimizations were carried out at the B3LYP level of theory using Gaussian 16 suite[1] of quantum chemical programs. Pople's basis sets, 6-311++G(d,p), was employed for geometry optimization. The effect of continuum solvation was incorporated by using the polarizable continuum model (PCM). Enthalpies and Gibbs free energies were obtained by adding unscaled zero-point vibrational energy corrections (ZPVE) and thermal contributions to the energies (temperature 298.150 Kelvin, pressure 1.000 atm). Carbon chemical shifts of the prepared compounds in DMSO were calculated by the GIAO method at the PBE1PBE/6-311+G(2d,p) level of theory using the DFT B3LYP/6-311++G(d,p) optimized geometries (DMSO solution) and applying a multi-standard approach.[2]

DMSO solution

Pyrazolopyrimidine 8

Table S1: Cartesian coordinates and energies of the optimized geometry of *(Z)*-**8**.

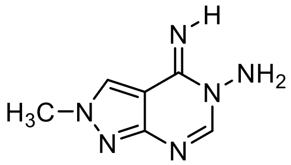

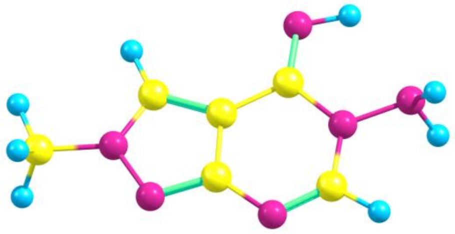

Electronic Energy =	-562.081809772 a.u.
Zero-point correction=	0.156283 (Hartree/Particle)
Thermal correction to Energy=	0.166283
Thermal correction to Enthalpy=	0.167227
Thermal correction to Gibbs Free Energy=	0.120815
Sum of electronic and zero-point Energies=	-561.925527
Sum of electronic and thermal Energies=	-561.915527
Sum of electronic and thermal Enthalpies=	-561.914583
Sum of electronic and thermal Free Energies=	-561.960995

Standard orientation:

Center Number	Atomic Number	Atomic Type	Coordinates (Angstroms) X	Y	Z
1	6	0	-3.843614	0.277386	-0.000004
2	7	0	-2.395175	0.124791	-0.000001
3	6	0	-1.470688	1.108651	0.000002
4	6	0	-0.234361	0.479153	0.000005
5	6	0	-0.538854	-0.903959	0.000002
6	7	0	-1.856193	-1.118979	-0.000002
7	6	0	1.134464	0.945464	0.000002
8	7	0	1.500151	2.172611	0.000003
9	7	0	2.033945	-0.162888	-0.000001
10	6	0	1.624237	-1.481683	0.000002
11	7	0	0.405521	-1.910371	0.000004
12	7	0	3.406708	0.149333	-0.000008
13	1	0	3.849656	-0.232316	-0.829985
14	1	0	3.849662	-0.232303	0.829972
15	1	0	-4.081474	1.339039	-0.000015
16	1	0	-4.262955	-0.191500	0.890559
17	1	0	-4.262952	-0.191517	-0.890559
18	1	0	-1.748188	2.150003	0.000002
19	1	0	2.520012	2.220061	-0.000001
20	1	0	2.444428	-2.193018	0.000006

Table S2: Cartesian coordinates and energies of the optimized geometry of *(E)*-**8**.

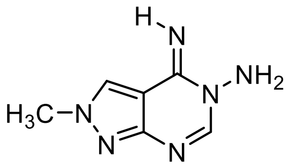

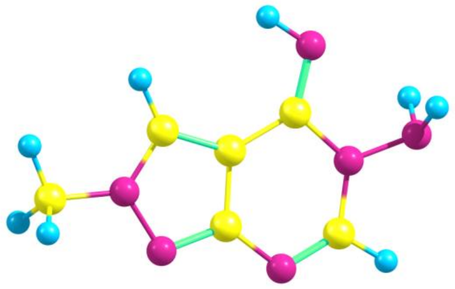

Electronic Energy =	-562.084576037 a.u.
Zero-point correction=	0.156209 (Hartree/Particle)
Thermal correction to Energy=	0.166341
Thermal correction to Enthalpy=	0.167286
Thermal correction to Gibbs Free Energy=	0.120507
Sum of electronic and zero-point Energies=	-561.928367
Sum of electronic and thermal Energies=	-561.918235
Sum of electronic and thermal Enthalpies=	-561.917290
Sum of electronic and thermal Free Energies=	-561.964069

Standard orientation:

Center Number	Atomic Number	Atomic Type	Coordinates (Angstroms) X	Y	Z
1	6	0	-3.830027	0.294008	0.000002
2	7	0	-2.382453	0.130649	0.000000
3	6	0	-1.452388	1.107117	-0.000004
4	6	0	-0.218627	0.468059	-0.000006
5	6	0	-0.534583	-0.915113	-0.000001
6	7	0	-1.853766	-1.117618	0.000002
7	6	0	1.157731	0.912242	-0.000001
8	7	0	1.665566	2.092643	0.000005
9	7	0	2.039228	-0.197529	-0.000002
10	6	0	1.624259	-1.508050	0.000000
11	7	0	0.400247	-1.927294	0.000001
12	7	0	3.434386	0.010678	0.000001
13	1	0	3.658000	0.583332	0.812119
14	1	0	3.658002	0.583336	-0.812115
15	1	0	0.927778	2.793286	0.000004
16	1	0	-4.060494	1.357218	0.000001
17	1	0	-4.251834	-0.172368	0.890601
18	1	0	-4.251837	-0.172371	-0.890593
19	1	0	-1.728220	2.149506	-0.000008
20	1	0	2.447957	-2.212224	0.000001

Pyrazolopyrimidine 9 and its tautomers

Table S3: Cartesian coordinates and energies of the optimized geometry of the s-*cis*-conformer of **9**.

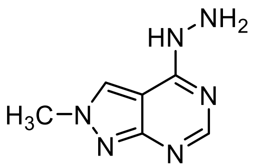

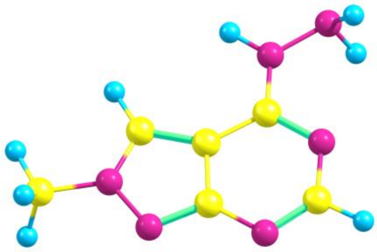

Electronic Energy =	-562.091266870 a.u.
Zero-point correction =	0.155971 (Hartree/Particle)
Thermal correction to Energy =	0.166608
Thermal correction to Enthalpy =	0.167552
Thermal correction to Gibbs Free Energy	0.118851
Sum of electronic and zero-point Energies =	-561.935296
Sum of electronic and thermal Energies =	-561.924659
Sum of electronic and thermal Enthalpies =	-561.923715
Sum of electronic and thermal Free Energies =	-561.972416

Standard orientation:

Center Number	Atomic Number	Atomic Type	Coordinates (Angstroms) X	Y	Z
1	6	0	3.645236	-0.995145	0.000000
2	7	0	2.284030	-0.474248	-0.000001
3	6	0	1.146801	-1.192873	-0.000001
4	6	0	0.106568	-0.262797	0.000000
5	6	0	0.757629	1.004395	0.000001
6	7	0	2.090970	0.866878	0.000000
7	6	0	-1.320038	-0.248517	0.000001
8	7	0	-2.042788	-1.393737	0.000003
9	7	0	-1.959318	0.921069	-0.000001
10	6	0	-1.216302	2.057720	-0.000000
11	7	0	0.092906	2.194163	0.000001
12	7	0	-3.445972	-1.446318	-0.000002
13	1	0	-3.792025	-0.946487	0.816120
14	1	0	-3.792019	-0.946485	-0.816124
15	1	0	-1.579688	-2.288103	-0.000000
16	1	0	3.603446	-2.082178	-0.000009
17	1	0	4.168431	-0.646558	0.890471
18	1	0	4.168437	-0.646543	-0.890462
19	1	0	1.163550	-2.270705	-0.000003
20	1	0	-1.798289	2.975704	-0.000002

Table S4: Cartesian coordinates and energies of the optimized geometry of the s-*trans*-conformer of **9**.

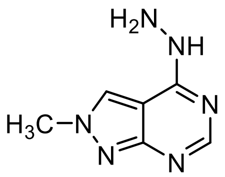

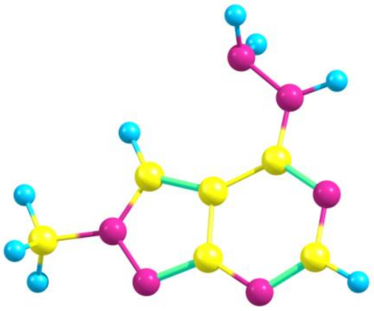

Electronic Energy =	-562.092165994 a.u.
Zero-point correction =	0.156247 (Hartree/Particle)
Thermal correction to Energy =	0.166504
Thermal correction to Enthalpy =	0.167448
Thermal correction to Gibbs Free Energy =	0.120212
Sum of electronic and zero-point Energies =	-561.935919
Sum of electronic and thermal Energies =	-561.925662
Sum of electronic and thermal Enthalpies =	-561.924718
Sum of electronic and thermal Free Energies =	-561.971954

Standard orientation:

Center Number	Atomic Number	Atomic Type	Coordinates (Angstroms) X	Y	Z
1	6	0	-3.464035	0.867026	0.000006
2	7	0	-2.103467	0.346325	-0.000007
3	6	0	-0.967949	1.063548	-0.000048
4	6	0	0.074471	0.133470	-0.000052
5	6	0	-0.579003	-1.136666	-0.000001
6	7	0	-1.912042	-0.998219	0.000022
7	6	0	1.497470	0.114205	-0.000048
8	7	0	2.271111	1.220868	-0.000155
9	7	0	2.133678	-1.063971	0.000010
10	6	0	1.391828	-2.194858	0.000033
11	7	0	0.079687	-2.328644	0.000030
12	7	0	1.720813	2.508474	0.000106
13	1	0	2.036982	3.012034	0.824198
14	1	0	2.037152	3.012404	-0.823689
15	1	0	-3.421912	1.954132	-0.000035
16	1	0	-3.988149	0.518821	0.890273
17	1	0	-3.988190	0.518753	-0.890209
18	1	0	-0.966975	2.139169	-0.000068
19	1	0	3.275233	1.085618	0.000079
20	1	0	1.970695	-3.115110	0.000067

Table S5: Cartesian coordinates and energies of the optimized geometry of the tautomer of **9** (*Z*-isomer).

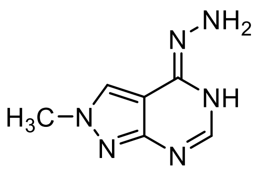

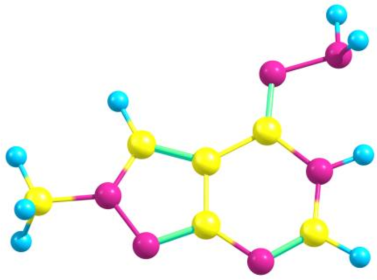

Electronic Energy =	-562.086372220 a.u.
Zero-point correction =	0.156180 (Hartree/Particle)
Thermal correction to Energy =	0.166420
Thermal correction to Enthalpy =	0.167364
Thermal correction to Gibbs Free Energy =	0.120204
Sum of electronic and zero-point Energies =	-561.930192
Sum of electronic and thermal Energies =	-561.919953
Sum of electronic and thermal Enthalpies =	-561.919008
Sum of electronic and thermal Free Energies =	-561.966168

Standard orientation:

Center Number	Atomic Number	Atomic Type	Coordinates (Angstroms) X	Y	Z
1	6	0	3.683026	-0.936990	0.012080
2	7	0	2.308505	-0.456556	0.004749
3	6	0	1.182250	-1.203774	-0.010430
4	6	0	0.123670	-0.308930	-0.013013
5	6	0	0.736182	0.971178	0.003726
6	7	0	2.067707	0.876529	0.014100
7	6	0	-1.316699	-0.411011	-0.020737
8	7	0	-1.972716	-1.521698	-0.032268
9	7	0	-1.920543	0.854437	-0.008842
10	6	0	-1.234275	2.041053	-0.012674
11	7	0	0.052642	2.173108	0.004284
12	7	0	-3.397791	-1.335045	-0.028477
13	1	0	3.671091	-2.025013	0.006047
14	1	0	4.192793	-0.582004	0.908258
15	1	0	4.205825	-0.572485	-0.872725
16	1	0	1.215692	-2.280817	-0.015826
17	1	0	-2.929609	0.868855	-0.096538
18	1	0	-1.864555	2.923944	-0.026270
19	1	0	-3.795381	-2.239769	-0.257133
20	1	0	-3.705404	-1.137292	0.925650

Table S6: Cartesian coordinates and energies of the optimized geometry of the tautomer of **9** (*E*-isomer).

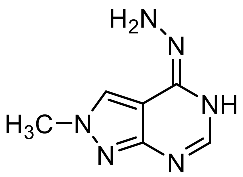
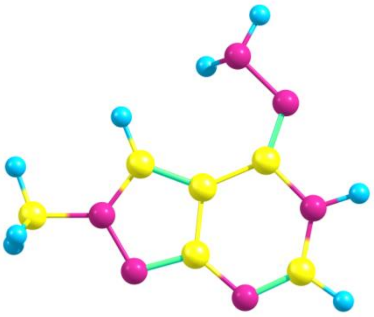

Electronic Energy =	-562.085705132 a.u.
Zero-point correction =	0.156372 (Hartree/Particle)
Thermal correction to Energy =	0.166494
Thermal correction to Enthalpy =	0.167438
Thermal correction to Gibbs Free Energy =	0.120683
Sum of electronic and zero-point Energies =	-561.929333
Sum of electronic and thermal Energies =	-561.919211
Sum of electronic and thermal Enthalpies =	-561.918267
Sum of electronic and thermal Free Energies =	-561.965023

Standard orientation:

Center Number	Atomic Number	Atomic Type	Coordinates (Angstroms) X	Y	Z
1	6	0	-3.516963	0.680694	0.026044
2	7	0	-2.125531	0.253737	0.009123
3	6	0	-1.028777	1.039977	0.023537
4	6	0	0.068691	0.185518	-0.002888
5	6	0	-0.503335	-1.120311	-0.027126
6	7	0	-1.834210	-1.071661	-0.021451
7	6	0	1.508185	0.327713	0.022768
8	7	0	2.268886	1.364382	0.035989
9	7	0	2.146033	-0.929970	0.035310
10	6	0	1.499825	-2.127908	-0.003974
11	7	0	0.214656	-2.300533	-0.039649
12	7	0	1.606036	2.612957	0.039089
13	1	0	3.157279	-0.923131	0.054411
14	1	0	2.322115	3.318378	-0.091440
15	1	0	0.966148	2.697057	-0.752016
16	1	0	-3.547659	1.768068	0.054126
17	1	0	-4.015626	0.278420	0.908514
18	1	0	-4.023041	0.324306	-0.871826
19	1	0	-1.111328	2.112652	0.062835
20	1	0	2.155264	-2.992234	-0.003654

Table S7. Relative electronic (ΔE, kcal/mol) and Gibbs free energies (ΔG, kcal/mol) of pyrazolopyrimidines **8**, **9**, and tautomers of **9** in DMSO solution.[a]

Compound	ΔE	ΔG
(Z)-**8**	6.50	7.17
(E)-**8**	4.76	5.24
s-*cis*-conformer of **9**	0.56	0.00
s-*trans*-conformer of **9**	0.00	0.29
tautomer of **9** (*Z*-isomer)	3.64	3.92
tautomer of **9** (*E*-isomer)	4.05	4.64

[a] Calculated using data of Tables S1-S6.

Table S8. Experimental ^{13}C chemical shifts (in DMSO-d_6) for the product of the reaction of imidate **4** with $N_2H_4{\cdot}H_2O$ in EtOH and calculated ^{13}C chemical shifts for (*E*)- and *(Z)*-**8**, s-*cis*- and s-*trans*-conformers of **9** in DMSO solution.[a]

^{13}C assign-ment	Calculated shift, ppm				Observed shift, ppm
	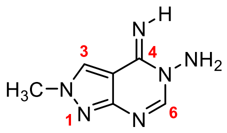 (*Z*)-**8**	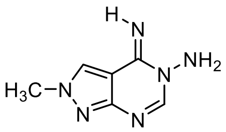 (*E*)-**8**	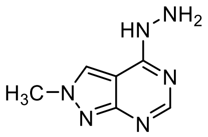 **9** s-*cis*-conformer	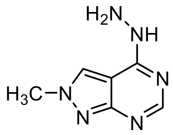 **9** s-*trans*-conformer	
C(7a)	156.6	156.8	160.9	162.1	155.0
C(4)	152.1	157.2	158.9	160.9	151.8
C(6)	151.9	152.1	157.4	157.1	149.9
C(3)	128.3	127.9	123.3	128.8	128.4
C(3a)	105.6	104.1	98.4	98.3	105.4

[a] Calculations were performed for sp^2-atoms by the GIAO method at the PBE1PBE/6-311+G(2d,p) level of theory using the DFT B3LYP/6-311++G(d,p) optimized geometries (DMSO solution) and applying a multi-standard approach (C_6H_6 as a standard; δ_{calc} = 182.4059 - δ_{abs}, in ppm).

Macrocycle 5

Table S9: Cartesian coordinates and energies of the optimized geometry of macrocycle **5**.

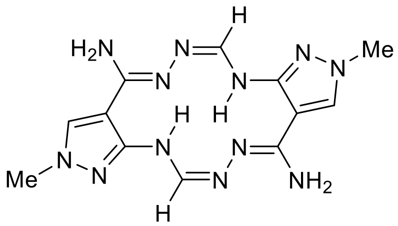

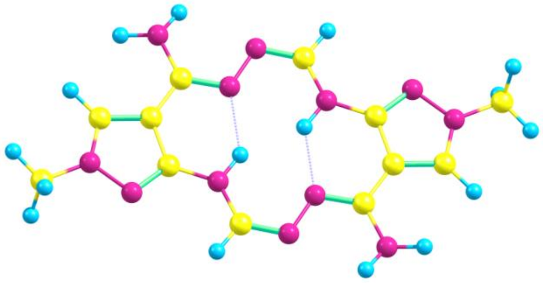

Electronic Energy =	-1124.19783035 a.u.
Zero-point correction =	0.312391 (Hartree/Particle)
Thermal correction to Energy =	0.334428
Thermal correction to Enthalpy =	0.335372
Thermal correction to Gibbs Free Energy =	0.260484
Sum of electronic and zero-point Energies =	-1123.885439
Sum of electronic and thermal Energies =	-1123.863402
Sum of electronic and thermal Enthalpies =	-1123.862458
Sum of electronic and thermal Free Energies =	-1123.937347

Standard orientation:

Center Number	Atomic Number	Atomic Type	Coordinates (Angstroms) X	Y	Z
1	7	0	0.980495	-1.603095	0.017727
2	6	0	2.259932	-1.883020	-0.008959
3	7	0	2.750144	-3.158694	0.015280
4	6	0	3.195414	-0.759746	-0.011109
5	7	0	0.137247	-2.715127	-0.002910
6	6	0	-1.118689	-2.389281	0.027152
7	7	0	-1.575840	-1.106658	0.084893
8	6	0	-2.870386	-0.633070	0.045526
9	6	0	2.870387	0.633070	-0.045509
10	7	0	1.575841	1.106658	-0.084875
11	6	0	1.118689	2.389281	-0.027173
12	7	0	-0.137248	2.715128	0.002880
13	7	0	-0.980495	1.603095	-0.017722
14	6	0	-2.259931	1.883020	0.008932
15	6	0	-3.195415	0.759746	0.011107
16	7	0	-3.953391	-1.400020	0.032612
17	7	0	-4.995682	-0.521096	-0.013502
18	6	0	-4.587289	0.759758	-0.029059
19	6	0	-6.358313	-1.025641	-0.043609
20	7	0	3.953392	1.400019	-0.032583
21	7	0	4.995683	0.521095	0.013518
22	6	0	4.587289	-0.759760	0.029063
23	6	0	6.358312	1.025640	0.043687
24	1	0	3.678090	-3.339259	-0.333343
25	1	0	2.064124	-3.883290	-0.146548
26	1	0	-0.894081	-0.350053	0.079181
27	1	0	0.894082	0.350052	-0.079156
28	1	0	-1.856982	-3.181994	0.005178
29	1	0	1.856981	3.181997	-0.005223
30	1	0	-5.299543	1.568048	-0.075313
31	1	0	-6.556783	-1.615607	0.852417
32	1	0	-7.041115	-0.178793	-0.078329
33	1	0	-6.505884	-1.648003	-0.927454
34	1	0	5.299541	-1.568051	0.075312
35	1	0	6.556770	1.615726	-0.852262
36	1	0	7.041117	0.178789	0.078284
37	1	0	6.505892	1.647884	0.927614
38	7	0	-2.750144	3.158695	-0.015369
39	1	0	-2.064127	3.883294	0.146457
40	1	0	-3.678094	3.339272	0.333240

Table S10. Experimental and calculated ^{13}C chemical shifts for macrocycle **5** in DMSO solution.[a]

^{13}C assignment	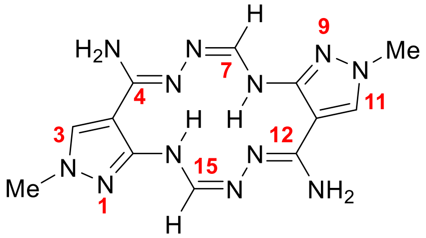	
	Calculated shift, ppm	Observed shift, ppm
C4 and C12	152.8	152.3
C8a and C16a	148.6	147.6
C7 and C15	138.1	138.2
C3 and C11	129.2	130.7
C3a and C11a	99.6	100.5

[a] Calculations were performed for sp^2-atoms by the GIAO method at the PBE1PBE/6-311+G(2d,p) level of theory using the DFT B3LYP/6-311++G(d,p) optimized geometries (DMSO solution) and applying a multi-standard approach (C_6H_6 as a standard; δ_{calc} = 182.4059 - δ_{abs}, in ppm).

Bis-pyrazole 6

Table S11: Cartesian coordinates and energies of the optimized geometry of bis-pyrazole **6**.

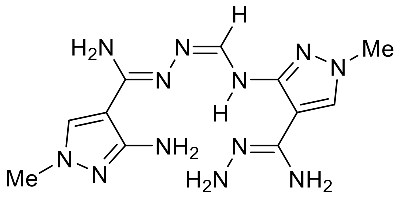

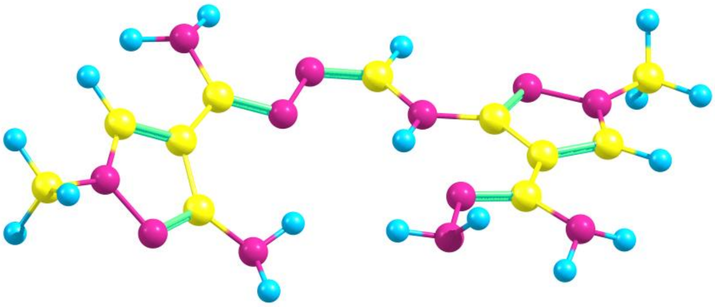

Electronic Energy =	-1087.29434685 a.u.
Zero-point correction =	0.328355 (Hartree/Particle)
Thermal correction to Energy =	0.351721
Thermal correction to Enthalpy =	0.352665
Thermal correction to Gibbs Free Energy =	0.273354
Sum of electronic and zero-point Energies =	-1086.965991
Sum of electronic and thermal Energies =	-1086.942626
Sum of electronic and thermal Enthalpies =	-1086.941682
Sum of electronic and thermal Free Energies =	-1087.020993

Standard orientation:

Center Number	Atomic Number	Atomic Type	Coordinates (Angstroms) X	Y	Z
1	7	0	-5.276614	0.544039	-0.150312
2	6	0	-4.697822	-0.609782	-0.516996
3	6	0	-3.407231	-0.619049	0.009105
4	6	0	-3.311904	0.627624	0.701218
5	7	0	-4.442550	1.324653	0.600388
6	6	0	-6.607070	1.023887	-0.478327
7	7	0	-2.246032	1.070663	1.458057
8	6	0	-2.389142	-1.651529	-0.115300
9	7	0	-2.776697	-2.887315	-0.572517
10	7	0	-1.147034	-1.346969	0.165031
11	7	0	-0.292473	-2.450147	0.110599
12	6	0	0.962830	-2.147334	0.083662
13	7	0	1.486031	-0.885561	0.039721
14	6	0	2.837895	-0.590643	0.017701
15	6	0	3.393261	0.717201	-0.098000
16	6	0	4.765836	0.488385	-0.078549
17	7	0	4.955814	-0.837938	0.043711
18	7	0	3.781882	-1.523553	0.107498
19	6	0	2.670716	1.980624	-0.214568
20	7	0	3.399499	3.155086	-0.303541
21	7	0	1.376986	1.943316	-0.289914
22	7	0	0.754689	3.215188	-0.388046
23	6	0	6.214531	-1.560755	0.112968
24	1	0	-1.367120	0.643975	1.189885
25	1	0	-2.067851	-3.603375	-0.477288
26	1	0	-3.722503	-3.183191	-0.384550
27	1	0	0.867020	-0.081739	-0.039026
28	1	0	4.338759	3.131196	0.066399
29	1	0	2.890475	3.982126	-0.013921
30	1	0	1.064343	3.706906	-1.228651
31	1	0	-0.237711	3.044067	-0.502004
32	1	0	-5.217123	-1.327438	-1.132293
33	1	0	-7.117397	0.262208	-1.065314
34	1	0	-6.541542	1.945608	-1.059379
35	1	0	-7.171208	1.214147	0.436248
36	1	0	-2.205560	2.070979	1.596902
37	1	0	1.680704	-2.958603	0.093010
38	1	0	5.601908	1.165389	-0.152486
39	1	0	7.031362	-0.844711	0.043757
40	1	0	6.283428	-2.270375	-0.712972
41	1	0	6.284113	-2.099173	1.059472

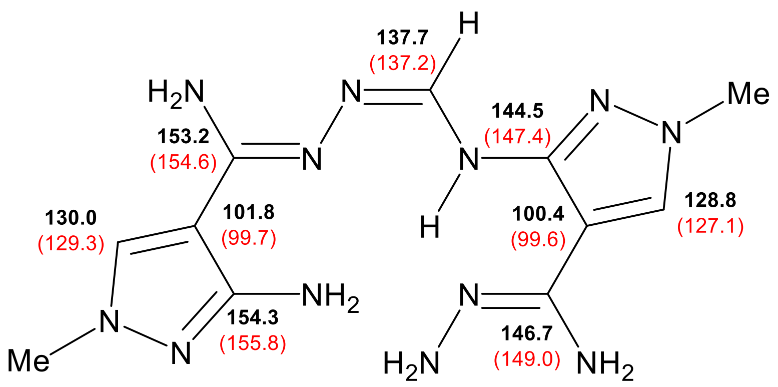

Figure S1. Experimental and calculated (numbers in red) ^{13}C chemical shifts for bis-pyrazole **6** in DMSO solution. Calculations were performed for sp^2-atoms by the GIAO method at the PBE1PBE/6-311+G(2d,p) level of theory using the DFT B3LYP/6-311++G(d,p) optimized geometries (DMSO solution) and applying a multi-standard approach (C_6H_6 as a standard; δ_{calc} = 182.4059 - δ_{abs}, in ppm).

Pyrazolyl-1,2,4-triazole 10

Table S12: Cartesian coordinates and energies of the optimized geometry of pyrazolyl-1,2,4-triazole **10** (s-*cis*-conformer).

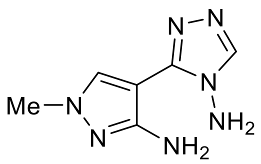

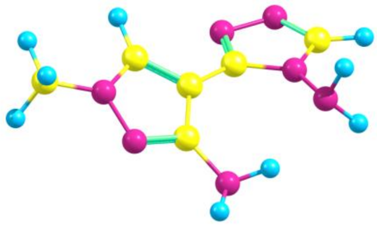

Electronic Energy =	-617.442098364 a.u.
Zero-point correction =	0.172449 (Hartree/Particle)
Thermal correction to Energy =	0.183981
Thermal correction to Enthalpy =	0.184925
Thermal correction to Gibbs Free Energy =	0.134642
Sum of electronic and zero-point Energies =	-617.269650
Sum of electronic and thermal Energies =	-617.258118
Sum of electronic and thermal Enthalpies =	-617.257173
Sum of electronic and thermal Free Energies =	-617.307457

Standard orientation:

Center Number	Atomic Number	Atomic Type	Coordinates (Angstroms) X	Y	Z
1	6	0	0.376671	-0.160068	-0.031299
2	6	0	1.095204	1.051605	-0.273276
3	7	0	2.409816	0.853740	-0.175964
4	7	0	2.557063	-0.471573	0.111488
5	6	0	1.377268	-1.099714	0.207965
6	6	0	-1.035498	-0.487462	-0.099593
7	7	0	-1.508136	-1.672640	-0.448753
8	7	0	-2.886950	-1.637329	-0.340783
9	6	0	-3.216866	-0.442425	0.066814
10	7	0	-2.095733	0.325765	0.231254
11	6	0	3.886220	-1.022070	0.311682
12	7	0	0.584120	2.282246	-0.654507
13	7	0	-2.014169	1.647017	0.701553
14	1	0	1.302691	-2.149854	0.439622
15	1	0	-4.214304	-0.071979	0.245775
16	1	0	4.492319	-0.861499	-0.581149
17	1	0	4.367696	-0.542686	1.165881
18	1	0	3.795694	-2.090367	0.499863
19	1	0	1.260213	3.031597	-0.576945
20	1	0	-0.293348	2.498526	-0.190574
21	1	0	-2.726331	2.200603	0.233072
22	1	0	-2.204711	1.655881	1.700676

Table S13: Cartesian coordinates and energies of the optimized geometry of pyrazolyl-1,2,4-triazole **10** (s-*trans*-conformer).

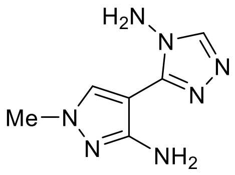

10

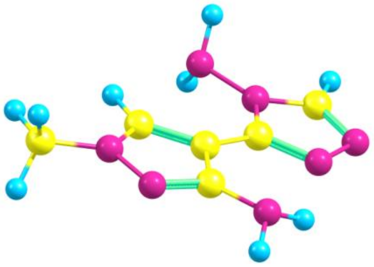

Electronic Energy =	-617.445188385 a.u.
Zero-point correction =	0.172005 (Hartree/Particle)
Thermal correction to Energy =	0.183743
Thermal correction to Enthalp =	0.184687
Thermal correction to Gibbs Free Energy =	0.133994
Sum of electronic and zero-point Energies =	-617.273184
Sum of electronic and thermal Energies =	-617.261446
Sum of electronic and thermal Enthalpies =	-617.260501
Sum of electronic and thermal Free Energies =	-617.311194

Standard orientation:

Center Number	Atomic Number	Atomic Type	Coordinates (Angstroms) X	Y	Z
1	6	0	3.691856	-1.290398	-0.048904
2	7	0	2.452919	-0.533724	-0.017235
3	6	0	1.197424	-1.006195	-0.008070
4	6	0	0.341244	0.093811	0.022779
5	6	0	1.223749	1.219076	0.033561
6	7	0	2.497452	0.832038	0.007811
7	7	0	0.864433	2.548461	0.125782
8	6	0	-1.098691	0.165480	0.002819
9	7	0	-1.977324	-0.891862	0.015814
10	6	0	-3.227668	-0.332004	-0.026629
11	7	0	-3.140104	0.968925	-0.062669
12	7	0	-1.793355	1.292763	-0.043532
13	7	0	-1.622745	-2.247832	0.063037
14	1	0	1.537674	3.202910	-0.248211
15	1	0	-0.096073	2.732735	-0.138980
16	1	0	-1.996610	-2.716275	-0.757511
17	1	0	-2.030098	-2.667076	0.894115
18	1	0	3.451124	-2.351798	-0.068049
19	1	0	4.288235	-1.070043	0.838140
20	1	0	4.264677	-1.032367	-0.941304
21	1	0	0.977621	-2.059274	-0.027257
22	1	0	-4.132961	-0.918815	-0.027343

Comment: s-*trans*-conformer of **10** is more stable than s-*cis*-conformer of **10** (ΔE = 1.94 kcal/mol, ΔG = 2.35 kcal/mol in DMSO)

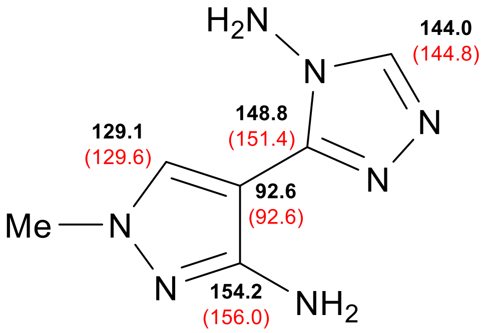

Figure S2. Experimental and calculated (numbers in red) ^{13}C chemical shifts for pyrazolyl-1,2,4-triazole **10** in DMSO solution. Calculations were performed for sp^2-atoms by the GIAO method at the PBE1PBE/6-311+G(2d,p) level of theory using the DFT B3LYP/6-311++G(d,p) optimized geometries (DMSO solution) and applying a multi-standard approach (C_6H_6 as a standard; $\delta_{calc} = 182.4059 - \delta_{abs}$, in ppm).

Calculation of thermodynamic parameters for the hydrazine promoted transformation of pyrazolopyrimidine **8** into macrocycle **5** in MeOH solution

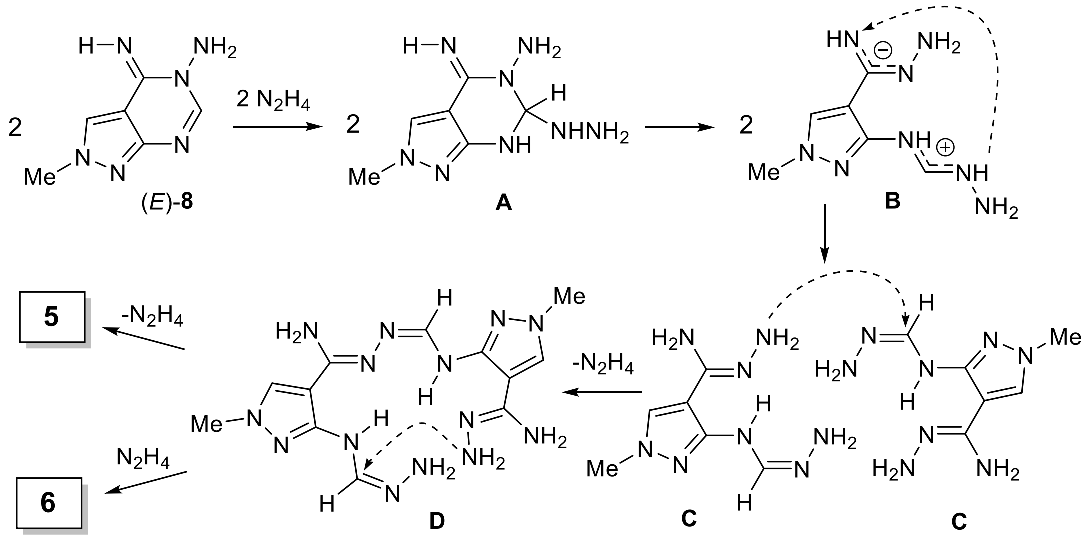

Starting compound: Pyrazolopyrimidine 8

Table S14: Cartesian coordinates and energies of the optimized geometry of *(Z)*-**8**.

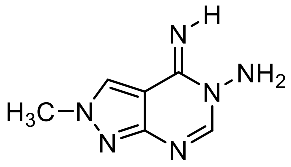

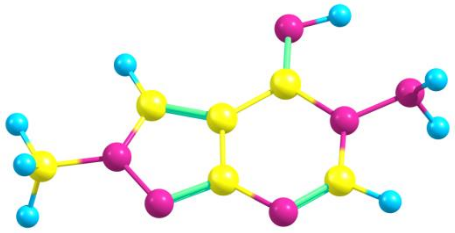

Electronic Energy =	-562.081453421 a.u.
Zero-point correction=	0.156277 (Hartree/Particle)
Thermal correction to Energy=	0.166277
Thermal correction to Enthalpy=	0.167222
Thermal correction to Gibbs Free Energy=	0.120809
Sum of electronic and zero-point Energies=	-561.925177
Sum of electronic and thermal Energies=	-561.915176
Sum of electronic and thermal Enthalpies=	-561.914232
Sum of electronic and thermal Free Energies=	-561.960645

Standard orientation:

Center Number	Atomic Number	Atomic Type	Coordinates (Angstroms) X	Y	Z
1	6	0	3.843459	0.277455	-0.000003
2	7	0	2.395084	0.124750	0.000003
3	6	0	1.470539	1.108649	0.000005
4	6	0	0.234331	0.479097	0.000004
5	6	0	0.538909	-0.904041	0.000001
6	7	0	1.856228	-1.119025	0.000004
7	6	0	-1.134452	0.945607	0.000002
8	7	0	-1.499890	2.172722	0.000001
9	7	0	-2.033972	-0.162885	-0.000002
10	6	0	-1.624112	-1.481779	-0.000002
11	7	0	-0.405515	-1.910449	-0.000002
12	7	0	-3.406692	0.149232	-0.000007
13	1	0	-3.849763	-0.232048	0.830048
14	1	0	-3.849764	-0.232075	-0.830050
15	1	0	4.081198	1.339162	0.000008
16	1	0	4.262883	-0.191417	-0.890541
17	1	0	4.262892	-0.191438	0.890519
18	1	0	1.747835	2.150045	0.000007
19	1	0	-2.519718	2.220593	-0.000003
20	1	0	-2.444315	-2.193165	-0.000007

Table S15: Cartesian coordinates and energies of the optimized geometry of *(E)*-**8**.

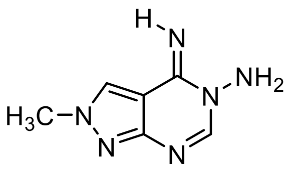

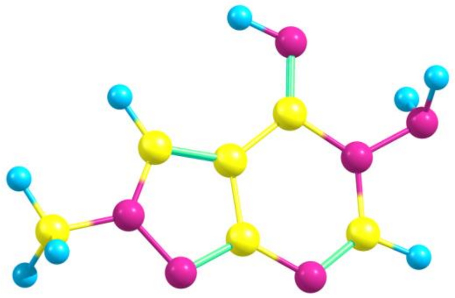

Electronic Energy =	-562.084277499 a.u.
Zero-point correction =	0.156215 (Hartree/Particle)
Thermal correction to Energy =	0.166345
Thermal correction to Enthalpy =	0.167289
Thermal correction to Gibbs Free Energy =	0.120520
Sum of electronic and zero-point Energies =	-561.928062
Sum of electronic and thermal Energies =	-561.917933
Sum of electronic and thermal Enthalpies =	-561.916988
Sum of electronic and thermal Free Energies =	-561.963758

Standard orientation:

Center Number	Atomic Number	Atomic Type	Coordinates (Angstroms) X	Y	Z
1	6	0	-3.829930	0.294115	0.000001
2	7	0	-2.382404	0.130735	0.000000
3	6	0	-1.452256	1.107224	-0.000003
4	6	0	-0.218594	0.468070	-0.000004
5	6	0	-0.534677	-0.915146	-0.000000
6	7	0	-1.853867	-1.117519	0.000003
7	6	0	1.157794	0.912190	0.000000
8	7	0	1.665803	2.092519	0.000004
9	7	0	2.039180	-0.197596	-0.000002
10	6	0	1.624068	-1.508193	0.000000
11	7	0	0.400108	-1.927328	0.000001
12	7	0	3.434322	0.010652	-0.000001
13	1	0	3.657799	0.583452	0.812050
14	1	0	3.657800	0.583456	-0.812048
15	1	0	0.928139	2.793275	0.000003
16	1	0	-4.060385	1.357357	-0.000001
17	1	0	-4.251772	-0.172305	0.890560
18	1	0	-4.251773	-0.172308	-0.890557
19	1	0	-1.727991	2.149660	-0.000006
20	1	0	2.447764	-2.212386	-0.000001

Final compound: Macrocycle 5

Table S16: Cartesian coordinates and energies of the optimized geometry of macrocycle **5**.

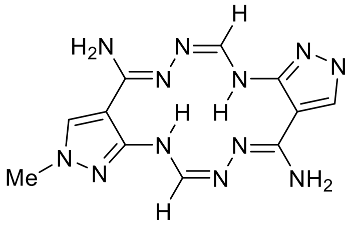

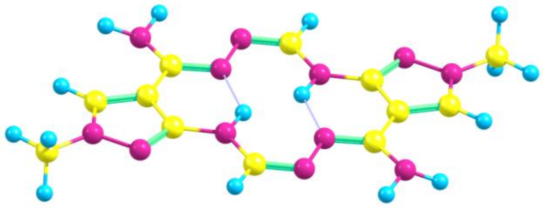

Electronic Energy =	-1124.19739243 a.u.
Zero-point correction =	0.312403 (Hartree/Particle)
Thermal correction to Energy =	0.334442
Thermal correction to Enthalpy =	0.335386
Thermal correction to Gibbs Free Energy =	0.260476
Sum of electronic and zero-point Energies =	-1123.884989
Sum of electronic and thermal Energies =	-1123.862951
Sum of electronic and thermal Enthalpies =	-1123.862006
Sum of electronic and thermal Free Energies =	-1123.936916

Standard orientation:

Center Number	Atomic Number	Atomic Type	Coordinates (Angstroms) X	Y	Z
1	7	0	0.980339	-1.603164	0.018252
2	6	0	2.259627	-1.882992	-0.008965
3	7	0	2.749930	-3.158868	0.015036
4	6	0	3.195191	-0.759804	-0.011373
5	7	0	0.137149	-2.715190	-0.002378
6	6	0	-1.118745	-2.389301	0.028061
7	7	0	-1.575842	-1.106687	0.086238
8	6	0	-2.870291	-0.633062	0.046365
9	6	0	2.870291	0.633062	-0.046364
10	7	0	1.575842	1.106687	-0.086236
11	6	0	1.118745	2.389302	-0.028061
12	7	0	-0.137149	2.715190	0.002377
13	7	0	-0.980339	1.603164	-0.018251
14	6	0	-2.259627	1.882992	0.008964
15	6	0	-3.195191	0.759804	0.011373
16	7	0	-3.953356	-1.399909	0.033322
17	7	0	-4.995535	-0.520958	-0.013494
18	6	0	-4.586983	0.759938	-0.029360
19	6	0	-6.358056	-1.025573	-0.044118
20	7	0	3.953356	1.399909	-0.033323
21	7	0	4.995535	0.520958	0.013489
22	6	0	4.586983	-0.759938	0.029358
23	6	0	6.358056	1.025573	0.044127
24	1	0	3.676837	-3.339246	-0.336445
25	1	0	2.063229	-3.882946	-0.146444
26	1	0	-0.893980	-0.350186	0.080295
27	1	0	0.893980	0.350186	-0.080292
28	1	0	-1.857083	-3.181971	0.006027
29	1	0	1.857083	3.181971	-0.006029
30	1	0	-5.299005	1.568384	-0.076482
31	1	0	-6.556982	-1.615268	0.851998
32	1	0	-7.041027	-0.178858	-0.079554
33	1	0	-6.505127	-1.648352	-0.927764
34	1	0	5.299005	-1.568384	0.076478
35	1	0	6.556972	1.615311	-0.851964
36	1	0	7.041028	0.178857	0.079514
37	1	0	6.505135	1.648310	0.927801
38	7	0	-2.749929	3.158868	-0.015041
39	1	0	-2.063229	3.882947	0.146438
40	1	0	-3.676838	3.339247	0.336437

Promoter: Hydrazine

Table S17: Cartesian coordinates and energies of the optimized geometry of hydrazine.

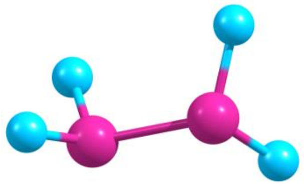

Electronic Energy =	-111.918802464 a.u.
Zero-point correction =	0.053324 (Hartree/Particle)
Thermal correction to Energy =	0.056606
Thermal correction to Enthalpy =	0.057551
Thermal correction to Gibbs Free Energy =	0.031349
Sum of electronic and zero-point Energies =	-111.865478
Sum of electronic and thermal Energies =	-111.862196
Sum of electronic and thermal Enthalpies =	-111.861252
Sum of electronic and thermal Free Energies =	-111.887454

Standard orientation:

Center Number	Atomic Number	Atomic Type	Coordinates (Angstroms) X	Y	Z
1	7	0	-0.000000	0.720383	-0.079845
2	7	0	-0.000000	-0.720383	-0.079845
3	1	0	0.255261	1.093310	0.832411
4	1	0	-0.947500	1.026029	-0.273499
5	1	0	0.947500	-1.026029	-0.273499
6	1	0	-0.255261	-1.093310	0.832411

Intermediates: Intermediate C

Table S18: Cartesian coordinates and energies of the optimized geometry of intermediate **C** (1st isomer).

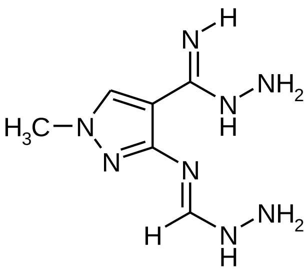

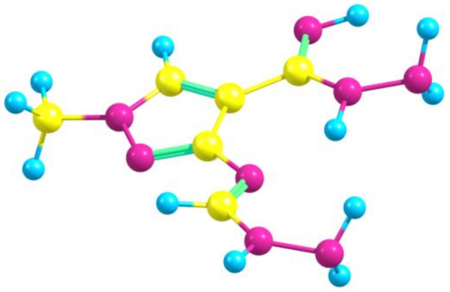

Electronic Energy =	-674.00692689 a.u.
Zero-point correction =	0.210611 (Hartree/Particle)
Thermal correction to Energy =	0.225592
Thermal correction to Enthalpy =	0.226537
Thermal correction to Gibbs Free Energy =	0.167107
Sum of electronic and zero-point Energies =	-673.796316
Sum of electronic and thermal Energies =	-673.781335
Sum of electronic and thermal Enthalpies =	-673.780390
Sum of electronic and thermal Free Energies =	-673.839820

Standard orientation:

Center Number	Atomic Number	Atomic Type	Coordinates (Angstroms) X	Y	Z
1	6	0	0.289394	-0.651909	0.036032
2	7	0	1.091381	-1.722312	0.071525
3	6	0	1.051362	0.554162	-0.012038
4	6	0	2.370403	0.119497	-0.018115
5	6	0	3.488570	-2.131216	0.066541
6	7	0	2.352521	-1.224619	0.029051
7	6	0	0.610161	1.959396	-0.040885
8	7	0	-0.753524	2.119996	-0.125860
9	7	0	-1.396795	3.358918	0.012750
10	7	0	1.485574	2.912373	-0.000927
11	7	0	-1.101275	-0.718141	0.076059
12	6	0	-1.678274	-1.828589	-0.241966
13	7	0	-3.013294	-2.000285	-0.174157
14	7	0	-3.919213	-1.029203	0.283388
15	1	0	-1.335364	1.298812	0.009665
16	1	0	-1.197898	3.949264	-0.790500
17	1	0	-1.076714	3.841395	0.851106
18	1	0	1.031209	3.824591	-0.033595
19	1	0	-3.416631	-2.885988	-0.433951
20	1	0	-3.620087	-0.715569	1.204788
21	1	0	-3.870863	-0.220109	-0.332615
22	1	0	3.291127	0.678159	-0.050037
23	1	0	3.478982	-2.704189	0.995094
24	1	0	4.403435	-1.543996	0.012335
25	1	0	3.446110	-2.816130	-0.781461
26	1	0	-1.140623	-2.711382	-0.591044

Table S19: Cartesian coordinates and energies of the optimized geometry of intermediate **C** (2nd isomer).

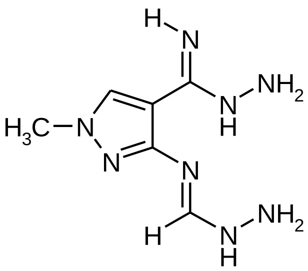

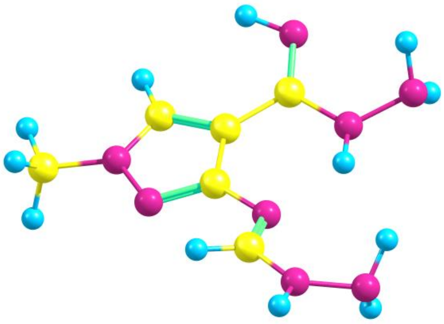

Electronic Energy =	-674.007044705 a.u.
Zero-point correction =	0.210611 (Hartree/Particle)
Thermal correction to Energy =	0.225740
Thermal correction to Enthalpy =	0.226684
Thermal correction to Gibbs Free Energy =	0.167371
Sum of electronic and zero-point Energies =	-673.796433
Sum of electronic and thermal Energies =	-673.781305
Sum of electronic and thermal Enthalpies =	-673.780360
Sum of electronic and thermal Free Energies =	-673.839673

Standard orientation:

Center Number	Atomic Number	Atomic Type	Coordinates (Angstroms) X	Y	Z
1	6	0	0.287902	-0.645987	0.048259
2	7	0	1.091576	-1.713871	0.086027
3	6	0	1.047256	0.563988	0.006299
4	6	0	2.367520	0.129614	0.013272
5	6	0	3.489284	-2.118172	0.126435
6	7	0	2.351765	-1.214708	0.057125
7	6	0	0.582190	1.963918	-0.049421
8	7	0	-0.736978	2.132120	0.295745
9	7	0	-1.404028	3.354707	0.069650
10	7	0	1.300297	2.994214	-0.366457
11	7	0	-1.101742	-0.715658	0.069799
12	6	0	-1.671083	-1.811682	-0.307616
13	7	0	-3.005096	-1.992277	-0.257669
14	7	0	-3.919362	-1.044700	0.231524
15	1	0	-1.325136	1.305033	0.269822
16	1	0	-1.046950	3.757303	-0.797242
17	1	0	-1.130130	4.005255	0.802516
18	1	0	2.247563	2.709548	-0.599417
19	1	0	-3.404606	-2.858391	-0.581780
20	1	0	-3.646951	-0.790768	1.179178
21	1	0	-3.847215	-0.199337	-0.331284
22	1	0	3.296163	0.678217	0.004503
23	1	0	4.403663	-1.532180	0.054417
24	1	0	3.447567	-2.828625	-0.699981
25	1	0	3.478857	-2.661722	1.072545
26	1	0	-1.126267	-2.673185	-0.696849

Table S20: Cartesian coordinates and energies of the optimized geometry of intermediate **C** (3rd isomer).

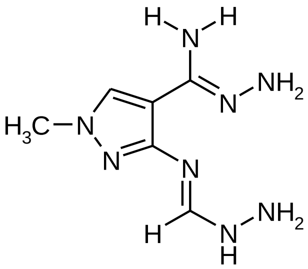

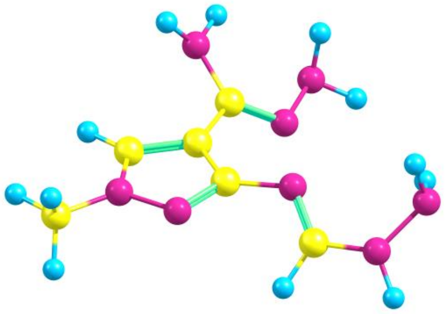

Electronic Energy =	-674.005063821 a.u.
Zero-point correction =	0.209516 (Hartree/Particle)
Thermal correction to Energy =	0.224867
Thermal correction to Enthalpy =	0.225812
Thermal correction to Gibbs Free Energy =	0.166275
Sum of electronic and zero-point Energies =	-673.795547
Sum of electronic and thermal Energies =	-673.780196
Sum of electronic and thermal Enthalpies =	-673.779252
Sum of electronic and thermal Free Energies =	-673.838789

Standard orientation:

Center Number	Atomic Number	Atomic Type	Coordinates (Angstroms) X	Y	Z
1	6	0	0.056795	-0.703755	0.064608
2	7	0	-0.324321	-1.988157	0.032555
3	6	0	-1.078424	0.160048	0.018344
4	6	0	-2.162036	-0.707512	-0.050572
5	6	0	-2.423428	-3.210758	-0.091775
6	7	0	-1.676980	-1.964569	-0.047225
7	6	0	-1.156602	1.627746	0.029660
8	7	0	-0.271535	2.323865	-0.603140
9	7	0	-0.450376	3.734456	-0.520578
10	7	0	-2.251876	2.160648	0.685036
11	7	0	1.385124	-0.325489	0.212954
12	6	0	2.284769	-1.041090	-0.363126
13	7	0	3.612273	-0.836892	-0.179975
14	7	0	4.137378	0.230180	0.572079
15	1	0	-0.447192	4.050570	0.452309
16	1	0	0.365651	4.149469	-0.955724
17	1	0	-2.652505	1.642922	1.450944
18	1	0	4.265920	-1.286392	-0.801355
19	1	0	3.633691	1.079935	0.322724
20	1	0	3.940478	0.057326	1.555443
21	1	0	-3.220015	-0.509415	-0.118364
22	1	0	-3.485485	-2.978523	-0.148716
23	1	0	-2.228569	-3.796276	0.808334
24	1	0	-2.132495	-3.790349	-0.969276
25	1	0	-2.346419	3.162012	0.741198
26	1	0	2.062687	-1.867647	-1.042292

Table S21: Cartesian coordinates and energies of the optimized geometry of intermediate **C** (4th isomer).

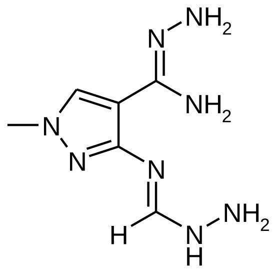

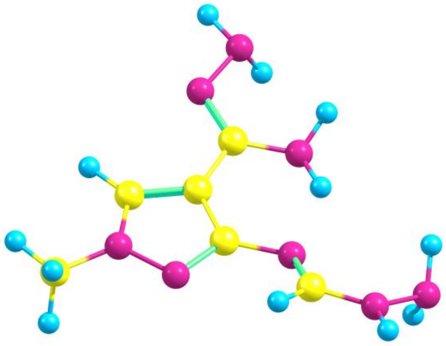

Electronic Energy =	-674.013385002 a.u.
Zero-point correction =	0.210391 (Hartree/Particle)
Thermal correction to Energy =	0.225326
Thermal correction to Enthalpy =	0.226270
Thermal correction to Gibbs Free Energy =	0.167609
Sum of electronic and zero-point Energies =	-673.802994
Sum of electronic and thermal Energies =	-673.788059
Sum of electronic and thermal Enthalpies =	-673.787115
Sum of electronic and thermal Free Energies =	-673.845776

Standard orientation:

Center Number	Atomic Number	Atomic Type	Coordinates (Angstroms) X	Y	Z
1	6	0	0.336523	0.643011	0.053196
2	7	0	0.353647	1.981964	0.065210
3	6	0	-1.001761	0.149365	0.038235
4	6	0	-1.783849	1.297187	0.032142
5	6	0	-1.299146	3.764857	0.083291
6	7	0	-0.949130	2.354491	0.042731
7	6	0	-1.470063	-1.240638	0.013995
8	7	0	-2.716297	-1.479298	-0.242649
9	7	0	-3.054761	-2.860471	-0.229176
10	7	0	-0.549191	-2.222955	0.332264
11	7	0	1.481335	-0.150424	0.078738
12	6	0	2.595868	0.362114	-0.321616
13	7	0	3.766458	-0.306270	-0.269197
14	7	0	3.919382	-1.603582	0.246956
15	1	0	-2.808883	-3.292174	0.664633
16	1	0	-4.063239	-2.912597	-0.313592
17	1	0	0.421091	-1.923335	0.281941
18	1	0	4.606673	0.129225	-0.614087
19	1	0	3.566359	-1.616262	1.201948
20	1	0	3.334345	-2.240554	-0.290269
21	1	0	-0.738071	-3.132550	-0.070327
22	1	0	-2.854528	1.417055	0.028018
23	1	0	-2.382575	3.856205	0.029282
24	1	0	-0.849339	4.283676	-0.764352
25	1	0	-0.942880	4.213770	1.012089
26	1	0	2.685508	1.367979	-0.734876

Table S22: Cartesian coordinates and energies of the optimized geometry of intermediate **C** (5th isomer).

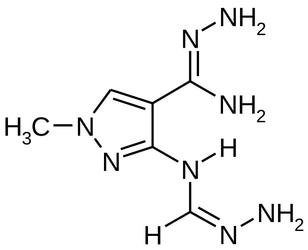

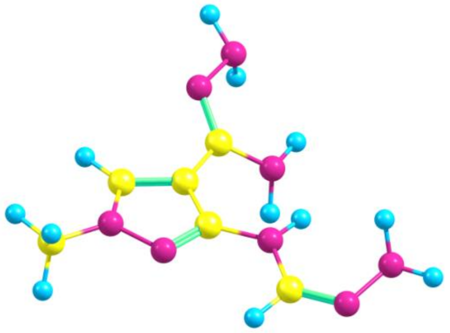

Electronic Energy =	-674.008125348 a.u.
Zero-point correction =	0.210306 (Hartree/Particle)
Thermal correction to Energy =	0.225100
Thermal correction to Enthalpy =	0.226044
Thermal correction to Gibbs Free Energy =	0.167863
Sum of electronic and zero-point Energies =	-673.797820
Sum of electronic and thermal Energies =	-673.783025
Sum of electronic and thermal Enthalpies =	-673.782081
Sum of electronic and thermal Free Energies =	-673.840263

Standard orientation:

Center Number	Atomic Number	Atomic Type	Coordinates (Angstroms) X	Y	Z
1	6	0	0.357685	0.645242	0.002987
2	7	0	0.415771	1.973343	-0.056364
3	6	0	-0.985037	0.178145	0.079336
4	6	0	-1.737178	1.345986	0.066790
5	6	0	-1.190405	3.800873	-0.022782
6	7	0	-0.882267	2.380710	-0.011195
7	6	0	-1.477524	-1.202086	0.123074
8	7	0	-2.581902	-1.491410	-0.477198
9	7	0	-3.005072	-2.840266	-0.359505
10	7	0	-0.653846	-2.121059	0.769763
11	7	0	1.504726	-0.139960	-0.056777
12	1	0	-3.098540	-3.119978	0.619345
13	1	0	-3.931963	-2.888207	-0.766220
14	1	0	-0.237675	-1.821076	1.641138
15	1	0	-0.980501	-3.076245	0.796844
16	1	0	1.360968	-1.140363	0.005672
17	6	0	2.784972	0.353126	0.072790
18	7	0	3.865907	-0.333768	-0.008359
19	7	0	3.681645	-1.719957	-0.235013
20	1	0	4.597622	-2.115580	-0.410818
21	1	0	3.116578	-1.892883	-1.068716
22	1	0	-2.804259	1.487442	0.119424
23	1	0	-2.272293	3.920845	-0.021245
24	1	0	-0.775757	4.263153	-0.919640
25	1	0	-0.770237	4.284167	0.860883
26	1	0	2.866256	1.417578	0.252694

Table S23: Cartesian coordinates and energies of the optimized geometry of intermediate **C** (6th isomer).

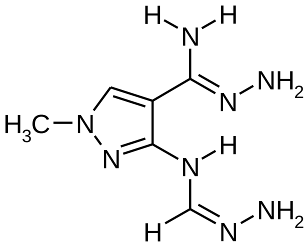

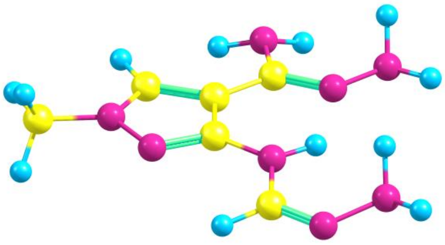

Electronic Energy =	-674.015849881 a.u.
Zero-point correction =	0.210450 (Hartree/Particle)
Thermal correction to Energy =	0.225114
Thermal correction to Enthalpy =	0.226058
Thermal correction to Gibbs Free Energy =	0.168109
Sum of electronic and zero-point Energies =	-673.805400
Sum of electronic and thermal Energies =	-673.790736
Sum of electronic and thermal Enthalpies =	-673.789792
Sum of electronic and thermal Free Energies =	-673.847741

Standard orientation:

Center Number	Atomic Number	Atomic Type	Coordinates (Angstroms) X	Y	Z
1	6	0	-0.279546	-0.692543	0.014643
2	7	0	-1.098053	-1.740019	0.040620
3	6	0	-0.994128	0.540969	0.002313
4	6	0	-2.327364	0.142945	0.025583
5	6	0	-3.507390	-2.079022	0.080009
6	7	0	-2.349271	-1.202336	0.042392
7	6	0	-0.425123	1.884572	-0.033533
8	7	0	0.864600	2.003168	0.042264
9	7	0	1.339073	3.340438	-0.026272
10	7	0	-1.281622	2.969604	-0.086819
11	7	0	1.099662	-0.811213	0.010734
12	1	0	0.997181	3.885179	0.767758
13	1	0	2.347206	3.294509	0.067447
14	1	0	-2.205558	2.797119	-0.455464
15	1	0	-0.861175	3.811197	-0.463679
16	1	0	1.579150	0.089038	-0.006095
17	6	0	1.776289	-1.991558	-0.200591
18	7	0	3.050222	-2.145840	-0.165726
19	7	0	3.798540	-0.976140	0.093741
20	1	0	4.749455	-1.265592	0.289049
21	1	0	3.456582	-0.475056	0.915808
22	1	0	-3.242197	0.714033	0.039113
23	1	0	-4.407218	-1.466856	0.065288
24	1	0	-3.492470	-2.680447	0.990404
25	1	0	-3.503533	-2.738347	-0.789255
26	1	0	1.164101	-2.860595	-0.407454

Table S24: Cartesian coordinates and energies of the optimized geometry of intermediate **C** (7th isomer).

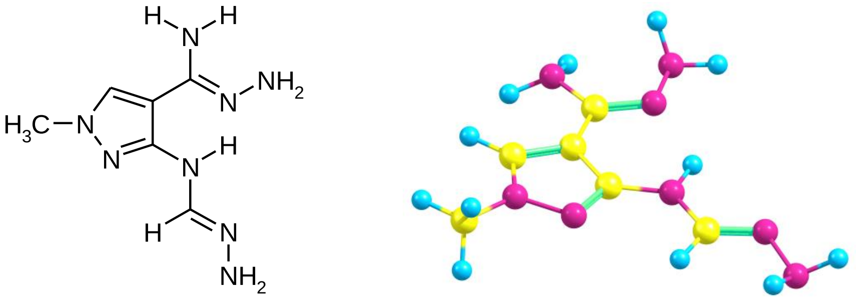

Electronic Energy =	-674.012513200 a.u.
Zero-point correction =	0.209757 (Hartree/Particle)
Thermal correction to Energy =	0.224686
Thermal correction to Enthalpy =	0.225631
Thermal correction to Gibbs Free Energy =	0.167105
Sum of electronic and zero-point Energies =	-673.802756
Sum of electronic and thermal Energies =	-673.787827
Sum of electronic and thermal Enthalpies =	-673.786883
Sum of electronic and thermal Free Energies =	-673.845408

Standard orientation:

Center Number	Atomic Number	Atomic Type	Coordinates (Angstroms) X	Y	Z
1	6	0	-0.174592	0.529012	-0.003199
2	7	0	-0.358762	1.847599	0.013453
3	6	0	1.211682	0.187739	-0.003871
4	6	0	1.850521	1.424031	0.018913
5	6	0	1.071918	3.816115	0.053098
6	7	0	0.899251	2.374422	0.024702
7	6	0	1.790328	-1.151500	-0.025102
8	7	0	0.990171	-2.168030	0.072360
9	7	0	1.636631	-3.432889	0.018036
10	7	0	3.167755	-1.278318	-0.088474
11	7	0	-1.212133	-0.377990	-0.022598
12	1	0	2.268866	-3.548309	0.812457
13	1	0	0.910536	-4.132031	0.124347
14	1	0	3.671619	-0.498475	-0.485484
15	1	0	3.482486	-2.170435	-0.452337
16	1	0	-0.921029	-1.352676	0.020229
17	6	0	-2.548793	-0.044228	-0.046959
18	7	0	-3.453770	-0.944443	0.006355
19	7	0	-4.785682	-0.505893	-0.126328
20	1	0	-5.374093	-1.143708	0.397179
21	1	0	-4.928914	0.434393	0.244306
22	1	0	2.897102	1.684281	0.041486
23	1	0	2.137298	4.038972	0.056298
24	1	0	0.612318	4.231024	0.951802
25	1	0	0.610983	4.265050	-0.828313
26	1	0	-2.757784	1.023687	-0.121785

Table S25: Cartesian coordinates and energies of the optimized geometry of intermediate **C** (8th isomer).

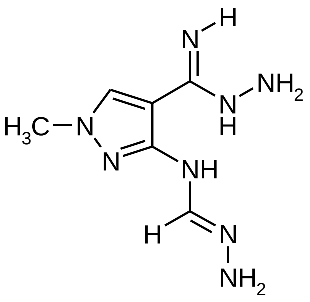

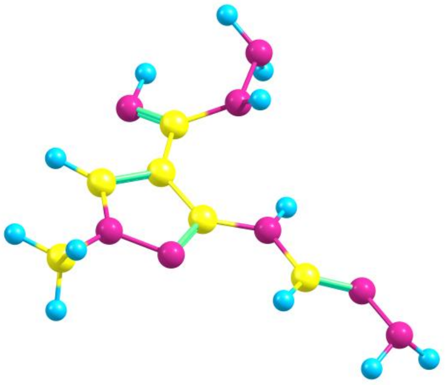

Electronic Energy =	-673.999303772 a.u.
Zero-point correction =	0.210274 (Hartree/Particle)
Thermal correction to Energy =	0.225119
Thermal correction to Enthalpy =	0.226063
Thermal correction to Gibbs Free Energy =	0.167426
Sum of electronic and zero-point Energies =	-673.789030
Sum of electronic and thermal Energies =	-673.774185
Sum of electronic and thermal Enthalpies =	-673.773241
Sum of electronic and thermal Free Energies =	-673.831877

Standard orientation:

Center Number	Atomic Number	Atomic Type	Coordinates (Angstroms) X	Y	Z
1	6	0	0.198411	0.486983	-0.005178
2	7	0	0.510312	1.779870	-0.083846
3	6	0	-1.213142	0.292218	0.062755
4	6	0	-1.723897	1.584331	0.019784
5	6	0	-0.716818	3.879133	-0.184475
6	7	0	-0.687994	2.430326	-0.075796
7	6	0	-1.999369	-0.943336	0.187408
8	7	0	1.175645	-0.494304	0.025626
9	1	0	0.882514	-1.435364	0.251118
10	6	0	2.534302	-0.242358	-0.018878
11	7	0	3.376943	-1.173895	0.205397
12	7	0	4.734438	-0.859345	0.014558
13	1	0	5.277284	-1.429832	0.652370
14	1	0	4.943221	0.121317	0.205778
15	7	0	-3.116053	-0.947695	0.825472
16	7	0	-1.387930	-2.055586	-0.397702
17	7	0	-2.036660	-3.301903	-0.485190
18	1	0	-3.560180	-1.865533	0.786136
19	1	0	-0.834902	-1.862708	-1.222293
20	1	0	-2.039736	-3.748070	0.426643
21	1	0	-2.999146	-3.199205	-0.803876
22	1	0	-2.745707	1.926366	0.040316
23	1	0	-1.751652	4.211158	-0.125012
24	1	0	-0.288538	4.192120	-1.138133
25	1	0	-0.145524	4.323341	0.631824
26	1	0	2.804539	0.782311	-0.272998

Table S26: Cartesian coordinates and energies of the optimized geometry of intermediate **C** (9th isomer).

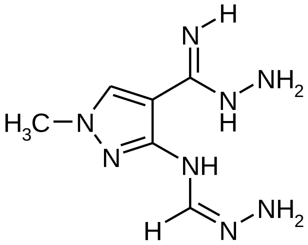

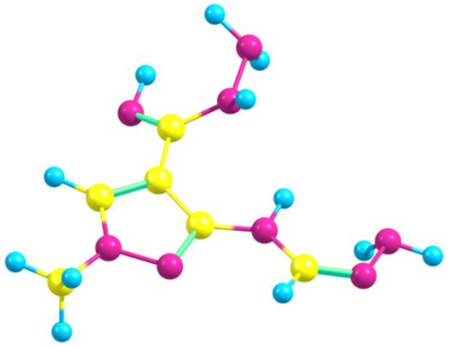

Electronic Energy =	-674.001927298 a.u.
Zero-point correction =	0.211029 (Hartree/Particle)
Thermal correction to Energy =	0.225602
Thermal correction to Enthalpy =	0.226546
Thermal correction to Gibbs Free Energy =	0.168511
Sum of electronic and zero-point Energies =	-673.790898
Sum of electronic and thermal Energies =	-673.776326
Sum of electronic and thermal Enthalpies =	-673.775381
Sum of electronic and thermal Free Energies =	-673.833416

Standard orientation:

Center Number	Atomic Number	Atomic Type	Coordinates (Angstroms) X	Y	Z
1	6	0	0.276996	-0.661756	0.022803
2	7	0	1.082474	-1.719311	-0.034904
3	6	0	1.012543	0.557241	0.064752
4	6	0	2.337784	0.140223	0.027247
5	6	0	3.484642	-2.092332	-0.136005
6	7	0	2.340843	-1.200010	-0.040826
7	6	0	0.538447	1.945986	0.166714
8	7	0	-1.104409	-0.808584	0.089293
9	1	0	-1.646610	0.042223	0.166365
10	6	0	-1.768134	-1.987233	-0.179352
11	7	0	-3.033577	-2.173354	-0.082151
12	7	0	-3.777999	-1.040671	0.328262
13	1	0	-4.728805	-1.352188	0.487343
14	1	0	-3.433811	-0.665657	1.214203
15	7	0	1.203735	2.819425	0.834205
16	7	0	-0.686842	2.161060	-0.472459
17	7	0	-1.257004	3.444681	-0.572464
18	1	0	0.767069	3.740024	0.775157
19	1	0	-0.832983	1.621792	-1.315973
20	1	0	-1.642876	3.715319	0.326614
21	1	0	-0.565979	4.138126	-0.855527
22	1	0	3.248141	0.717202	0.033510
23	1	0	4.394027	-1.497293	-0.077964
24	1	0	3.461868	-2.630977	-1.084655
25	1	0	3.464833	-2.807225	0.687438
26	1	0	-1.149077	-2.816764	-0.496158

Table S27: Cartesian coordinates and energies of the optimized geometry of intermediate **C** (10th isomer).

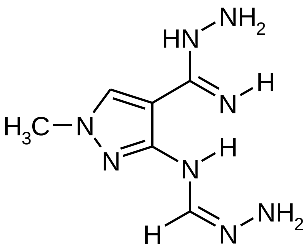

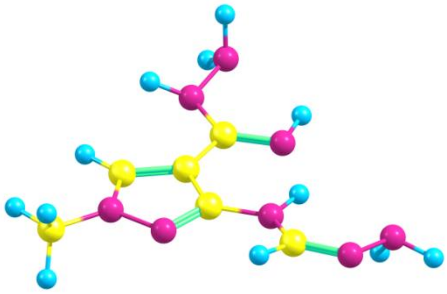

Electronic Energy =	-674.010164885 a.u.
Zero-point correction =	0.210855 (Hartree/Particle)
Thermal correction to Energy =	0.225408
Thermal correction to Enthalpy =	0.226352
Thermal correction to Gibbs Free Energy =	0.168468
Sum of electronic and zero-point Energies =	-673.799310
Sum of electronic and thermal Energies =	-673.784757
Sum of electronic and thermal Enthalpies =	-673.783813
Sum of electronic and thermal Free Energies =	-673.841697

Standard orientation:

Center Number	Atomic Number	Atomic Type	Coordinates (Angstroms) X	Y	Z
1	6	0	-0.403662	0.661707	-0.023912
2	7	0	-0.442132	1.990747	-0.006193
3	6	0	0.933367	0.168672	-0.061454
4	6	0	1.706509	1.326555	-0.069688
5	6	0	1.198289	3.788985	-0.034469
6	7	0	0.866206	2.373979	-0.030433
7	6	0	1.321394	-1.241965	-0.076568
8	7	0	-1.535376	-0.132271	-0.017628
9	1	0	-1.315066	-1.130265	-0.057362
10	6	0	-2.806224	0.328761	0.242821
11	7	0	-3.882983	-0.369332	0.210197
12	7	0	-3.716985	-1.736518	-0.099416
13	1	0	-4.635752	-2.113609	-0.298772
14	1	0	-3.139737	-1.878796	-0.930184
15	7	0	0.411043	-2.150691	-0.203069
16	7	0	2.681480	-1.494279	0.011697
17	7	0	3.090496	-2.833612	0.160551
18	1	0	0.846559	-3.073002	-0.171954
19	1	0	3.252746	-0.790335	0.461221
20	1	0	3.891112	-3.003235	-0.438404
21	1	0	3.358056	-3.022712	1.123284
22	1	0	2.774056	1.472251	-0.113358
23	1	0	2.281804	3.891977	-0.049761
24	1	0	0.800964	4.265952	0.862631
25	1	0	0.774867	4.269426	-0.917817
26	1	0	-2.889897	1.379892	0.490147

Table S28. Relative electronic (ΔE, kcal/mol) and Gibbs free energies (ΔG, kcal/mol) of various isomers (tautomers, conformers, and geometric isomers) of intermediate **C** in MeOH solution.[a]

Isomer of intermediate **C**	ΔE	ΔG
**1**	5.60	4.97
**2**	5.53	5.06
**3**	6.77	5.62
**4**	1.55	1.23
**5**	4.85	4.69
**6**	0.00	0.00
**7**	2.09	1.46
**8**	10.38	9.95
**9**	8.74	8.99
**10**	3.57	3.79

[a] Calculated using data of Tables S18-S27.

Comment: the most stable isomer of intermediate **C** is number 6:

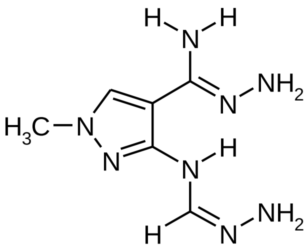

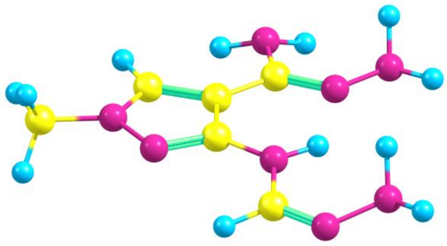

Intermediates: Intermediate A

Table S29: Cartesian coordinates and energies of the optimized geometry of intermediate **A** (1st isomer).

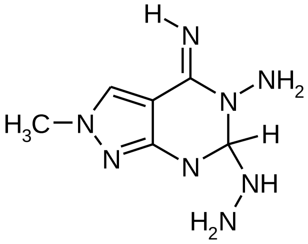

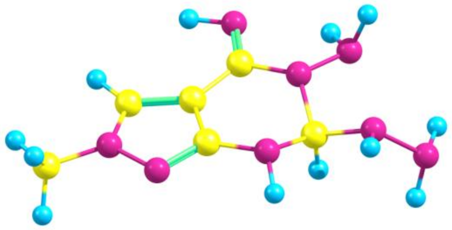

Electronic Energy =	-673.990150619 a.u.
Zero-point correction =	0.213448 (Hartree/Particle)
Thermal correction to Energy =	0.226714
Thermal correction to Enthalpy =	0.227658
Thermal correction to Gibbs Free Energy =	0.173881
Sum of electronic and zero-point Energies =	-673.776702
Sum of electronic and thermal Energies =	-673.763437
Sum of electronic and thermal Enthalpies =	-673.762493
Sum of electronic and thermal Free Energies =	-673.816270

Standard orientation:

Center Number	Atomic Number	Atomic Type	Coordinates (Angstroms) X	Y	Z
1	6	0	4.379634	-0.625427	0.143103
2	7	0	2.958369	-0.335848	0.049362
3	6	0	2.353116	0.864141	0.105987
4	6	0	0.987198	0.632869	-0.011853
5	7	0	2.069956	-1.368504	-0.097639
6	6	0	0.887576	-0.768604	-0.138155
7	6	0	-0.191101	1.486012	-0.050569
8	7	0	-1.363706	0.762925	-0.271143
9	7	0	-2.582564	1.464146	-0.048241
10	6	0	-1.483922	-0.653952	0.165677
11	7	0	-0.330709	-1.403565	-0.318736
12	1	0	-3.014715	1.624555	-0.954796
13	1	0	-2.314293	2.380506	0.312097
14	7	0	-0.253095	2.771503	0.067438
15	1	0	0.677371	3.166821	0.174730
16	7	0	-2.686483	-1.240135	-0.404032
17	7	0	-3.808384	-1.383331	0.460247
18	1	0	-0.338230	-2.368250	-0.007043
19	1	0	-2.480246	-2.130961	-0.837688
20	1	0	-4.196046	-0.454566	0.595126
21	1	0	-3.523027	-1.724310	1.380149
22	1	0	4.919784	0.313515	0.249026
23	1	0	4.574859	-1.256805	1.011253
24	1	0	4.714704	-1.137580	-0.759893
25	1	0	2.917992	1.775346	0.222259
26	1	0	-1.556843	-0.668851	1.268846

Table S30: Cartesian coordinates and energies of the optimized geometry of intermediate **A** (2nd isomer).

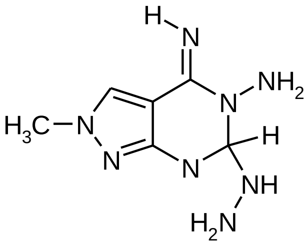

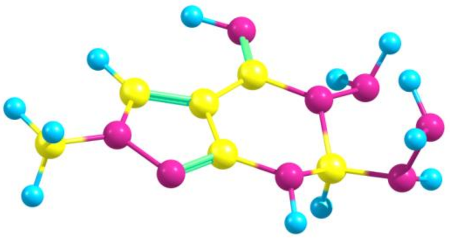

Electronic Energy =	-673.996521242 a.u.
Zero-point correction =	0.213545 (Hartree/Particle)
Thermal correction to Energy =	0.226847
Thermal correction to Enthalpy =	0.227792
Thermal correction to Gibbs Free Energy =	0.174018
Sum of electronic and zero-point Energies =	-673.782976
Sum of electronic and thermal Energies =	-673.769674
Sum of electronic and thermal Enthalpies =	-673.768730
Sum of electronic and thermal Free Energies =	-673.822503

Standard orientation:

Center Number	Atomic Number	Atomic Type	Coordinates (Angstroms) X	Y	Z
1	7	0	-2.891536	-0.260096	0.050921
2	6	0	-2.251936	0.917140	0.159876
3	6	0	-0.891174	0.649087	0.053466
4	6	0	-0.830228	-0.750638	-0.119857
5	7	0	-2.032220	-1.314236	-0.128248
6	6	0	-4.323261	-0.506346	0.083909
7	6	0	0.310672	1.466256	0.090185
8	7	0	1.490010	0.708089	0.011315
9	6	0	1.511926	-0.631511	-0.659051
10	7	0	0.372061	-1.417108	-0.211983
11	7	0	0.398626	2.750071	0.186085
12	7	0	2.675142	1.428833	-0.294249
13	1	0	0.338386	-2.333715	-0.640513
14	1	0	-0.528045	3.162853	0.261604
15	1	0	3.111309	1.705282	0.581968
16	1	0	2.393367	2.299177	-0.746043
17	7	0	2.712744	-1.380993	-0.362487
18	7	0	3.014368	-1.566869	1.024693
19	1	0	3.499893	-0.907625	-0.790034
20	1	0	2.718860	-0.751098	1.562904
21	1	0	2.458461	-2.348309	1.356201
22	1	0	-2.791456	1.840257	0.300716
23	1	0	-4.836308	0.440753	0.240045
24	1	0	-4.564019	-1.188903	0.900195
25	1	0	-4.648215	-0.944055	-0.861303
26	1	0	1.517408	-0.472369	-1.749239

Table S31: Cartesian coordinates and energies of the optimized geometry of intermediate **A** (3rd isomer).

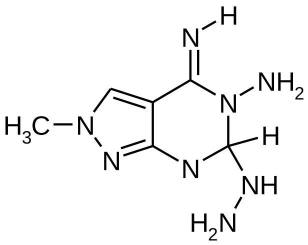

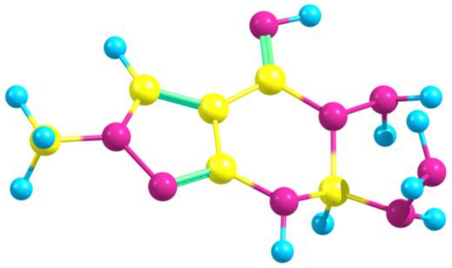

Electronic Energy =	-673.994797906 a.u.
Zero-point correction =	0.213601 (Hartree/Particle)
Thermal correction to Energy =	0.226712
Thermal correction to Enthalpy =	0.227656
Thermal correction to Gibbs Free Energy =	0.174306
Sum of electronic and zero-point Energies =	-673.781197
Sum of electronic and thermal Energies =	-673.768086
Sum of electronic and thermal Enthalpies =	-673.767142
Sum of electronic and thermal Free Energies =	-673.820492

Standard orientation:

Center Number	Atomic Number	Atomic Type	Coordinates (Angstroms) X	Y	Z
1	7	0	-2.905506	-0.246525	0.044264
2	6	0	-2.262166	0.929225	0.158484
3	6	0	-0.903486	0.656584	0.057796
4	6	0	-0.844623	-0.740223	-0.118502
5	7	0	-2.047820	-1.302810	-0.133135
6	6	0	-4.337697	-0.488887	0.068999
7	6	0	0.289569	1.481724	0.100835
8	7	0	1.484387	0.722248	-0.004049
9	6	0	1.503844	-0.634735	-0.650356
10	7	0	0.357763	-1.410286	-0.203585
11	7	0	0.247061	2.756659	0.251022
12	7	0	2.620514	1.509913	-0.277848
13	1	0	0.324804	-2.330105	-0.625341
14	1	0	1.200209	3.122042	0.228701
15	1	0	2.847695	1.503187	-1.272099
16	1	0	3.418815	1.175351	0.248961
17	7	0	2.691718	-1.392130	-0.332155
18	7	0	3.011680	-1.542448	1.051686
19	1	0	3.499286	-1.030896	-0.823721
20	1	0	2.704737	-0.723636	1.578343
21	1	0	2.477482	-2.328706	1.406933
22	1	0	-2.791373	1.857860	0.297577
23	1	0	-4.848536	0.459267	0.226364
24	1	0	-4.585402	-1.173737	0.881424
25	1	0	-4.660224	-0.921911	-0.879349
26	1	0	1.521284	-0.493179	-1.744726

Table S32. Relative electronic (ΔE, kcal/mol) and Gibbs free energies (ΔG, kcal/mol) of various isomers (geometric isomers and conformers) of intermediate **A** in MeOH solution.[a]

Isomer of intermediate **A**	ΔE	ΔG
**1**	4.00	3.91
**2**	0.00	0.00
**3**	1.08	1.26

[a] Calculated using data of Tables S29-S31.

Comment: the most stable isomer of intermediate **A** is number 2:

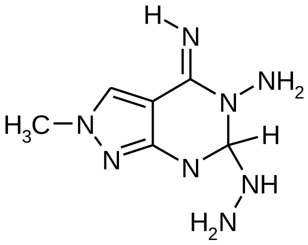

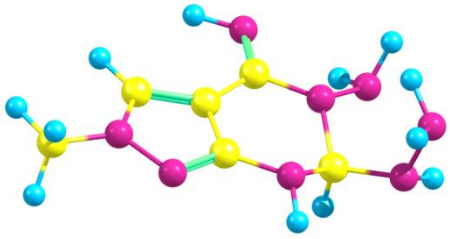

Intermediates: Intermediate D

Table S33: Cartesian coordinates and energies of the optimized geometry of intermediate **D** (1st isomer).

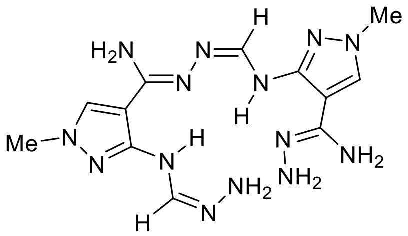

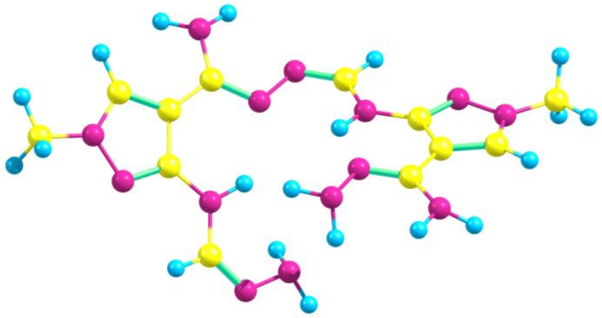

Electronic Energy =	-1236.11440651 a.u.
Zero-point correction =	0.366808 (Hartree/Particle)
Thermal correction to Energy =	0.393829
Thermal correction to Enthalpy =	0.394774
Thermal correction to Gibbs Free Energy =	0.306474
Sum of electronic and zero-point Energies =	-1235.747599
Sum of electronic and thermal Energies =	-1235.720577
Sum of electronic and thermal Enthalpies =	-1235.719633
Sum of electronic and thermal Free Energies =	-1235.807932

Standard orientation:

Center Number	Atomic Number	Atomic Type	Coordinates (Angstroms) X	Y	Z
1	7	0	-5.092677	-0.038215	-0.718057
2	6	0	-4.424343	-1.196554	-0.846858
3	6	0	-3.193541	-1.043600	-0.213923
4	6	0	-3.232586	0.296291	0.271515
5	7	0	-4.379398	0.897489	-0.029704
6	6	0	-6.412276	0.297884	-1.225499
7	7	0	-2.228627	0.913200	0.997960
8	6	0	-2.266562	2.231837	1.399209
9	7	0	-1.403576	2.821456	2.143555
10	7	0	-0.310176	2.034638	2.561224
11	6	0	-2.123829	-2.017109	-0.043879
12	7	0	-2.390825	-3.332032	-0.328827
13	7	0	-0.947775	-1.586581	0.332853
14	7	0	-0.043594	-2.617616	0.599705
15	6	0	1.198084	-2.276357	0.498743
16	7	0	1.669661	-1.066337	0.078604
17	6	0	3.009557	-0.731217	-0.023376
18	6	0	3.512921	0.494573	-0.546997
19	6	0	4.892540	0.350207	-0.439455
20	7	0	5.133915	-0.856641	0.104864
21	7	0	3.988234	-1.541082	0.371105
22	6	0	2.740893	1.624044	-1.062939
23	7	0	3.428736	2.774155	-1.384216
24	7	0	1.464946	1.460953	-1.224333
25	7	0	0.767196	2.594798	-1.723353
26	6	0	6.419800	-1.462895	0.405339
27	1	0	-1.374221	0.362465	1.059647
28	1	0	-0.604291	1.133786	2.942779
29	1	0	0.159774	2.541087	3.302030
30	1	0	-1.671803	-3.976397	-0.024952
31	1	0	-3.340066	-3.656272	-0.221669
32	1	0	1.024182	-0.360933	-0.271675
33	1	0	4.282049	2.985733	-0.891797
34	1	0	2.895918	3.579308	-1.672426
35	1	0	0.836518	3.376873	-1.068185

36	1	0	-0.212622	2.335665	-1.746450
37	1	0	-4.846838	-2.033013	-1.380514
38	1	0	-6.829678	-0.579108	-1.716606
39	1	0	-6.340516	1.116728	-1.943189
40	1	0	-7.061413	0.595298	-0.400720
41	1	0	-3.116108	2.807089	1.053305
42	1	0	1.947858	-3.013522	0.760019
43	1	0	5.700184	1.007640	-0.719246
44	1	0	7.206290	-0.759733	0.137558
45	1	0	6.484404	-1.690435	1.470453
46	1	0	6.544151	-2.382174	-0.169296

Table S34: Cartesian coordinates and energies of the optimized geometry of intermediate **D** (2nd isomer).

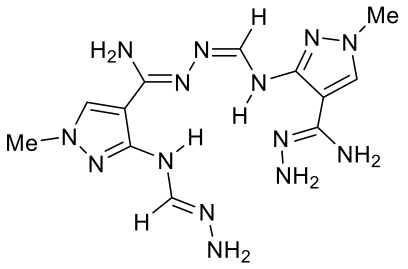

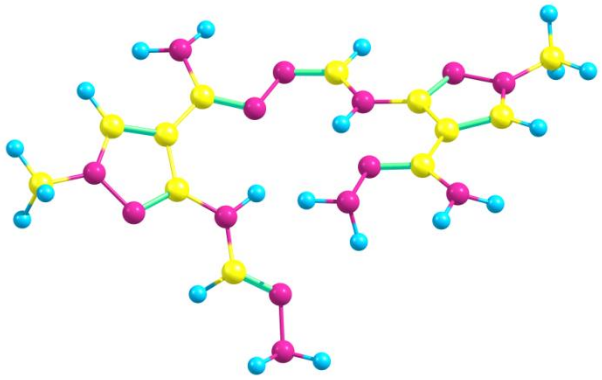

Electronic Energy =	-1236.11160350 a.u.
Zero-point correction =	0.365737 (Hartree/Particle)
Thermal correction to Energy =	0.393157
Thermal correction to Enthalpy =	0.394102
Thermal correction to Gibbs Free Energy =	0.304531
Sum of electronic and zero-point Energies =	-1235.745867
Sum of electronic and thermal Energies =	-1235.718446
Sum of electronic and thermal Enthalpies =	-1235.717502
Sum of electronic and thermal Free Energies =	-1235.807072

Standard orientation:

Center Number	Atomic Number	Atomic Type	Coordinates (Angstroms) X	Y	Z
1	7	0	-5.064301	-0.323477	-0.681135
2	6	0	-4.389280	-1.484035	-0.677814
3	6	0	-3.130358	-1.238224	-0.135021
4	6	0	-3.159973	0.159013	0.157946
5	7	0	-4.326996	0.707086	-0.174118
6	6	0	-6.407333	-0.068545	-1.172430
7	7	0	-2.130236	0.874713	0.728778
8	6	0	-2.229317	2.179750	1.163288
9	7	0	-1.228221	2.780717	1.680615
10	7	0	-1.461942	4.076772	2.178755
11	6	0	-2.048812	-2.177922	0.120197
12	7	0	-2.319206	-3.520052	0.023714
13	7	0	-0.859633	-1.701827	0.388847
14	7	0	0.058913	-2.692788	0.742330
15	6	0	1.294854	-2.330571	0.640272
16	7	0	1.745709	-1.134830	0.160151
17	6	0	3.077866	-0.772546	0.056488
18	6	0	3.555012	0.451326	-0.495886
19	6	0	4.937214	0.341601	-0.380040
20	7	0	5.203931	-0.845211	0.195646
21	7	0	4.073299	-1.549660	0.474137
22	6	0	2.757971	1.548739	-1.041853
23	7	0	3.420842	2.700856	-1.405444

24	7	0	1.484239	1.355938	-1.188943
25	7	0	0.760547	2.456399	-1.723562
26	6	0	6.501942	-1.413045	0.517884
27	1	0	-1.269226	0.350345	0.861584
28	1	0	-2.186883	4.573716	1.659716
29	1	0	-0.598479	4.600637	2.094985
30	1	0	-1.583368	-4.115428	0.382390
31	1	0	-3.260362	-3.827111	0.217318
32	1	0	1.087923	-0.458882	-0.223529
33	1	0	4.274374	2.943924	-0.928222
34	1	0	2.871117	3.486176	-1.715670
35	1	0	0.821099	3.263532	-1.098608
36	1	0	-0.214347	2.177906	-1.723283
37	1	0	-4.828429	-2.389920	-1.064569
38	1	0	-6.840121	-1.009911	-1.506095
39	1	0	-6.375028	0.632904	-2.007934
40	1	0	-7.021084	0.349479	-0.373152
41	1	0	-3.211975	2.634983	1.034069
42	1	0	2.056163	-3.033993	0.955454
43	1	0	5.731125	1.009967	-0.673088
44	1	0	7.273462	-0.701689	0.229142
45	1	0	6.568980	-1.605162	1.589851
46	1	0	6.647736	-2.347163	-0.026957

Table S35. Relative electronic (ΔE, kcal/mol) and Gibbs free energies (ΔG, kcal/mol) of various isomers of intermediate **D** in MeOH solution.[a]

Isomer of intermediate **D**	ΔE	ΔG
**1**	0.00	0.00
**2**	1.76	1.08

[a] Calculated using data of Tables S33-S34.

Comment: the most stable isomer of intermediate **A** is number 1:

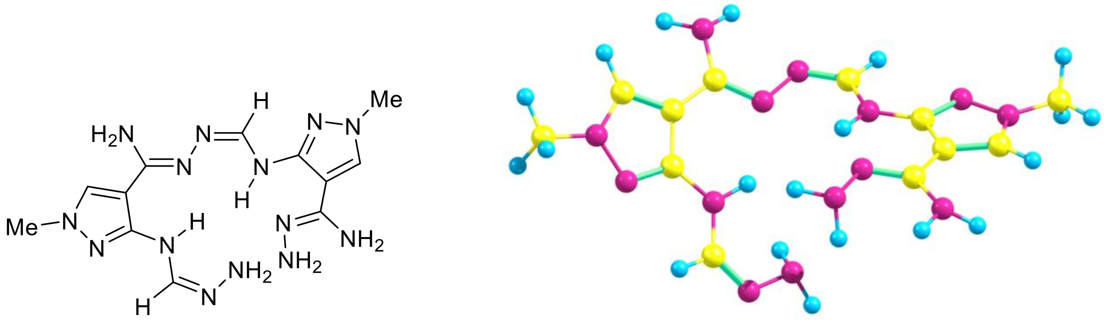

The hydrazine promoted transformation of pyrazolopyrimidine **8** into macrocycle **5** (MeOH solution)

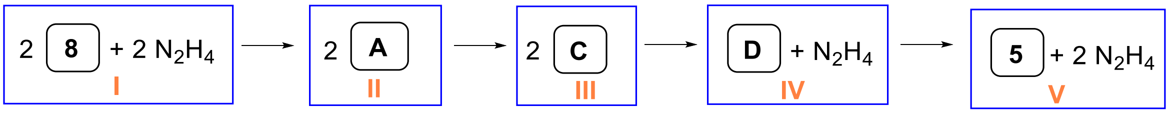

Calculations:

System I: 2×(*E*)-**8** + 2 N_2H_4
E = 2 × (-562.08427749) +2 × (-111.918802464) = -1348.006159908 a.u. (0 kcal/mol)
G = 2 × (-561.963758) + 2 × (-111.887454) = -1347.702424 a.u. (0 kcal/mol)

System II: 2×(*E*)-**A**
E = 2 × (-673.996521241) = -1347.993042482 a.u. (+8.23 kcal/mol)
G = 2 × (-673.822503) = -1347.645006 a.u. (+36.03 kcal/mol)

System III: 2×**C**
E = 2 × (-674.015849881) = -1348.031699762 a.u. (-16.03 kcal/mol)
G = 2 × (-673.847741) = -1347.695482 a.u. (+4.36 kcal/mol)

System IV: D + N_2H_4
E = (-1236.11440651) + (-111.918802464) = -1348.033208974 a.u. (-16.97 kcal/mol)
G = (-1235.807932) + (-111.887454) = -1347.695384 a.u. (+4.42 kcal/mol)

System V: 5 + 2 N_2H_4
E = (-1124.19739243) + 2 × (-111.918802464) = -1348.034997358 a.u. (-18.10 kcal/mol)
G = (-1123.936916) + 2 × (-111.887454) = -1347.711824 a.u. (-5.90 kcal/mol)

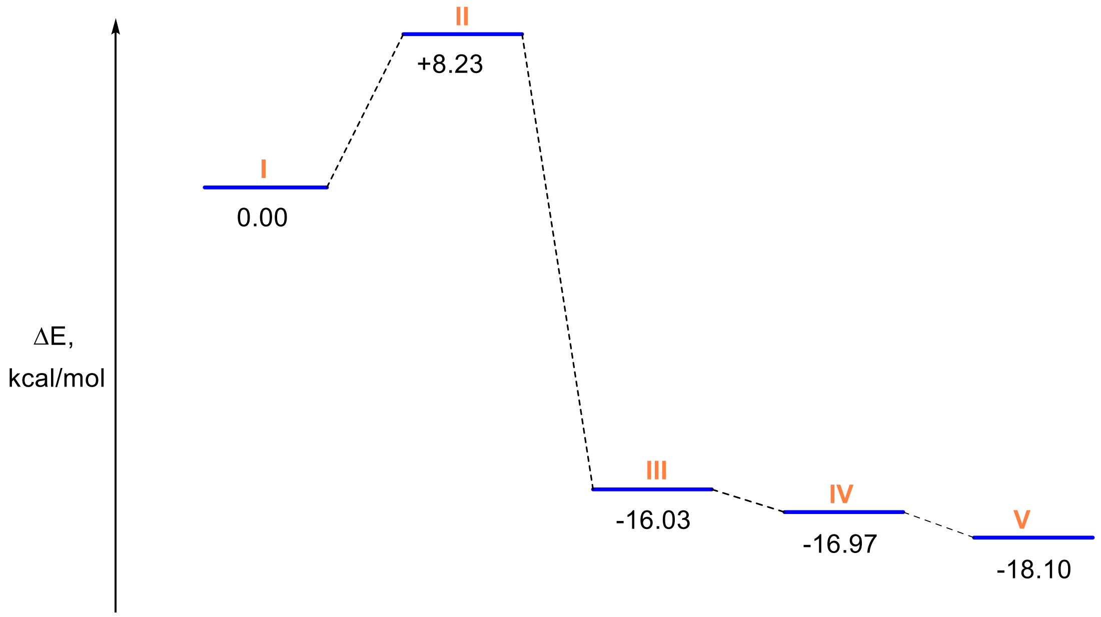

Figure S3. Energy diagram (B3LYP/6-311++G(d,p)) for the N_2H_4-promoted transformation of pyrazolopyrimidine (*E*)-**8** into macrocycle **5** in MeOH solution.

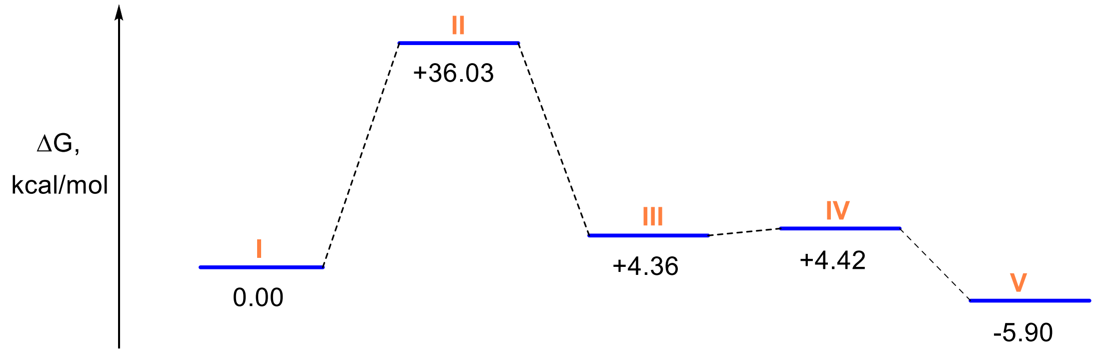

Figure S4. Gibbs free energy diagram (B3LYP/6-311++G(d,p)) for the N_2H_4-promoted transformation of pyrazolopyrimidine (*E*)-**8** into macrocycle **5** in MeOH solution. Free energies in kcal/mol at 298 K and 1 atm.

Attempt to prepare compound **11** by annulation of two 1,2,4-triazole rings onto the macrocyclic core of **5**. Synthesis of triazolo[1,5-*c*]pyrimidine **13**

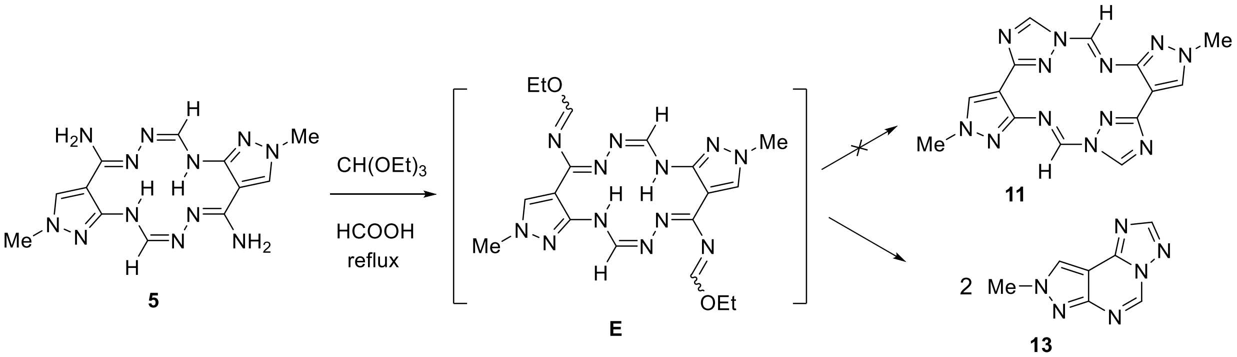

Unfortunately, our attempts to annulate two 1,2,4-triazole rings onto the macrocyclic core of **5** were unsuccessful. Particularly, reflux of macrocycle **5** in excess $CH(OEt)_3$ in the presence of HCOOH (2.5 equiv) for 8 h resulted in the formation of triazolo[1,5-*c*]pyrimidine **13** instead of the expected macrocycle **11**. The failure in the synthesis of compound **11** can be associated with difficulties in the annulation of the triazole rings due to the high conformational rigidity of macrocycle **5**. Indeed, this annulation should occur with a significant distorsion of the original geometry of macrocycle **5**. The DFT B3LYP/6-311++G(d,p) calculations in DMSO showed that the 14-membered ring in **5**, which is almost planar (see, Table S9), would change to a bowl-like configuration in compound **11** (see, Table S36) along with significant change in many bond angles. Specifically, each of the 1,2,4-triazole rings in **11** is rotated at an angle of ≈ 30 degrees relative to its coupled pyrazole ring.

Table S36: Cartesian coordinates and energies of the optimized geometry of compound **11** in DMSO.

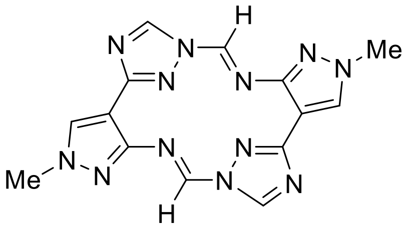

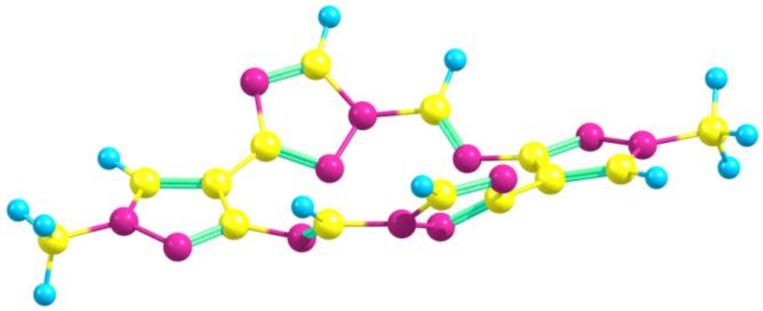

Electronic Energy =	-1197.94397409 a.u.
Zero-point correction =	0.278185 (Hartree/Particle)
Thermal correction to Energy =	0.299008
Thermal correction to Enthalpy =	0.299952
Thermal correction to Gibbs Free Energy =	0.225413
Sum of electronic and zero-point Energies =	-1197.665789
Sum of electronic and thermal Energies =	-1197.644966

Sum of electronic and thermal Enthalpies = -1197.644022
Sum of electronic and thermal Free Energies = -1197.718561

Standard orientation:

Center Number	Atomic Number	Atomic Type	Coordinates (Angstroms) X	Y	Z
1	6	0	2.093814	1.696656	-0.047809
2	7	0	0.909873	1.489054	0.488673
3	6	0	-1.286681	2.205263	-0.151166
4	7	0	-1.754103	1.233614	0.498613
5	6	0	-3.005986	0.661339	0.353373
6	6	0	-3.179574	-0.746992	0.160993
7	6	0	-2.096840	-1.706154	-0.041184
8	7	0	-0.922314	-1.520258	0.523065
9	7	0	-0.114417	-2.429774	-0.094167
10	6	0	1.282117	-2.185660	-0.153148
11	7	0	1.757617	-1.248201	0.540182
12	6	0	3.006192	-0.668564	0.368295
13	6	0	3.175028	0.736626	0.150998
14	7	0	2.127857	2.774366	-0.921490
15	6	0	0.882413	3.188056	-0.936865
16	7	0	0.114548	2.427865	-0.096776
17	6	0	-0.869333	-3.154312	-0.977767
18	7	0	-2.115455	-2.746013	-0.960741
19	6	0	4.547030	0.888186	0.029957
20	6	0	-4.551977	-0.897671	0.049469
21	7	0	4.175084	-1.302355	0.396434
22	7	0	5.103159	-0.331779	0.194387
23	6	0	6.518394	-0.667555	0.231829
24	7	0	-4.174261	1.298228	0.377645
25	7	0	-5.104443	0.326739	0.197795
26	6	0	-6.519155	0.664924	0.239850
27	1	0	-1.808522	2.868286	-0.843332
28	1	0	1.799455	-2.799651	-0.892398
29	1	0	0.481672	4.000030	-1.524742
30	1	0	-0.457425	-3.937372	-1.596381
31	1	0	5.148131	1.764670	-0.151728
32	1	0	-5.156737	-1.775092	-0.114700
33	1	0	7.091882	0.216725	-0.039714
34	1	0	6.802403	-0.991031	1.234618
35	1	0	6.725054	-1.466946	-0.480117
36	1	0	-6.796406	1.001550	1.240170
37	1	0	-6.728750	1.455495	-0.480968
38	1	0	-7.095438	-0.221910	-0.016999

The calculations also showed that stability of compound **11** is much lower than that of two equivalents of compound **13** (Table S36 vs Table S37) (ΔE = 39.89 kcal/mol and ΔG = 50.18 kcal/mol).

Table S37: Cartesian coordinates and energies of the optimized geometry of compound **13** in DMSO.

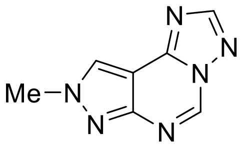

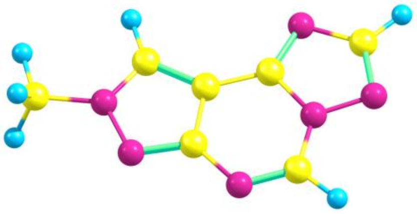

Electronic Energy =	-599.003772570 a.u.
Zero-point correction =	0.139647 (Hartree/Particle)
Thermal correction to Energy =	0.148816
Thermal correction to Enthalpy =	0.149761
Thermal correction to Gibbs Free Energy =	0.104509
Sum of electronic and zero-point Energies =	-598.864126
Sum of electronic and thermal Energies =	-598.854956
Sum of electronic and thermal Enthalpies =	-598.854012
Sum of electronic and thermal Free Energies =	-598.899263

Standard orientation:

Center Number	Atomic Number	Atomic Type	Coordinates (Angstroms) X	Y	Z
1	6	0	3.952136	-0.611928	-0.000003
2	7	0	2.525689	-0.306286	0.000000
3	6	0	1.510212	-1.195643	0.000004
4	6	0	0.344551	-0.440483	0.000005
5	6	0	0.792403	0.914818	0.000004
6	7	0	2.127287	0.983061	-0.000001
7	6	0	-1.056914	-0.656099	0.000001
8	7	0	-1.817282	0.499883	-0.000002
9	6	0	-1.297464	1.780867	-0.000002
10	7	0	-0.028255	2.017435	0.000002
11	7	0	-1.860793	-1.709843	-0.000000
12	6	0	-3.103620	-1.146243	-0.000001
13	7	0	-3.145870	0.174215	-0.000001
14	1	0	4.076124	-1.692586	0.000002
15	1	0	4.414467	-0.187463	0.890891
16	1	0	4.414462	-0.187474	-0.890906
17	1	0	1.681502	-2.259085	-0.000004
18	1	0	-2.029807	2.578957	-0.000002
19	1	0	-4.009993	-1.733333	-0.000002

Based on the above data, we proposed that macrocycle **5** reacts with $CH(OEt)_3$ initially at the NH_2 groups, but the resulting product, imidate **E**, is converted faster into compound **13** than into compound **11**. Relatively low stability and tendency to disintegration of the macrocyclic ring in **5** can be explained by its somewhat anti-aromatic character.

References

1 *Gaussian 16, Revision A.03*, Frisch, M. J.; Trucks, G. W.; Schlegel, H. B.; Scuseria, G. E.; Robb, M. A.; Cheeseman, J. R.; Scalmani, G.; Barone, V.; Petersson, G. A.; Nakatsuji, H.; Li, X.; Caricato, M.; Marenich, A. V.; Bloino, J.; Janesko, B. G.; Gomperts, R.; Mennucci, B.; Hratchian, H. P.; Ortiz, J. V.; Izmaylov, A. F.; Sonnenberg, J. L.; Williams-Young, D.; Ding, F.; Lipparini, F.; Egidi, F.; Goings, J.; Peng, B.; Petrone, A.; Henderson, T.; Ranasinghe, D.; Zakrzewski, V. G.; Gao, J.; Rega, N.; Zheng, G.; Liang, W.; Hada, M.; Ehara, M.; Toyota, K.; Fukuda, R.; Hasegawa, J.; Ishida, M.; Nakajima, T.; Honda, Y.; Kitao, O.; Nakai, H.; Vreven, T.; Throssell, K.; Montgomery, J. A., Jr.; Peralta, J. E.; Ogliaro, F.; Bearpark, M. J.; Heyd, J. J.; Brothers, E. N.; Kudin, K. N.; Staroverov, V. N.; Keith, T. A.; Kobayashi, R.; Normand, J.; Raghavachari, K.; Rendell, A. P.; Burant, J. C.; Iyengar, S. S.; Tomasi, J.; Cossi, M.; Millam, J. M.; Klene, M.; Adamo, C.; Cammi, R.; Ochterski, J. W.; Martin, R. L.; Morokuma, K.; Farkas, O.; Foresman, J. B.; Fox, D. J. Gaussian, Inc., Wallingford CT, 2016.

2 Sarotti, A. M.; Pellegrinet, S. C. *J. Org. Chem.* **2009**, *74*, 7254–7260.
